# Access to services for autistic people across Europe

**DOI:** 10.1186/s13229-025-00664-2

**Published:** 2025-06-14

**Authors:** Siti Nurnadhirah Binte Mohd Ikhsan, Rosemary Holt, Joyce Man, Tracey Parsons, Rik Schalbroeck, Amber Ruigrok, Aurélie Baranger, Carrie Allison, Mary Doherty, Katrien Van den Bosch, Jerneja Terčon, Pierre Violland, Anjuli Ghosh, James Cusack, Simon Baron-Cohen

**Affiliations:** 1 Autism Research Centre, Department of Psychiatry, University of Cambridge, Douglas House, 18B Trumpington Road, Cambridge, CB2 8 AH UK; 2 Clinical Neurodevelopmental Sciences, Institute of Education and Child Studies, Faculty of Social and Behavioural Sciences, Leiden University, Leiden, The Netherlands; 3 Division of Psychology and Mental Health, School of Health Sciences, Faculty of Biology, Medicine and Health, University of Manchester, Manchester, UK; 4 Autism-Europe, Brussels, Belgium; 5 A-Reps AIMS-2-TRIALS Consortium, Brussels, Belgium; 6 University College Dublin, Dublin, Ireland; 7 Brighton and Sussex Medical School, Brighton, UK; 8 Autistica, London, UK

**Keywords:** Autism, Services, Service access, Service barriers, Europe, Policy, Survey

## Abstract

**Background:**

Autistic communities in Europe continue to face difficulties accessing services despite increasing rates of autism diagnosis in recent years.

**Methods:**

To investigate autistic people’s access to services in Europe and reasons for unsuccessful access, we conducted the ACCESS-EU survey comprising of 2322 formally diagnosed autistic people and family carers living within the European Union (EU) and the United Kingdom (UK). The survey also examined age group (adult vs. child) and gender (male vs. female) differences in results.

**Results:**

Overall, autistic people reported access to therapy (33.38%), mental health (29.89%), educational (27.05%), medical (34.28%), financial (26.66%), needs assessment (14.90%), information/referral (14.73%), social care (14.43%), employment (7.54%), housing (6.80%), legal (3.96%), helpline (3.40%) and other services (0.26%), and most (≥ 57.61%) had waited up to 6 months from referral to access most services. Several respondents were also unable to access therapeutic (13.53%), mental health (11.90%), autism diagnostic (5.92%), needs assessment (8.32%), financial (9.62%), educational (8.10%), social care (7.39%), information/referral (6.14%), medical (7.28%), housing (5.92%), employment (5.43%), legal (3.42%), and helpline services (2.34%). Reasons cited by respondents for their unsuccessful service access included service unavailability (23.08%), service unsuitability or participant ineligibility (20.04%), long waitlists (17.42%), service unaffordability (11.80%), and rejection from service due to autism diagnosis (9.87%), along with other reasons not listed in the survey (18.42%). Significant age group and gender differences were observed for successful access to services, waiting time, unsuccessful access and reasons for unsuccessful access. Among the five most represented countries in the survey—the UK (33.33%), Spain (14.04%), Poland (13.87%), France (11.07%) and Germany (6.03%)—overall service access was most inconsistent in Poland and the UK, highest in Germany and Spain but poorest in France.

**Limitations:**

Issues related to survey presentation such as the languages in which the survey was conducted and the phrasing of some questions should be considered, as well as issues regarding subjectivity and ambiguity of data analysis such as translation of non-English responses into English.

**Conclusions:**

Our findings suggest that service access among autistic people in Europe is inconsistent. Significant improvement to current policies is required to enhance access to services across Europe.

**Supplementary Information:**

The online version contains supplementary material available at 10.1186/s13229-025-00664-2.

## Background

Studies on the prevalence of autism indicate high numbers worldwide—1 in 100 people in Europe are diagnosed [1], 1 in 36 children aged 8 years old in the US are autistic [2], and diagnosis rates in the United Kingdom (UK) have increased by 787% between 1998 and 2018 [3]. However, barriers faced by autistic people in Europe when accessing various services ranging from diagnostic and education to employment and healthcare services persist [4, 5]. Common reasons include delay in autism diagnosis, too few services, poor quality of services, cutbacks in funding for services and the lack of willingness by service providers to address autistic people’s needs [4, 6–9]. In England alone and as of June 2024, there were 187,567 cases of open suspected autism referral, and 81.20% of them with referrals open for at least 13 weeks have not had a care contact appointment recorded, with both trends increasing over 5 years [10]. The net result is a crisis in mental health, with half of autistic people suffering from anxiety or depression at any point in time [11], and, even more alarming, 34–72% of autistic people having had suicidal thoughts, 22% suicide plans and 24–47% suicide attempts compared to 9% in the general population experiencing suicidal ideation and 2–3% suicide plans, attempts behaviours [12–14].

Individual and environmental factors also have an impact on autistic people’s access to services in Europe. For example, teenagers use services more frequently than adults [15], and service access among autistic people is influenced by the educational level [16] and socioeconomic status of carers [17], language [18], financial pressures [19] and geographical location [19]. Negative social perceptions and stigma towards autism and mental health services also hamper autistic people and carers’ motivation to reach out to service providers [19, 20]. A survey by the Autism Spectrum Disorder in the European Union project (ASDEU) noted that around a fifth of autistic adults and carers in the European Union (EU) had attempted but failed to access financial services within the last 2 years at the time of survey completion [21]. For employment services, over 10% had attempted but were unable to access services [21]. In a sample of parents of 311 autistic adolescents and young adults in Poland, 82.7% of parents reported that children faced at least one barrier, such as unavailability or unaffordability of services, when attempting to use a mental health, therapy and educational service, while 93.5% said that their children were unable to access all the services they require [15].

Service availability may also vary significantly between countries across Europe. In a study on early intervention [16], children in Northern Europe received 15 h of intervention therapy every week on average, more than the 6–8 h reported by children in other parts of the continent (i.e. Southern, Eastern and Western Europe), although the percentage of autistic children accessing the service did not differ much across all regions (Northern: 38%, Southern: 54%, Eastern: 47%, Western: 40%).

In view of these challenges faced by autistic people throughout Europe in having their needs met, we conducted the Autism Services Access—EU (ACCESS-EU) study which sought to understand the experiences of autistic people in the EU and the UK when attempting to access a variety of services within their local area. An online survey was distributed to autistic communities in countries within the EU and the UK (launched just after the UK left the EU on January 31st 2020) and was open from May 2020 until May 2022. We aimed to investigate whether autistic people of all ages could access the services they were trying to reach and what their experiences were when using these services. As most countries were affected by the COVID-19 pandemic during the survey period, participants were also asked about their attempts to utilise COVID-related services. However, only data on access to non-COVID services were analysed in this paper, as well as attempts on service access made within 2 years before the completion of the ACCESS-EU survey, so as to more accurately link the data to existing policies that govern service access.

## Methods

### Survey development

The ACCESS-EU survey was designed to capture the experience of autistic people as reported by themselves and their carers when attempting to access a wide range of services that they may want and need to access. This ranged from healthcare and education to financial and housing services (see Supplementary Fig. 5 for full list and description of service types). The survey was designed with input from 18 members of the autism representatives group (A-Reps), which was established as part of the Autism Innovative Medicine Studies-2-Trials (AIMS-2-TRIALS). A-Reps are autistic people or carers, living in the EU or the UK and aged 18 or above, who are involved in shaping AIMS-2-TRIALS research. A-Reps were asked to provide feedback on an early draft of the survey and subsequently on a revised version. Consultation and feedback on the survey was also sought from representatives of Autism-Europe and the UK-based autism charity Autistica. The feedback received was incorporated into the final design of the survey and helped to ensure that the survey was accessible to autistic communities across Europe and could best assess autistic people’s experiences with services. The survey was also tested internally by the research team before it was launched.

The survey was first developed in English and then translated into French, German, Italian, and Spanish by a qualified translation agency. Czech, Polish, and Slovenian versions were produced 6 months after the survey was launched based on the top responses to a question on the survey about what other languages respondents would prefer the questionnaire to be in, as well as the languages used by sites that assisted with participant recruitment. The same company was engaged for the second phase of translation except for the Czech version which was translated by an A-Rep. Autism-Europe, researcher and clinician partners of AIMS-2-TRIALS as well as A-Reps with corresponding native languages facilitated the review of translations by native speakers to ensure that the terminology regarding autism and services was appropriate for each country.

### Participants

Autistic people who were residents of EU member states or the UK were invited to take part in the survey, and recruitment was focused on countries where a language version of the survey was available. Those who were self-diagnosed or awaiting formal diagnosis were informed at the beginning of the questionnaire that they could also participate in the study but their data would be analysed separately. Self-reporting participants were 16 years old or above whereas people aged under 16 or needing support filling in the survey could have their family carers aged 16 or older complete it for them. Participation was voluntary; respondents could withdraw from the survey at any point. The ACCESS-EU study was given a favourable opinion by the Cambridge Psychology Research Ethics Committee (reference number PRE.2019.088).

### Measures

The survey consisted of five sections: (1) demographic questions, (2) experiences of autism diagnostic service, (3) experiences of other services received, (4) unsuccessful attempts to access services, and (5) COVID-19-related service access.

Besides autism diagnostic and COVID-19-related services, we assessed participants’ experiences with 13 other types of services (see Supplementary Fig. 5). Therapy, mental health, medical and social care services were each broken down into subcategories (see Supplementary Fig. 5) for questions on waiting time, type and quality of accessed services to more precisely examine the varied types of services within each of the categories. For ‘other’ services not listed in the survey, respondents could describe up to two types of services in free-form text.

After providing consent, participants were shown the list of 13 services and were asked to indicate which of the services, if not none of them, they had ever received (see Supplementary Fig. 1 for fuller illustration of survey flow). We then asked participants, for each accessed service, how long ago the access was i.e. less than 2 years ago, 2–5 years ago, more than 5 years ago or ‘Don’t remember’. If the service had been used within 2 years before survey completion—a timeframe that covered participants’ most recent experiences upon survey completion and thus best reflected existing policies regarding service access—follow-up questions on waiting time for the service from point of referral as well as the specific type and quality of service received were asked. Participants viewed the list of 13 services again together with autism diagnostic services and were asked to select any of the services they had unsuccessfully attempted to access. For each unsuccessfully accessed service, participants were asked what they thought the reasons were for the failed access. They could choose from six options: (1) rejection from service due to autism diagnosis, (2) service unsuitability/participant ineligibility (3) service unavailability, (4) long waitlists, (5) service unaffordability, and (6) other reasons not listed in the survey. Only the latter five options were provided for autism diagnostic service, as rejection from this service due to autism diagnosis was not applicable. Participants then had to indicate how long ago these attempts were made unless they had indicated unsuccessful access to none of the services. All questions used a multiple-choice structure, most of which provided an option for participants to respond via free text if none of the options provided applied (e.g. *Have you received any of the following services? Other: ___*).

Autistic participants and carer participants were presented with slightly different versions of the questionnaire, mainly differentiating in terms of the language used (questions in the self-report version were directed to autistic participants while those in the carer version were on the autistic family members, e.g. *Age when you were first diagnosed with autism* in the self-report version versus *Age when your family member was first diagnosed with autism* in the carer version) and questions asked. The carer version had questions on the demographic background e.g. age of the carers and the autistic family members, carers’ experiences with respite care services, and whether services accessed met the carer’s needs.

### Survey distribution

The questionnaire was distributed via various local and international channels. The survey was shared via the Cambridge Autism Research Database (CARD) and the AIMS-2-TRIALS and Autism Research Centre official websites and Twitter/X accounts. Autism research centres based at European universities and hospitals within the AIMS-2-TRIALS consortium assisted with recruitment by disseminating the survey within their networks. Autism organisations and charities across the EU and the UK helped circulate the survey, in particular Autism-Europe who promoted the survey in newsletters and on social media. A-Reps also helped to disseminate the survey to their networks within their countries of residence.

### Data analysis

Data from self-reported and carer versions of the survey were combined and analysed together except for questions directed to carers (i.e. carers’ demographic information, experiences with respite care services, and whether services met the needs of the carers). In line with the focus of this paper, only data related to services accessed—successful and unsuccessful access to services, waiting time for services accessed, and reasons for unsuccessful access to services according to participants—within 2 years of completing the ACCESS-EU survey were analysed, while data for successful or unsuccessful access to no service at all was not restricted to any timeframe.

Data were analysed according to age group—those aged 16 or above were in the adult sample while those below 16 years old and represented by carers in the survey were in the children group—and gender, i.e. male, female, or other. Participants in the survey were asked which gender they identified with, and could select from ‘male’, ‘female’, ‘non-binary’, and ‘other’ categories. Participants in the male and female groups were those who had indicated ‘male’ or ‘female’ respectively regardless of whether other categories were also selected (but not both ‘male’ or ‘female’; no participant in the survey had selected both), whereas participants in the other group had indicated ‘non-binary’ or ‘other’ or both but not ‘male’ or ‘female’.

Survey entries that took less than 120 s to finish the whole survey were omitted from analysis to eliminate possible bots, as were entries that did not answer the question on which services participants could access, as this was one of the core questions.

Free-form text responses to multiple-choice questions on demographic background and on services that were successfully or unsuccessfully accessed, where the option ‘Other: ___’ was selected by respondents, were checked to see if they could be re-categorised by a member of the research team. If applicable, answers were reclassified under one of the predefined options in the questions (e.g. ‘insomnia’ as an open-text response to the question on co-occurring conditions would be categorised under the predefined option ‘sleep disorders’) or remained in the ‘other’ category. In non-English versions of the survey, Microsoft Translator and Google Translate were used to translate text responses to English before re-categorisation. If both tools were unable to disambiguate the text, Deepl was used, and if the translation provided was still vague, a native speaker in the Autism-Europe team, one of our research collaborators in the study, was consulted. A second member of the research team verified the translation and re-categorisation, and if discrepancies occurred, discussions ensued until a consensus was reached. Open-ended text responses to follow-up questions about service access and quality were not reclassified as they were more detailed and beyond this paper’s scope.

Across the whole sample of participants, distributions of responses as percentages were calculated. We carried out χ^2^ goodness-of-fit tests for differences in responses in terms of waiting time for accessed services and Cochran’s Q tests for differences in responses in terms of reasons for unsuccessful access to services. χ^2^ tests of independence were also performed to compare results between age groups—adults and children—and gender groups—male and female. For employment and legal services, age group differences were not tested as the services tend to be predominantly used and hence accessed by adults rather than children. ‘Other’ gender was not included in gender-related analyses since its small group size often rendered it insufficient in many analyses to run valid χ^2^ tests of independence. If the number of expected observations in a χ^2^ test (goodness-of-fit or test of independence) was insufficient and both variables had two categories each (i.e. 2 × 2 contingency table, e.g. successful access variable), a Fisher’s exact test would be conducted in place, but if there were more than two categories (e.g. waiting time), no tests would be performed as the SPSS package used for our analysis (version 29.0.1.0) did not have Fisher’s exact tests for bigger contingency tables. Multiple tests corrections were also not carried since the primary approach to analysing our results was to examine them within each service. The number of statistical tests conducted was thus not as many as if analyses were made across services. Meanwhile, across-service comparisons were descriptive rather than subjected to statistical tests. Multiple testing corrections might also exclude meaningful signals and might not be as applicable here where our analyses were exploratory and not predetermined. In addition to exploring the findings across countries, we compared results from five countries with the most participants in the ACCESS-EU survey i.e. the UK, Spain, Poland, France, and Germany and implemented χ^2^ tests to assess differences in responses on successful and unsuccessful access among the countries.

## Results

### Demographic background

A total of 2322 participants with a formal diagnosis of autism and from EU states (Austria, Belgium, Croatia, Cyprus, Czech Republic, Denmark, Estonia, Finland, France, Germany, Greece, Hungary, Ireland, Italy, Latvia, Lithuania, Luxembourg, Malta, the Netherlands, Poland, Portugal, Romania, Slovakia, Slovenia, Spain, Sweden) and the UK were included in the analysis (see Supplementary Table 1). Most participants were from the UK (33.33%; n = 774), Spain (14.04%; n = 326), Poland (13.87%; n = 322), France (11.07%; n = 257), and Germany (6.03%; n = 140).

Almost two-thirds of participants were adults i.e. aged 16 or above (65.20%; n = 1514; see Supplementary Fig. 2) and the remaining one-third children i.e. under 16 years of age (34.80%; n = 808). The mean age for all participants was 25.5, ranging from 1 to 78 years old (median = 21; SD = 16.6; see Supplementary Fig. 2).

Male participants constituted 62.21% of the overall sample (n = 1444; see Supplementary Fig. 2), female participants a third (33.35%; n = 774) and other-identifying participants 4.44% (n = 103). In particular, males were over-represented in the child sample, where they formed four-fifths (80.45%; n = 650) of the group, whereas male participants made up around about half (52.44%; n = 794) of the adult sample. One adult participant did not indicate their gender and was thus excluded from gender-related analyses.

Family carers who completed the questionnaire for their autistic family members made up 57.80% of the sample (n = 1342) while 42.20% were autistic self-reporters (n = 980).

Four-fifths (81.44%; n = 1891) of participants had other diagnoses of mental or physical conditions (see Supplementary Fig. 3). The most reported co-occurring conditions were anxiety disorders (31.61%; n = 734), physical conditions such as digestive problems (25.15%; n = 584) and/or intellectual or learning disability or global developmental delay (25.00%; n = 580).

These data analyses generally involved only formally diagnosed participants except when pertinent to specifically assessing people without a formal diagnosis (e.g. difficulties accessing diagnostic services). Overall, including those who were not formally diagnosed with autism, there were 2527 participants (see Supplementary Fig. 2). Almost all had a formal diagnosis (91.89%; n = 2322) whereas a small percentage (4.47%; n = 113) were self-diagnosed and awaiting an assessment by a qualified professional, and 3.64% (n = 92) were self-diagnosed but not waiting for a formal diagnosis. No participant identified as awaiting an assessment without a self-diagnosis. On average, formally diagnosed participants received their autism diagnosis at the age of 18.8 years old (range = 1–69; SD = 17.3) where 93.25% (n = 1783) had waited for a month or longer for a diagnosis, 58.32% (n = 1115) more than 6 months and 37.03% (n = 708) over 12 months.

### Successful and unsuccessful access to services

**Fig. 1:**
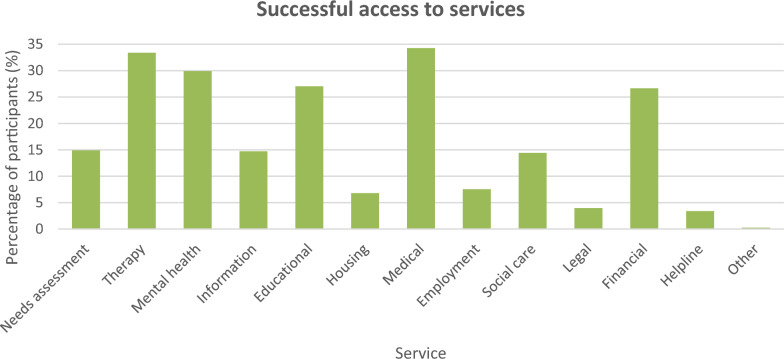
Percentage of participants indicating access to needs assessment, therapy, mental health, information/referral, educational, housing, medical, employment, social care, legal, financial and helpline services as well as other services not listed in the survey within 2 years before completing the survey, n = 2322

Within 2 years before the survey, medical services were the most accessed service among participants (34.28%; n = 796; see Fig. 1), followed by therapy (33.38%; n = 775), mental health (29.89%; n = 694), educational (27.05%; n = 628), and financial benefits/services (26.66%; n = 619). Needs assessment services were used by 14.90% of participants (n = 346) as well as information/referral (14.73%; n = 342) and social care services (14.43%; n = 335). Less than a tenth of the participants had utilised employment (7.54%; n = 175), housing (6.80%; n = 158), legal (3.96%; n = 92), helpline (3.40%; n = 79), or other services not listed in the survey (0.26%; n = 6), while 9.43% (n = 219) reported that they had received none of the listed services within the 2-year period. Results for successful access to services at any timepoint beyond the 2-year period are included in the Supplementary section (Supplementary Fig. 20).

**Fig. 2:**
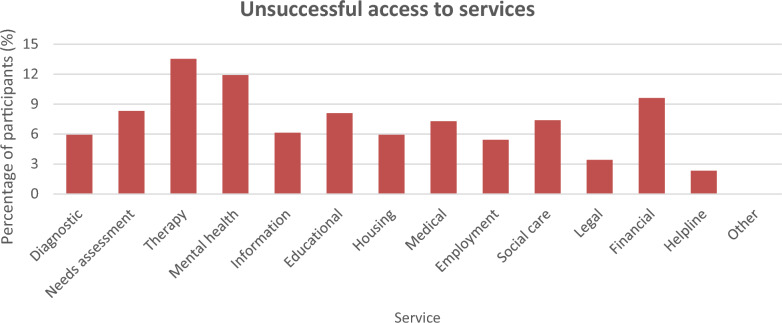
Percentage of participants indicating unsuccessful attempts at accessing autism diagnostic, needs assessment, therapy, mental health, information/referral, educational, housing, medical, employment, social care, legal, financial and helpline services as well as other services not listed in the survey within 2 years before completing the survey, n = 1840

Participants also reported unsuccessful attempts to access services in the last 2 years before survey completion. The service for which participants most commonly reported access difficulties was therapy (13.53%; n = 249; see Fig. 2), followed by mental health (11.90%; n = 219), financial benefits/services (9.62%; n = 177), needs assessment (8.32%; n = 153), and educational services (8.10%; n = 149). Participants also tried but could not access social care (7.39%; n = 136), medical (7.28%; n = 134), information/referral (6.14%; n = 113), autism diagnostic (5.92%; n = 109), housing (5.92%; n = 109), employment (5.43%; n = 100), legal (3.42%; n = 63), and helpline services (2.34%; n = 43). No participant indicated having failed to access other categories of services not listed in the survey, whereas 33.37% (n = 614) of participants reported that at any timepoint (i.e. within the last 2 years or more), they had no trouble with their attempts at accessing any of the other services. It should be noted here that participants could report both instances of successful and unsuccessful access to the same service, so the data represents participants having at least one successful or unsuccessful attempt. Data on unsuccessful access to services at any timepoint are available in the Supplementary section (Supplementary Fig. 20).

#### Autism diagnostic service

All participants in this analysis were formally diagnosed and had thus accessed an autism diagnostic service prior to completing the survey (see Supplementary Fig. 6). For people still awaiting a formal diagnosis, we asked how long they had been waiting to access an autism diagnostic service (see Supplementary Fig. 4). When analysis was performed only on known waiting times (‘unsure’ and ‘no service received’ responses omitted), 92.71% (n = 89) of participants were reported to have waited longer than 1 month, 68.76% (n = 66) longer than 6 months and 52.09% (n = 50) longer than 12 months.

Unsuccessful access to autism diagnostic services (see Fig. 2) was analysed even among formally diagnosed participants as diagnostic services could comprise of services that do not directly involve diagnosis such as consultation services. The differences in the percentage of participants reporting failed attempts to access the service were significant between adults and children (χ^2^(1, N = 1840) = 4.47, *p* = 0.04, higher among children than adults) but not between male and female groups (χ^2^(1, N = 1751) = 2.61, *p* = 0.11).

Reasons why participants had been unable to acquire diagnostic services also significantly differed (see Supplementary Fig. 6; χ^2^(4) = 32.15, *p* < 0.001). Most had indicated long waitlists (55.96%; n = 61) while over a third cited reasons other than those listed in the survey (34.86%; n = 38), under a third reported unavailability of service (31.19%, n = 34), a quarter cited unsuitability or participant ineligibility (25.69%, n = 28) and 22.02% (n = 24) unaffordability. Significant age group and gender effects were evident for reasons not listed in the survey (age group—χ^2^(1, N = 109) = 6.07, *p* = 0.01, more among adults than children; gender—χ^2^(1, N = 102) = 5.24, *p* = 0.02, more among females than males), but not other reasons (age group—service unsuitability/participant ineligibility: χ^2^(1, N = 109) = 0.18, *p* = 0.67; service unavailability: χ^2^(1, N = 109) = 1.04, *p* = 0.31; long waitlists: χ^2^(1, N = 109) = 3.80, *p* = 0.051; service unaffordability: χ^2^(1, N = 109) = 1.43, *p* = 0.23; gender—service unsuitability/participant ineligibility: χ^2^(1, N = 102) = 2.18, *p* = 0.14; service unavailability: χ^2^(1, N = 102) = 0.82, *p* = 0.37; long waitlists: χ^2^(1, N = 102) = 1.31, *p* = 0.25; service unaffordability: χ^2^(1, N = 102) = 0.10, *p* = 0.75).

#### Needs assessment

A significantly larger percentage of children indicated having had access to needs assessment services (see Fig. 1, Supplementary Fig. 7) than that of adults (χ^2^(1, N = 2322) = 27.17, *p* < 0.001), but no such differences between male and female participants (χ^2^(1, N = 2218) = 1.73, *p* = 0.19). Waiting times for accessed services (see Supplementary Fig. 7, Fig. 5d, Supplementary Table 2) were also significantly varied (χ^2^(6) = 196.98, *p* < 0.001)—excluding ‘unsure’ responses, 78.17% (n = 204) of participants reported waiting for a month or more, 34.49% (n = 90) more than 6 months and 18.01% (n = 47) more than 12 months. Waiting time was also not significantly associated with age group (χ^2^(6, N = 282) = 4.98, *p* = 0.55) or gender (χ^2^(6, N = 272) = 5.97, *p* = 0.43).

The inability to receive needs assessment services despite attempts (see Fig. 2) was unrelated to whether a participant was an adult or a child (χ^2^(1, N = 1840) = 1.04, *p* = 0.31) but was linked to gender (χ^2^(1, N = 1751) = 8.22, *p* = 0.004; more common among females than males).

Reasons for lack of access (see Supplementary Fig. 7) significantly varied (χ^2^(5) = 53.6, *p* < 0.001), with the most often cited reason being service unavailability (43.79%, n = 67). Participants also indicated reasons not listed in the survey (33.33%; n = 51), followed by long waitlists (27.45%; n = 42), service unsuitability/participant ineligibility (26.80%; n = 41), service unaffordability (15.69%; n = 24) and rejection from service due to autism diagnosis (10.46%; n = 16). Reasons reported by participants also did not significantly differ by age group (rejection from service due to autism diagnosis: Fisher’s exact test, *p* = 0.77; service unavailability: χ^2^(1, N = 153) = 0.52, *p* = 0.47; service unaffordability: χ^2^(1, N = 153) = 1.66, *p* = 0.20) except for service unsuitability/participant ineligibility (χ^2^(1, N = 153) = 6.14, *p* = 0.01, more among children than adults), long waitlists (χ^2^(1, N = 153) = 19.32, *p* < 0.001, more among children than adults) and reasons not listed in the survey (χ^2^(1, N = 153) = 17.06, *p* < 0.001, more among adults than children), nor by gender (rejection from service due to autism diagnosis: χ^2^(1, N = 146) = 2.66, *p* = 0.10; service unsuitability/participant ineligibility: χ^2^(1, N = 146) = 1.41, *p* = 0.24; service unavailability: χ^2^(1, N = 146) = 1.55, *p* = 0.21) except for long waitlists (χ^2^(1, N = 146) = 6.42, *p* = 0.01, more among males than females), service unaffordability (χ^2^(1, N = 146) = 6.77, *p* = 0.009, more among males than females) and reasons not listed in the survey (χ^2^(1, N = 146) = 3.97, *p* = 0.046, more among females than males).

#### Therapy service

Access to therapy services (see Fig. 1, Supplementary Fig. 8) was more prevalent among autistic children than autistic adults (χ^2^(1, N = 2322) = 62.14, *p* < 0.001) but differences across gender were non-significant (χ^2^(1, N = 2218) = 0.00, *p* = 0.98).

We then analysed waiting time (see Supplementary Fig. 8, Supplementary Table 2) for different subcategories of therapy service accessed (see Supplementary Fig. 8). Responses varied significantly among subservices (early intervention: χ^2^(6) = 227.10, *p* < 0.001; speech and language therapy: χ^2^(6) = 259.98, *p* < 0.001; occupational therapy: χ^2^(6) = 172.51, *p* < 0.001; physiotherapy: χ^2^(6) = 192.87, *p* < 0.001; behaviour support/therapy: χ^2^(6) = 280.02, *p* < 0.001; dietitian/nutritionist: χ^2^(6) = 61.97, *p* < 0.001; other services: χ^2^(6) = 136.92, *p* < 0.001). Omitting ‘unsure’ responses, three-quarters (74.65%; n = 106) of participants recorded successful access to early intervention within a month or more after referral, a fifth (20.42%; n = 29) more than 6 months and 7.05% (n = 10) more than 12 months, while for speech and language therapy, two-thirds (66.16%; n = 172) of participants received the service within a month or more, a quarter (25.39%; n = 66) more than 6 months and 14.24% (n = 37) more than 12 months. Occupational therapy was accessed by 68.59% (n = 131) of participants within a month or more, 28.80% (n = 55) more than 6 months and 13.09% (n = 25) more than 12 months; physiotherapy was accessed by 54.76% (n = 69) of participants within a month or more, 13.49% (n = 17) more than 6 months and 7.93% (n = 10) more than 12 months. Most participants (71.73%; n = 203) who had received behavioural support/therapy obtained it within a month or longer, 28.27% (n = 80) longer than 6 months and 13.78% (n = 39) longer than 12 months, whereas for dietitian/nutritionist services, 60.61% (n = 40) of participants acquired them within 1 month or more, 27.28% (n = 18) more than 6 months and 12.13% (n = 8) more than 12 months. Other therapy services not listed in the survey were accessed within 1 month or longer by 64.97% of participants (n = 102), longer than 6 months by 27.39% (n = 43) and longer than 12 months by 18.48% (n = 29). No analysis on the relationship between waiting time and age group as well as gender was performed for any of the therapy subservices since expected observations were insufficient in the relevant χ^2^ tests of independence.

Failed attempts to access therapy services (see Fig. 2) were reported by a significantly higher proportion for children compared to adults (χ^2^(1, N = 1840) = 8.10, *p* = 0.004) and for female participants compared to male participants (χ^2^(1, N = 1751) = 4.84, *p* = 0.03).

Reasons cited by participants for their lack of access to therapy services (see Supplementary Fig. 8) varied significantly (χ^2^(5) = 57.64, *p* < 0.001). The most common reasons specified by participants were service unavailability (42.57%; n = 106) and long waitlists (42.17%; n = 105). A third of participants indicated service unsuitability/participant ineligibility (32.53%; n = 81), the same proportion reported service unaffordability (32.13%; n = 80) and a quarter other reasons not listed in the survey (24.90%; n = 62) and while rejection from service due to autism diagnosis was reported by 16.06% (n = 40). Significant differences between adults and children were seen among participants indicating rejection from service due to autism diagnosis (χ^2^(1, N = 249) = 4.95, *p* = 0.03, more among adults than children) but not for other reasons (service unsuitability/participant ineligibility: χ^2^(1, N = 249) = 0.28, *p* = 0.60; service unavailability: χ^2^(1, N = 249) = 0.02, *p* = 0.88; long waitlists (χ^2^(1, N = 249) = 3.36, *p* = 0.07); service unaffordability: χ^2^(1, N = 249) = 0.50, *p* = 0.48); other reasons: χ^2^(1, N = 249) = 1.22, *p* = 0.27). Gender-related differences were significant only for other reasons not listed in the survey (χ^2^(1, N = 230) = 11.44, *p* < 0.001, more among females than males) but not for those which were (rejection from service due to autism: χ^2^(1, N = 230) = 0.19, *p* = 0.66; service unsuitability/participant ineligibility: χ^2^(1, N = 230) = 0.00, *p* = 0.99; service unavailability: χ^2^(1, N = 230) = 0.002, *p* = 0.96; long waitlists: χ^2^(1, N = 230) = 0.13, *p* = 0.72; service unaffordability: χ^2^(1, N = 230) = 0.12, *p* = 0.73).

#### Mental health service

Participants who had ever received mental health services (see Fig. 1, Supplementary Fig. 9) were more common among adults than among children (χ^2^(1, N = 2322) = 14.13, *p* < 0.001) as well as among females than among males (χ^2^(1, N = 2218) = 17.42, *p* < 0.001).

For participants who had accessed the various subcategories of mental health service (see Supplementary Fig. 9), the amount of time they had waited for the services differed significantly between participants (see Supplementary Fig. 9, Supplementary Table 2; crisis support: χ^2^(6) = 299.45, *p* < 0.001; outpatient: χ^2^(6) = 305.76, *p* < 0.001; inpatient: χ^2^(5) = 143.84, *p* < 0.001; other services: χ^2^(6) = 69.48, *p* < 0.001). After excluding ‘unsure’ responses to only analyse known waiting times, it was observed that crisis support was received within a month of referral or longer by 39.16% (n = 65) of participants, longer than 6 months by 13.26% (n = 22) and longer than 12 months by 7.84% (n = 13). Outpatient services were accessed within a month or longer by most participants (70.68%; n = 258), longer than 6 months by 29.59% (n = 108) and longer than 12 months by 14.52% (n = 53). To access inpatient services, 41.51% (n = 44) participants waited for a month or longer, 12.27% (n = 13) more than 6 months and 5.66% (n = 6) more than 12 months, whereas for other mental health services not listed in the survey, over two-thirds (67.47%; n = 56) of participants waited for a month or more, 27.71% (n = 23) more than 6 months and 15.66% (n = 13) more than 12 months. There was no significant association between waiting time and gender (outpatient: χ^2^(6, N = 355) = 4.66, *p* = 0.59), while no other χ^2^ tests of independence were done on age group and gender due to small sample size in service subcategories.

Failure to access mental health services in spite of attempts (see Fig. 2) exhibited no significant age group-related differences (χ^2^(1, N = 1840) = 3.32, *p* = 0.07) but was significantly more common among female than male participants (χ^2^(1, N = 1751) = 16.80, *p* < 0.001).

There were significant differences in terms of reasons indicated by participants to account for their unsuccessful attempts at service access (see Supplementary Fig. 9; χ^2^(5) = 22.50, *p* < 0.001). The most frequently cited reasons were service unsuitability/participant ineligibility (36.53%; n = 80), service unavailability (35.62%; n = 78), long waitlists (34.25%; n = 75), and other reasons not listed in the survey (31.96%; n = 70). Nearly a quarter of participants indicated rejection from service due to autism diagnosis (24.20%; n = 53) and a fifth (20.09%; n = 44) service unaffordability. Both service unavailability (χ^2^(1, N = 219) = 4.60, *p* = 0.03, more among adults than children) and reasons not listed in the survey (χ^2^(1, N = 219) = 4.83, *p* = 0.03, more among adults than children) were significantly linked to age group as well as gender (service unavailability: χ^2^(1, N = 206) = 9.42, *p* = 0.002, more among females than males; other reasons: χ^2^(1, N = 206) = 7.24, *p* = 0.01, more among females than males) while long waitlists were only related significantly to age group (χ^2^(1, N = 219) = 5.53, *p* = 0.02, more among children than adults). No significant age group-related differences were seen for the other reasons (rejection from service due to autism diagnosis: χ^2^(1, N = 219) = 2.46, *p* = 0.12; service unsuitability/participant ineligibility: χ^2^(1, N = 219) = 0.13, *p* = 0.72; service unaffordability: χ^2^(1, N = 219) = 0.14, *p* = 0.71) nor gender-related variations (rejection from service due to autism diagnosis: χ^2^(1, N = 206) = 1.19, *p* = 0.28; service unsuitability/participant ineligibility: χ^2^(1, N = 206) = 0.72, *p* = 0.40; long waitlists: χ^2^(1, N = 206) = 0.090, *p* = 0.76; service unaffordability: χ^2^(1, N = 206) = 0.02, *p* = 0.88).

#### Information/referral service

Autistic children were more likely than autistic adults to have accessed information/referral services before (see Fig. 1, Supplementary Fig. 10; χ^2^(1, N = 2322) = 18.51, *p* < 0.001), and as were male compared to female participants (χ^2^(1, N = 2218) = 4.28, *p* = 0.04). We then asked participants who had received the service how long they had waited to access it (see Supplementary Fig. 10; Supplementary Table 2). Waiting times differed significantly among participants (χ^2^(6) = 249.90, *p* < 0.001); the majority of participants (64.41%; n = 143), excluding ‘unsure’ responses, said they waited for a month or more, a fifth (20.26%; n = 45) more than 6 months and 10.80% (n = 24) more than 12 months. Differences in waiting time by age group and gender were not assessed due to invalid χ^2^ tests of independence caused by insufficient expected values.

With regards to unsuccessful access to information/referral services (see Fig. 2), no significant age group differences were found (χ^2^(1, N = 1840) = 0.09, *p* = 0.77) though there were significant gender differences (χ^2^(1, N = 1751) = 5.27, *p* = 0.02), with failure in access more common among females than males.

Reasons for failed attempts at service access (see Supplementary Fig. 10) were significantly varied (χ^2^(5) = 34.22, *p* < 0.001), with most indicating service unsuitability/participant ineligibility (41.59%; n = 47) and service unavailability (38.94%; n = 44). Almost a third of participants specified long waitlists (30.97%; n = 35) whereas 23.00% (n = 26) cited reasons not listed in the survey, 16.81% (n = 19) rejection from service due to autism diagnosis and 15.07% (n = 17) service unaffordability. All reasons indicated also did not differ significantly among age groups (rejection from service due to autism diagnosis: χ^2^(1, N = 113) = 0.06, *p* = 0.80; service unsuitability/participant ineligibility: χ^2^(1, N = 113) = 0.91, *p* = 0.34; service unavailability: χ^2^(1, N = 113) = 0.62, *p* = 0.43; service unaffordability: Fisher’s exact test, *p* = 0.26) except for long waitlists (χ^2^(1, N = 113) = 4.57, *p* = 0.03, more among children than adults) and reasons not listed in the survey (χ^2^(1, N = 113) = 5.10, *p* = 0.02, more among adults than children), nor gender groups (rejection from service due to autism diagnosis: χ^2^(1, N = 104) = 0.46, *p* = 0.50; service unsuitability/participant ineligibility: χ^2^(1, N = 104) = 1.29, *p* = 0.26; service unavailability: χ^2^(1, N = 104) = 0.19, *p* = 0.67; service unaffordability: χ^2^(1, N = 104) = 0.01, *p* = 0.94; other reasons: χ^2^(1, N = 104) = 0.31, *p* = 0.58) except for long waitlists (χ^2^(1, N = 104) = 4.95, *p* = 0.03, more among females than males).

#### Educational service

Access to educational services (see Fig. 1, Supplementary Fig. 11) was significantly more prevalent among children than adults (χ^2^(1, N = 2322) = 286.16, *p* < 0.001) and among males than females (χ^2^(1, N = 2218) = 46.70, *p* < 0.001). Waiting time for accessed educational services significantly differed across reports (χ^2^(6) = 319.20, *p* < 0.001), and without taking into account ‘unsure’ waiting time responses, most of the participants who had received the service had waited for a month or longer after referral (63.79%; n = 259; see Supplementary Fig. 11; Supplementary Table 2) while 29.80% (n = 121) waited for more than 6 months and 16.26% (n = 66) more than 12 months. Neither age group (χ^2^(6, N = 428) = 10.08, *p* = 0.12) nor gender (χ^2^(6, N = 415) = 2.41, *p* = 0.88) was significantly associated with waiting time.

Lack of access to educational services (see Fig. 2) was related to age group of participants (χ^2^(1, N = 1840) = 20.96, *p* < 0.001), affecting children more than adults, but was unrelated to gender (χ^2^(1, N = 1751) = 0.18, *p* = 0.68).

When asked about what participants thought explained their unsuccessful attempts to access the service (see Supplementary Fig. 11), reasons differed significantly (χ^2^(5) = 35.64, *p* < 0.001). However, most participants reported service unsuitability/participant ineligibility (40.27%; n = 60), service unavailability (38.26%; n = 57) and reasons not listed in the survey (30.87%; n = 46). Service unaffordability (20.81%; n = 31), rejection from service due to autism diagnosis (18.12%; n = 27) and long waitlists (18.12%; n = 27) were also cited as reasons. There was significant variability between age groups reporting long waitlists (χ^2^(1, N = 149) = 9.72, *p* = 0.002, more among children than adults) but not for other reasons (rejection from service due to autism diagnosis: χ^2^(1, N = 149) = 0.02, *p* = 0.89; service unsuitability/participant ineligibility: χ^2^(1, N = 149) = 0.37, *p* = 0.54; service unavailability: χ^2^(1, N = 149) = 1.63, *p* = 0.20; service unaffordability: χ^2^(1, N = 149) = 0.05, *p* = 0.82; other reasons: χ^2^(1, N = 149) = 0.33, *p* = 0.57), whereas gender differences were non-significant for all reasons (rejection from service due to autism diagnosis: χ^2^(1, N = 138) = 1.72, *p* = 0.19; service unsuitability/participant ineligibility: χ^2^(1, N = 138) = 0.69, *p* = 0.41; service unavailability: χ^2^(1, N = 138) = 1.49, *p* = 0.22; long waitlists: χ^2^(1, N = 138) = 0.73, *p* = 0.39; service unaffordability: χ^2^(1, N = 138) = 0.00, *p* = 0.96; other reasons: χ^2^(1, N = 138) = 0.02, *p* = 0.89).

#### Housing services

Age group (χ^2^(1, N = 2322) = 3.01, *p* = 0.08) and gender (χ^2^(1, N = 2218) = 0.00, *p* = 0.95) did not significantly impact on access to housing services (see Fig. 1, Supplementary Fig. 12). Participants who received housing services reported having waited for significantly varied lengths of time (see Supplementary Fig. 12, Supplementary Table 2; χ^2^(6) = 39.49, *p* < 0.001). Most participants who reported known waiting times (i.e. ‘unsure’ responses removed) indicated 1 month or more (79.35%; n = 73), 42.39% (n = 39) more than 6 months and 31.52% (n = 29) more than 12 months. Association analyses for age group and gender using χ^2^ tests of independence were not conducted seeing that expected counts were insufficient.

Participants who tried but were unable to acquire housing services (see Fig. 2) were not significantly different in proportion across age groups (χ^2^(1, N = 1840) = 3.08, *p* = 0.08) nor gender groups (χ^2^(1, N = 1751) = 0.44, *p* = 0.51).

Across participants, reasons for their failed access to housing services (see Supplementary Fig. 12) were significantly varied (χ^2^(5) = 34.05, *p* < 0.001), with the most common being service unavailability (43.12%; n = 47) followed by service unsuitability/participant ineligibility (30.28%; n = 33) and long waitlists (27.52%; n = 30). Reasons not listed in the survey (24.77%; n = 27), service unaffordability (15.60%; n = 17) and rejection from service due to autism diagnosis (11.00%; n = 12) were additionally reported by participants. No other age group differences (rejection due to autism diagnosis: Fisher’s exact test, *p* = 0.29; service unsuitability/participant ineligibility: χ^2^(1, N = 109) = 0.50, *p* = 0.48; service unavailability: χ^2^(1, N = 109) = 1.04, *p* = 0.31; long waitlists: χ^2^(1, N = 109) = 0.00, *p* = 0.95; service unaffordability: Fisher’s exact test, *p* = 0.21; other reasons: χ^2^(1, N = 109) = 2.84, *p* = 0.09) or gender differences (rejection from service due to autism diagnosis: χ^2^(1, N = 106) = 0.73, *p* = 0.39; service unavailability: χ^2^(1, N = 106) = 0.94, *p* = 0.33; long waitlists: χ^2^(1, N = 106) = 0.69, *p* = 0.41; service unaffordability: χ^2^(1, N = 106) = 1.49, *p* = 0.22; other reasons: χ^2^(1, N = 106) = 0.44, *p* = 0.51) were detected except for service unsuitability/participant ineligibility (χ^2^(1, N = 106) = 4.03, *p* = 0.045) which was more common among females than males.

#### Medical services

Adults were significantly more likely than children to have had access to medical services (see Fig. 1, Supplementary Fig. 13; χ^2^(1, N = 2322) = 8.09, *p* = 0.004), and it was more common among female than male participants (χ^2^(1, N = 2218) = 13.43, *p* < 0.001).

Among participants who had acquired any of the subcategories of medical service (see Supplementary Fig. 13), waiting time (see Supplementary Fig. 13; Supplementary Table 2) significantly varied for all medical subservices (GP: χ^2^(5) = 1226.54, *p* < 0.001; specialist: χ^2^(6) = 307.43, *p* < 0.001; dental: χ^2^(6) = 754.67, *p* < 0.001; emergency telephone: χ^2^(3) = 137.57, *p* < 0.001; A&E: χ^2^(3) = 246.53, *p* < 0.001; other services: χ^2^(4) = 40.54, *p* < 0.001). When ‘unsure’ waiting time responses were omitted, it was found that GP services were accessed within a month or more by 17.48% (n = 71) of participants, more than 6 months by 4.68% (n = 19) and more than 12 months by 2.46% (n = 10), whereas for specialist services, three-quarters (74.50%: n = 187) of participants obtained them within a month or longer, a quarter (25.10%; n = 63) 6 months or longer, and 11.95% (n = 30) longer than 12 months. Half (50.34%; n = 224) of participants accessed dental services within 1 month or more, 12.36% (n = 55) more than 6 months and 3.82% (n = 17) more than 12 months, compared to emergency telephone services which were received within a month or more by 5.46% (n = 3) of participants and more than 6 months by 1.82% (n = 1; no participant accessed the service within more than 12 months). Equal numbers of participants (2.24%; n = 2) who had accessed A&E services had waited for a month or longer, more than 6 months and more than 12 months, whilst for other medical services not listed in the survey, 28.00% (n = 7) of participants waited for a month or more, 12.00% (n = 3) more than 6 months and 4.00% (n = 1) more than 12 months. χ^2^ tests of independence seeking to identify any relationship between waiting time and age group as well as gender were not administered because of low expected numbers of observations.

Unsuccessful attempts to obtain medical services (see Fig. 2) did not exhibit significant age group-related (χ^2^(1, N = 1840) = 0.06, *p* = 0.81) nor gender-related differences (χ^2^(1, N = 1751) = 2.09, *p* = 0.15).

Differences in reasons indicated by participants for their unsuccessful access (see Supplementary Fig. 13) were seen to not be significant (χ^2^(5) = 8.26, *p* = 0.14). The most often reported reasons were long waitlists (34.33%; n = 46), reasons not listed in the survey (32.09%; n = 43), service unavailability (28.36%; n = 38) as well as service unsuitability/participant ineligibility (27.61%; n = 37) and service unaffordability (27.61%; n = 37). Almost a quarter of participants cited rejection from service due to autism diagnosis (19.40%; n = 26). Several reasons indicated were related significantly to age group, namely service unsuitability/participant ineligibility: χ^2^(1, N = 134) = 5.07, *p* = 0.02, more among children than adults), long waitlists (χ^2^(1, N = 134) = 8.84, *p* = 0.003, more among children than adults) and reasons not listed in the survey (χ^2^(1, N = 134) = 4.78, *p* = 0.03, more among adults than children), but not for other reasons (rejection from service due to autism diagnosis (χ^2^(1, N = 134) = 3.29, *p* = 0.07; service unavailability: χ^2^(1, N = 134) = 0.14, *p* = 0.71; service unaffordability: χ^2^(1, N = 134) = 0.34, *p* = 0.56). Significant differences due to gender were absent elsewhere (rejection from service due to autism diagnosis: χ^2^(1, N = 130) = 2.86, *p* = 0.09; service unsuitability/participant ineligibility: χ^2^(1, N = 130) = 1.77, *p* = 0.18; service unavailability: χ^2^(1, N = 130) = 1.08, *p* = 0.30; long waitlists: χ^2^(1, N = 130) = 0.50, *p* = 0.48; service unaffordability: χ^2^(1, N = 130) = 0.58, *p* = 0.45; reasons not listed in the survey (χ^2^(1, N = 130) = 2.45, *p* = 0.12).

#### Employment services

Successful access to employment services (see Fig. 1, Supplementary Fig. 14) showed significant differences across gender (χ^2^(1, N = 2218) = 6.82, *p* = 0.009, more among females than males). Age group analyses were not conducted as employment services are generally used more by adults than children; indeed, it was mostly adults who had indicated successful (98.29%, n = 172 vs. 1.71%, n = 3 children) or unsuccessful access (93.00%, n = 93 vs. 7.00%, n = 7 children) to the service. Across participants who had accessed the service, amount of waiting time differed significantly (χ^2^(6) = 130.20, *p* < 0.001; see Supplementary Fig. 14; Supplementary Table 2). Upon removing ‘unsure’ waiting times, we saw that 60.00% (n = 66) reporting waiting times of 1 month or more, 19.08% (n = 21) more than 6 months and 10.00% (n = 11) more than 12 months. It was not possible to derive gender differences as expected counts were insufficient in χ^2^ tests of independence.

As for failed attempts to access employment services (see Fig. 2), no significant gender effects were established (χ^2^(1, N = 1751) = 0.72, *p* = 0.40).

Reasons for unsuccessful access to employment services (see Supplementary Fig. 14) differed significantly across participants (χ^2^(5) = 50.66, *p* < 0.001), where most indicated service unavailability (44.00%; n = 44), service unsuitability/participant ineligibility (39.00%; n = 39) and/or reasons not listed in the survey (28.00%; n = 28). Other reasons included rejection from service due to autism diagnosis (18.00%; n = 18), long waitlists (13.00%; n = 13) and service unaffordability (9.00%; n = 9). No significant association was derived between reasons specified by participants and gender (rejection from service due to autism diagnosis: χ^2^(1, N = 93) = 0.02, *p* = 0.90; service unsuitability/participant ineligibility: χ^2^(1, N = 93) = 0.22, *p* = 0.64; service unavailability: χ^2^(1, N = 93) = 3.06, *p* = 0.08; long waitlists: Fisher’s exact test, *p* = 0.53; service unaffordability: Fisher’s exact test, *p* = 1.00; other reasons: χ^2^(1, N = 93) = 1.11, *p* = 0.29).

#### Social care services

Responses indicating successful access to social care services (see Fig. 1, Supplementary Fig. 15) showed a significant association with participants’ age group (χ^2^(1, N = 2322) = 10.05, *p* = 0.002, more among adults than children) though proportions of males and females did not significantly differ (χ^2^(1, N = 2218) = 0.00, *p* = 0.96).

For participants who had used social care services, the amount of time they had waited (see Supplementary Fig. 15; Supplementary Table 2) varied significantly for all subcategories of service (see Supplementary Fig. 15; respite care: χ^2^(6) = 27.65, *p* < 0.001; peer support: χ^2^(5) = 29.73, *p* < 0.001; support group: χ^2^(6) = 81.54, *p* < 0.001; support with daily living skills: χ^2^(6) = 41.60, *p* < 0.001; advocacy: χ^2^(6) = 31.63, *p* < 0.001; other services: χ^2^(6) = 58.34, *p* < 0.001). Three-quarters (75.51%; n = 37; excluding ‘unsure’ responses) of carer participants said they had waited for 1 month or longer to access respite care services, 40.81% (n = 20) longer than 6 months and 20.40% (n = 10) longer than 12 months. Peer support services were accessed within a month or longer by half of participants (50.00%; n = 15), longer than 6 months by 16.67% (n = 5) and longer than 12 months by 6.67% (n = 2), whereas support group services were received by over half of participants (52.94%; n = 27) within a month or longer, 13.72% (n = 7) more than 6 months and 7.84% (n = 4) more than 12 months. Almost three-quarters (74.24%; n = 49) of participants waited for a month or longer to access support for daily living skills services, 34.84% (n = 23) longer than 6 months and nearly a quarter (24.24%; n = 16) longer than 12 months, while over half (57.14%; n = 20) of participants waited for a month or more for advocacy services, a fifth (25.70%; n = 9) more than six months and 17.14% (n = 6) more than 12 months. For other social care services not listed in the survey, 72.42% (n = 63) of participants waited for a month or longer, a third (33.34%; n = 29) longer than 6 months and 17.24% (n = 15) longer than 12 months. No χ^2^ tests of independence on age group and gender were carried out as expected observations were insufficient.

Reports of unsuccessful attempts to receive social care services (see Fig. 2) were not significantly associated with age group (χ^2^(1, N = 1840) = 0.21, *p* = 0.64) nor with gender (χ^2^(1, N = 1751) = 2.54, *p* = 0.11).

Participants also differed significantly as to reasons for their lack of access to the service (see Supplementary Fig. 15; χ^2^(5) = 47.64, *p* < 0.001), but the majority cited service unavailability (38.24%; n = 52), reasons not listed in the survey (35.29%; n = 48) and/or service unsuitability/participant ineligibility (28.68%; n = 39). Long waitlists were reported by 21.32% (n = 29) of participants, service unaffordability by 11.03% (n = 15) and rejection from service due to autism diagnosis by 10.29% (n = 14). Rejection from service due to autism diagnosis was more likely to be experienced by females than males (χ^2^(1, N = 128) = 5.51, *p* = 0.02), but no other significant age group (rejection from service due to autism diagnosis: Fisher’s exact test, *p* = 0.07; service unsuitability/participant ineligibility: χ^2^(1, N = 136) = 1.39, *p* = 0.24; service unavailability: χ^2^(1, N = 136) = 0.56, *p* = 0.46; long waitlists: χ^2^(1, N = 136) = 0.10, *p* = 0.75; service unaffordability: Fisher’s exact test, *p* = 0.13; other reasons: χ^2^(1, N = 136) = 0.49, *p* = 0.48) or gender differences (service unsuitability/participant ineligibility: χ^2^(1, N = 128) = 1.69, *p* = 0.19; service unavailability: χ^2^(1, N = 128) = 0.49, *p* = 0.48; long waitlists: χ^2^(1, N = 128) = 0.37, *p* = 0.54; service unaffordability: χ^2^(1, N = 128) = 3.54, *p* = 0.06; other reasons: χ^2^(1, N = 128) = 0.14, *p* = 0.71) were noted.

#### Legal services

The difference in proportion between males and females indicating access to legal services (see Fig. 1, Supplementary Fig. 16) was not significant (χ^2^(1, N = 2218) = 1.26, *p* = 0.26). Because it is primarily adults and not children who use legal services—as also observed in our sample where more adults than children reported successful (85.87%, n = 79 vs. 14.13%, n = 13) or unsuccessful access (69.84%, n = 44 vs. 30.16%, n = 19) to the service—age group analyses were not performed. Among those who had accessed the service, waiting times significantly differed (see Supplementary Fig. 16, Supplementary Table 2; χ^2^(6) = 217.45, *p* < 0.001). When ‘unsure’ waiting time responses were omitted, a quarter of participants (25.68%; n = 19) were seen to have indicated a waiting time of 1 month or more, 9.46% (n = 7) more than 6 months and 6.76% (n = 5) more than 12 months. Because expected observations were not enough, χ^2^ tests of independence on age group and gender were not performed.

With respect to unsuccessful attempts to access legal services (see Fig. 2), no significant relationship was ascertained with age group (χ^2^(1, N = 1840) = 0.00, *p* = 0.96) or gender (χ^2^(1, N = 1751) = 0.03, *p* = 0.87).

There were significant variations as to what participants believed were reasons behind their unsuccessful access to the service (see Supplementary Fig. 16; χ^2^(5) = 43.49, *p* < 0.001), with half (50.79%; n = 32) saying it was service unavailability and almost the same proportion (47.62%; n = 30) service unaffordability. Participants also indicated service unsuitability/participant ineligibility (22.22%; n = 14), reasons not listed in the survey (20.63%; n = 13), long waitlists (15.87%; n = 10) and rejection from service due to autism diagnosis (11.11%; n = 7). Participants’ responses also did not differ as a function of age group (rejection from service due to autism diagnosis: Fisher’s exact test, *p* = 0.66; service unsuitability/participant ineligibility: Fisher’s exact test, *p* = 1.00; service unavailability: χ^2^(1, N = 63) = 0.04, *p* = 0.85; long waitlists: Fisher’s exact test, *p* = 0.71; service unaffordability: χ^2^(1, N = 63) = 2.81, *p* = 0.09; other reasons: Fisher’s exact test, *p* = 0.74) or gender (rejection from service due to autism diagnosis: Fisher’s exact test, *p* = 0.09; service unsuitability/participant ineligibility: Fisher’s exact test, *p* = 0.75; service unavailability: χ^2^(1, N = 60) = 0.77, *p* = 0.38; long waitlists: Fisher’s exact test, *p* = 0.73; service unaffordability: χ^2^(1, N = 60) = 1.61, *p* = 0.21; other reasons: χ^2^(1, N = 60) = 0.07, *p* = 1.00).

#### Financial benefits/services

The likelihood of receiving financial benefits/services (see Fig. 1, Supplementary Fig. 17) was greater among children than adults (χ^2^(1, N = 2322) = 31.10, *p* < 0.001) and among males than females (χ^2^(1, N = 2218) = 14.08, *p* < 0.001). Participants who had accessed the service had waited for significantly different lengths of time after referral (see Supplementary Fig. 17, Supplementary Table 2; χ^2^(6) = 226.14, *p* < 0.001). Discarding ‘unsure’ waiting responses to only study known waiting times, it was revealed that the majority of participants (80.73%; n = 331) waited for a month or more, 40.97% (n = 168) more than 6 months and nearly a quarter (24.14%; n = 99) more than 12 months. Significant variability was also present across age groups (χ^2^(6, N = 445) = 29.05, *p* < 0.001)—more among adults than children indicated < 1 month or ‘unsure’, while more among children than adults indicated 6–12 months, 12–18 months or 18 months-3 years. Gender, however, had no significant relationship with waiting time (χ^2^(6, N = 421) = 4.56, *p* = 0.60).

Lack of access to financial benefits/services (see Fig. 2) was not significantly linked to age group (χ^2^(1, N = 1840) = 0.70, *p* = 0.40) but varied significantly by gender of participants (χ^2^(1, N = 1751) = 6.83, *p* = 0.009, more among females than males).

Significant differences were seen in terms of reasons for participants’ unsuccessful attempts to acquire the service (see Supplementary Fig. 17; χ^2^(5) = 81.72, *p* < 0.001), where the most often cited reasons were those not listed in the survey (40.11%; n = 71) and service unsuitability/participant ineligibility (37.85%; n = 67) while 28.25% (n = 50) indicated service unavailability, 16.38% (n = 29) rejection from service due to autism diagnosis, 14.12% (n = 25) long waitlists and 6.78% (n = 12) service unaffordability. A significant association with age group was observed for long waitlists (χ^2^(1, N = 177) = 6.00, *p* = 0.01, more among children than adults) but not for other reasons (rejection from service due to autism diagnosis: χ^2^(1, N = 177) = 1.89, *p* = 0.17; service unsuitability/participant ineligibility: χ^2^(1, N = 177) = 1.51, *p* = 0.22; service unavailability: χ^2^(1, N = 177) = 0.00, *p* = 0.95; service unaffordability: Fisher’s exact test, *p* = 0.10; reasons not listed in the survey: χ^2^(1, N = 177) = 2.55, *p* = 0.11). All reasons also exhibited no gender differences (rejection from service due to autism diagnosis: χ^2^(1, N = 167) = 0.52, *p* = 0.47; service unsuitability/participant ineligibility: χ^2^(1, N = 167) = 0.52, *p* = 0.47; service unavailability: χ^2^(1, N = 167) = 0.46, *p* = 0.50; long waitlists: χ^2^(1, N = 167) = 0.01, *p* = 0.92; service unaffordability: Fisher’s exact test, *p* = 1.00; other reasons: χ^2^(1, N = 167) = 0.00, *p* = 0.98).

#### Helpline services

Successful access to helpline services (see Fig. 1, Supplementary Fig. 18) was significantly more commonly reported by adults than children (χ^2^(1, N = 2322) = 13.86, *p* < 0.001) and by female than male participants (χ^2^(1, N = 2218) = 20.19, *p* < 0.001). Out of the participants who recorded access, and after removing ‘unsure’ waiting time responses to assess only known waiting time reports, 15.38% (n = 8) were found to have waited for a month or longer, 7.69% (n = 4) longer than 6 months and 3.85 = 4% (n = 2) more than 12 months from referral (see Supplementary Fig. 18, Supplementary Table 2), and variability in waiting time between participants was significant (χ^2^(5) = 148.28, *p* < 0.001). We did not study age group- and gender-related variability in waiting time due to invalid χ^2^ tests of independence caused by insufficient expected counts.

Among participants who were unable to access helpline services (see Fig. 2), a significantly higher proportion of adults than of children (χ^2^(1, N = 1840) = 13.83, *p* < 0.001) and a significantly higher proportion of females than of males (χ^2^(1, N = 1751) = 6.65, *p* = 0.01) were affected.

Reasons for participants’ unsuccessful access (see Supplementary Fig. 18) were significantly varied (χ^2^(5) = 24.11, *p* < 0.001). The most frequently cited reasons were service unavailability (46.51%; n = 20) followed by service unsuitability/participant ineligibility (34.88%; n = 15), reasons not listed in the survey (25.58%; n = 11), long waitlists (16.28%; n = 7), rejection from service due to autism diagnosis (16.28%; n = 7) and service unaffordability (4.65%; n = 2). No significant relationship between any of the reasons and age group (rejection from service due to autism diagnosis: Fisher’s exact test, *p* = 1.00; service unsuitability/participant ineligibility: Fisher’s exact test, *p* = 0.54; service unavailability: Fisher’s exact test, *p* = 1.00; long waitlists: Fisher’s exact test, *p* = 0.30; service unaffordability: Fisher’s exact test, *p* = 1.00; other reasons: Fisher’s exact test, *p* = 0.08) or gender (rejection from service due to autism diagnosis: Fisher’s exact test, *p* = 1.00; service unsuitability/participant ineligibility: χ^2^(1, N = 40) = 0.03, *p* = 0.87; service unavailability: χ^2^(1, N = 40) = 0.33, *p* = 0.57; long waitlists: Fisher’s exact test, *p* = 0.68; service unaffordability: Fisher’s exact test, *p* = 1.00; other reasons: χ^2^(1, N = 43) = 0.72, *p* = 1.00) was noted.

#### Other services

We also examined participants’ access to services that were not listed in the survey (see Fig. 1, Supplementary Fig. 19), which was not significantly varied between child and adult participants (Fisher’s exact test, *p* = 0.67) nor between male and female participants (Fisher’s exact test, *p* = 0.43). Valid results from χ^2^ goodness-of-fit tests and tests of association for age group and gender on waiting time could not be obtained as the expected number of observations were not enough and a Fisher’s exact test was inapplicable as the number of categories for waiting time was more than two. A third (33.34%; n = 2) of participants who received these services and with known waiting times (i.e. ‘unsure’ waiting time responses excluded) had waited for 1 month or more to access them, 16.67% (n = 1) more than 6 months and 16.67% (n = 1) more than 12 months (see Supplementary Fig. 19, Supplementary Table 2).

Unsuccessful access was also not analysed since no participant in our sample had reported as such (see Fig. 2).

#### None of the services

Participants were also asked if they never had successful access to any service at any timepoint (see Fig. 1). Adults were significantly more likely to respond yes than children (χ^2^(1, N = 2322) = 11.97, *p* = 0.001) but no significant differences were found between males and females (χ^2^(1, N = 2218) = 1.76, *p* = 0.19).

We then studied participants who indicated that they never had any unsuccessful attempts at service access regardless of timepoint (see Fig. 2). Participants were found to not significantly differ across age group (χ^2^(1, N = 1840) = 0.02, *p* = 0.90) nor gender (χ^2^(1, N = 1751) = 1.77, *p* = 0.18).

#### Cross-country analysis

The UK, Spain, Poland, France and Germany (Supplementary Fig. 2) exhibited similar patterns of waiting time for autism diagnosis among the sample that had received a diagnosis (see Supplementary Fig. 4), with the greatest proportion of participants in each country receiving a diagnosis 1–6 months after referral, followed by 6–12 months and 12–18 months.

Overall, similar trends of accessed services persisted across the five countries with several differences (see Supplementary Fig. 21, Supplementary Fig. 23). Therapy, mental health, medical and financial services were four out of the five most accessed services for each of the respective countries. Educational services were also amongst the top five in the UK, Spain and Poland, with needs assessment services in France and employment services in Germany forming the top five.

The difference among the five countries in their respective proportion of participants who were able to receive needs assessment services was significant (χ^2^(4, N = 1819) = 16.36, *p* = 0.003; see Supplementary Fig. 23). The same result was observed for the other services—therapy (χ^2^(4, N = 1819) = 142.56, *p* < 0.001), mental health (χ^2^(4, N = 1819) = 49.04, *p* < 0.001), information/referral (χ^2^(4, N = 1819) = 44.87, *p* < 0.001), educational (χ^2^(4, N = 1819) = 175.74, *p* < 0.001), housing (χ^2^(4, N = 1819) = 57.14, *p* < 0.001), medical (χ^2^(4, N = 1819) = 23.15, *p* < 0.001), employment (χ^2^(4, N = 1819) = 55.01, *p* < 0.001), social care (χ^2^(4, N = 1819) = 30.96, *p* < 0.001), legal (χ^2^(4, N = 1819) = 11.54, *p* = 0.02), financial (χ^2^(4, N = 1819) = 61.85, *p* < 0.001) and helpline services (χ^2^(4, N = 1819) = 11.22, *p* = 0.02). There was also a significant difference for participants indicating no access ever to any service at any timepoint (χ^2^(4, N = 1819) = 11.54, *p* = 0.02). Variability for ‘other’ services not listed in the survey was not analysed using χ^2^ tests due to insufficient expected counts and a Fisher’s exact test was not performed since there were more than two categories within the country variable.

Similar observations were made across the five countries when participants were asked what services they had tried to access but were unable to (see Supplementary Fig. 22, 24), though with greater variety. Therapy services comprised the top five most inaccessible services in all five countries, especially in Spain, Poland and Germany where they were the most unattainable. Mental health (except in France), educational (except in the UK) and financial services (except in Poland) were among the five services that could not be obtained most by participants in four of the five countries.

For unsuccessful attempts to acquire services, cross-country comparisons were more mixed (see Supplementary Fig. 22). Autism diagnostic services did not significantly differ among the five countries in the percentage of people who could not access the services (χ^2^(4, N = 1819) = 3.75, *p* = 0.44) and neither did therapy (χ^2^(4, N = 1819) = 6.72, *p* = 0.15), educational (χ^2^(4, N = 1819) = 3.08, *p* = 0.54), housing (χ^2^(4, N = 1819) = 4.69, *p* = 0.32), medical (χ^2^(4, N = 1819) = 5.92, *p* = 0.21), employment (χ^2^(4, N = 1819) = 4.14, *p* = 0.39) and financial benefits/services (χ^2^(4, N = 1819) = 7.86, *p* = 0.10). The five countries, however, were significantly different from each other in unsuccessful access to needs assessment (χ^2^(4, N = 1819) = 15.27, *p* = 0.004), mental health (χ^2^(4, N = 1819) = 34.28, *p* < 0.001), information/referral (χ^2^(4, N = 1819) = 10.26, *p* = 0.04), social care (χ^2^(4, N = 1819) = 12.63, *p* = 0.01), legal (χ^2^(4, N = 1819) = 10.47, *p* = 0.03) and helpline services (χ^2^(4, N = 1819) = 14.91, *p* = 0.005) as well as no service at all regardless of timepoint (χ^2^(4, N = 1819) = 28.86, *p* < 0.001). No participant selected other services not listed in the survey when asked about unsuccessful access.

Spain had the highest percentage of people having had access to needs assessment (20.49%; n = 67) and financial services (38.53%; n = 126) compared to other countries, whereas Poland reported the highest rates for therapy (56.21%; n = 181), mental health (43.79%; n = 141), information/referral (24.53%; n = 79), educational (50.31%; n = 162) and housing services (17.70%; n = 57). The UK recorded the highest percentage of people accessing medical (40.31%; n = 312), social care (19.12%; n = 148), helpline (4.52%; n = 35) and other services not listed in the survey (0.26%; n = 2), and Germany as for employment (17.14%; n = 24) and legal services (7.14%; n = 10), the largest proportion of participants indicating access was in Germany.

For unsuccessful access, Poland reported the highest rate of failed attempts at accessing autism diagnostic (9.83%; n = 17), therapy (17.92%; n = 31), information/referral (11.56%; n = 20), educational (13.29%; n = 23), housing (12.72%; n = 22) and medical services (15.61%; n = 27). The UK had the highest percentage of people unsuccessful in accessing needs assessment (9.33%; n = 64), mental health (17.06%; n = 117) and helpline services (3.94%; n = 27), while France presented the highest rate of failed access for employment (7.58%; n = 15), legal (7.07%; n = 14) and financial services (15.15%; n = 30). Spain reported the greatest percentage of participants indicating unsuccessful access to social care services (12.55%; n = 31).

Across the five countries, service access was generally most inconsistent in Poland and the UK. While Poland recorded the highest proportion of participants with successful access to most services compared to other countries i.e. five—therapy, mental health, information/referral, educational and housing services—it also had the highest rate of unsuccessful attempts at access for the most services i.e. six—autism diagnostic, therapy, information/referral, educational, housing and medical services. The UK also demonstrated inconsistent trends in service access, with the highest proportion of people successfully accessing four services—medical, social care, helpline and other services—out of the five countries but also the largest percentage of people failing to obtain three services—needs assessment, mental health and helpline services.

Access to services was generally highest in Germany and Spain, compared to the other countries in the top five. Germany had the greatest rate of successful access for two services (employment and legal services), and the same was observed for Spain (needs assessment and financial services). While Germany did not have the greatest rate of failed access for any service and Spain had for one (social care services), Germany presented the highest rate of successful access to none of the services (13.57%; n = 19) and Spain reported the highest rate of unsuccessful access to none of the services (39.27%; n = 97).

On the other hand, service access was lowest in France across the five countries overall, with the largest proportion of participants indicating failed attempts to obtain three services—employment, legal and financial services—but not recording the highest rate of successful access for any service.

## Discussion

Results of the ACCESS-EU survey demonstrated the challenges in accessing services among autistic people in Europe. Nearly all services received had required most participants to wait for up to 6 months after referral, and for some services, significant age group (adult vs. child) and gender differences (male vs. female) were noted with regards to waiting time and successful as well as unsuccessful access. Reasons for unsuccessful attempts at accessing services, as informed by participants themselves, were significantly varied and differed across age group and gender in some cases.

### Autistic people’s access to services in Europe

Overall, services with the most reports of failed attempts at access were therapy, mental health, autism diagnostic, needs assessment and financial services; this is in spite of accounts of successful access to the same services. All services, however, recorded significantly varied rates of successful access among participants and of unsuccessful access alike. These findings were supported by other studies on service access among autistic people [4, 15, 21, 22], though rates of successful and unsuccessful access vary across literature perhaps because of differences in participant demographics, sample size, measures analysed and period of survey dissemination. For example, while an ASDEU study on service access reported that almost a fifth (18.9%) of adult EU participants had failed to obtain financial services within 2 years prior to their survey [21], the figure was about half of that (9.62%) in ACCESS-EU. Possible explanations include the difference in the age group of participants in the two studies—ASDEU participants were all adults while the ACCESS-EU sample was made up of both adults and carers reporting on both adults and children—and the period of circulation of the surveys—the ASDEU survey was distributed in 2017 before the COVID-19 pandemic while the ACCESS-EU survey was disseminated throughout the outbreak in 2020–2022, when most in-person services were unavailable or inaccessible [22] and so the financial pressure to fund them was reduced.

There was also significant variation in the reasons reported by participants for their failure to obtain services. The most common reasons across all services were that the service was not available or did not exist (23.08%), the service was unsuitable or not eligible for participants to use (20.04%) and other reasons not listed in the survey (18.42%). Other reasons were also reported by participants, including long waitlists (17.42%), unaffordability of service (11.80%) and being turned away by service providers because of their autism diagnosis (9.87%). Service unavailability was most frequently blamed for lack of access to needs assessment (43.79%), therapy (42.57%), housing (43.12%), employment (44.00%), social care (38.24%), legal (50.79%) and helpline services (46.51%), whereas lengthy waitlists were most attributed for unsuccessful attempts to acquire autism diagnostic (55.96%) and medical services (34.33%). Unsuitability of services or ineligibility of participants for the services was quoted by most participants as reason for lack of access to mental health (36.53%), information/referral (41.59%) and educational services (40.27%), while for financial services (40.11%), reasons other than those specified by the survey were indicated. The reasons cited by participants for their inability to access services corroborate earlier work on barriers to service access among autistic people (see Discussion—Challenges to service access).

### Association with age group

A significantly larger percentage of child compared to adult participants had ever received needs assessment, therapy, information/referral, educational and financial benefits/services, suggesting that these services are more common among autistic children currently than in the past. This is perhaps caused by a substantial improvement in service availability, perception of autism, and awareness of existing services compared to decades ago [23]. Meanwhile, adults were significantly more likely than children to indicate access to mental health, medical, employment, social care, legal and helpline services, potentially because these are all services that cater mainly to adults. For housing and other services not specified in the survey, no significant age group differences were observed, implying that adults and families with autistic children make use of them equally.

There was a significantly higher likelihood for children to encounter barriers to accessing autism diagnostic, therapy and educational services than adults, which could be explained by the fact that these services tend to be utilised more by children than by adults, so rejections are more common among children. Conversely, adults were more inclined than children to have failed in acquiring helpline services, which was expected considering that adults primarily use these services relative to children. Unsuccessful attempts to access needs assessment, mental health, information/referral, housing, medical, social care, legal and financial benefits/services did not show a significant relationship with age group; this suggests that both autistic adults and children are equally affected by difficulties accessing these services.

We then examined whether reasons cited by participants for their inability to access services were significantly related to their age group. No significant differences were observed in most cases, but notably, children were significantly more likely than adults to have cited long waitlists for unsuccessful access to needs assessment, mental health, information/referral, educational, medical and financial services, and unsuitability of service/participant ineligibility for unsuccessful access to needs assessment and medical services. This observation is supported by findings reporting lengthy wait times that autistic children and their carers face for services [24, 25] possibly exacerbated by increasing numbers of children being diagnosed with autism over the recent years [1, 3] and limited funding for children’s services [26]. Meanwhile, more adults than children attributed failure in securing diagnostic, needs assessment, mental health, information/referral and medical services to reasons not listed in the survey, therapy services to denial of service due to autism diagnosis, and mental health services to unavailability of service. Indeed, difficulty in acquiring necessary services including appropriate autism diagnostic [27] and mental health services [28] has been previously reported among autistic adults.

### Association with gender

Due to the small sample size of participants indicating other as their gender (4.44%), only male (62.2%) and female (33.3%) participants could be compared in statistical tests on gender differences. Male participants were more likely to have acquired information/referral, educational and financial benefits/services than female participants. This might have been influenced by the finding that access to these services was more prevalent among children compared to adults (see Results—Successful and unsuccessful access to services) and the overrepresentation of boys within the child sample (see Results—Demographic background). Comparatively, a significantly greater proportion of female participants than male participants had accessed mental health, medical, employment and helpline services. There was, however, no significant relationship between gender and access to needs assessment, therapy, housing, social care and legal services as well as other services not listed in the survey, suggesting no gender disparity in the accessibility of these services.

Unsuccessful attempts to obtain services were reported by a significantly higher percentage of female participants than male participants for needs assessment, therapy, mental health, information/referral, financial and helpline services. No other gender-related variations were seen for diagnostic, educational, housing, medical, employment, social care and legal services, suggesting that autistic males and females are similarly affected by problems accessing these services.

There was also a significantly higher likelihood of male participants than female participants failing to access needs assessment services because of lengthy waitlists and service unaffordability. On the other hand, female participants were significantly more likely than male participants to have reported unsuccessful access to diagnostic, needs assessment, therapy and mental health services due to reasons not covered by the survey, mental health services due to service unavailability, information/referral services due to lengthy waitlists, housing services due to unsuitability of services or participant ineligibility, and social care services due to service providers declining participants due to their autism diagnosis.

Although autistic people of all genders face obstacles in acquiring the support they need, women have been shown by our study to be significantly more likely than men to fail in their attempts to access multiple services while no such bias against men was seen in any of the services examined. Altogether, our gender-related findings on service access complement results from the literature documenting the struggles that autistic women endure in finding services that would meet their needs as both autistic people and women [29].

### Waiting time for accessed services

Only 31.24% of self-diagnosed participants had managed to access diagnostic services (which might also include services that do not directly administer diagnoses) within 6 months, and only 47.91% acquired diagnostic services within 12 months (see Supplementary Table 2). For most of the other services assessed in this survey, and for access within 2 years before survey completion, the majority of the main ACCESS-EU sample of formally diagnosed participants (≥ 57.61%) had waited up to 6 months for access upon referral. Services that were obtained by most participants within a month are those that provide time-sensitive support i.e. mental health—crisis support (60.84% of participants), mental health—inpatient (58.48%), medical—GP (82.52%), medical—emergency telephone (94.54%), medical—A&E (97.76%), medical—other services (72.00%), social care—peer support (50.00%), legal (74.32%), helpline (84.62%), and other services (66.66%). On the contrary, all the other services, which typically involve more bureaucratic processing, were slowest to reach participants, recording the lowest rate of access over time among all services. Only 57.61% of participants obtained housing services within the first 6 months, for example, and 68.48% within 12 months, while for financial benefits/services the numbers were 59.03% and 75.86% respectively. All types of social care services also took relatively long; respite care services, for instance, were accessed by 59.19% of respondents within 6 months and 79.60% within 12 months. This might be because respite care is provided mainly by family members, hence external providers are not sought, in addition to the barriers that carers encounter in attempting to utilise respite care services, such as lack of information on available services, inflexibility of services, absence of variety in services, location of services, cost of services and waiting time for services [30].

In our data overall we found longer waiting times than the ASDEU study on autistic people’s access to services in the EU which noted that, more often than not, participants waited for up to 1 month for financial services, up to 3 months for residential, employment and social support services, and up to 6 months for adult education services [21]. On the other hand, most participants in our survey took up to 6 months to access these services. This incongruence in results could be attributed to, as with the discrepancy in service access results between the two studies (see Discussion—Autistic People’s Access to Services in Europe), the difference in the period of time participants in the two studies were surveyed. While the ACCESS-EU survey was disseminated in 2020–2022, the ASDEU survey was available for 10.5 months in 2017. Waiting time for the services assessed could have worsened for reasons such as the shutdown of services in many countries during the COVID-19 pandemic [31], a shift in resources to urgent COVID-19 related healthcare services or changes in policy or funding structure that significantly reduced availability of certain services as were in England [31, 32].

It is also still possible that services with the shortest amount of waiting time that could be indicated in the survey were actually late in reaching participants, if services were urgent in nature. Given the minimum waiting time that could be indicated in the survey was ‘less than 1 month’, people could still have been left without urgent services such as inpatient psychiatric services and emergency hospital services for several days or weeks, which might be too late in some cases.

Overall, our data on waiting time for services illustrates a prolonged wait for fundamental services endured by autistic people, and both adults and children as well as men and women are in general affected. Immediate actions by the government and service providers are needed to shorten the amount of time autistic people have to wait for service access (see Discussion—Policy Recommendations below). Lengthy wait times should be avoided as they have been shown to potentially bring about detrimental repercussions to the wellbeing of autistic people [33–35].

### Implications of service access data

Data on successful and unsuccessful access to a service are not meant to be the converse of each other. A participant could have been able to access an educational service (e.g. one-to-one support) and still fail in obtaining another educational service (e.g. student wellbeing service), or they could have had opposing outcomes at acquiring the same type of service at different points in time and so report both successful and unsuccessful access of the service in the survey. Thus, relatively low rates of successful access to a particular service do not always imply relatively high rates of unsuccessful access to the service, and vice versa.

It is also important to be careful when making inferences on participants’ responses to questions on successful and unsuccessful access to services. If a service was used by more participants than another service, it could imply that the former was not needed by as many participants or was more accessible. In a similar vein, a group of participants utilising a service more often than another group might not only suggest that the former needed it more or could access it more easily, but it could also indicate that the former was more receptive to using it than the latter. Perception of services could be a barrier to access, considering that there is still stigma attached to some services e.g. mental health services [19, 20]. When participants did not report having ever received a particular service, this may indicate that the service was not needed to begin with, even if it was accessible or not associated with any stigma.

Conversely, interpretations of data on unsuccessful access to a service are more precise since the explicit implication in the survey question that *attempts* on access had been made but failed suggested that participants who answered in the affirmative did need and were generally open to attaining the service, and so any failure to secure it had primarily to do with its poor accessibility. It might also not always be the case that participants who responded in the negative had successfully received the service; instead, they might not have even sought to obtain it anyway since there was no need for it or there was a reluctance to acquire it. It would thus be more meaningful to examine data on successful and unsuccessful access together to derive more accurate conclusions on autistic people and service access. Future research could more accurately probe the accessibility of a service by asking participants if they have categorically endeavoured to utilise the service, to preclude any implication of need or receptiveness towards the service in the survey response.

Access to a service is only a part of what constitutes an autistic person’s experience when using the service. The quality of the service received is perhaps more important, as that will determine if the autistic person’s needs are met. The ACCESS-EU survey had also asked participants a range of questions on the quality of services received, and the findings are expected to be published separately.

In addition, successfully accessing a service might not always be a positive indication of autistic people’s quality of life. The use of certain services, such as inpatient psychiatric services and legal services, might suggest in some cases that existing, fundamental forms of assistance are not enough to support the community and have resulted in a spiralling down of wellbeing that necessitates urgent intervention. For instance, inadequate outpatient mental health services could have resulted in severe aggravation of an autistic person’s mental health issues that could in turn call for the use of inpatient psychiatric services. Our findings on service access could therefore also emphasise the importance of refining the quality of essential services such that autistic people may not need to turn to crisis intervention that could have been avoided. As autistic people could experience variations in wellbeing and thus in the type of services they depend on, support should be tailored to the individual so that the right form of quality support could be offered and better quality of life outcomes could be achieved.

### Cross-country comparisons

Upon reviewing data from the five most represented countries in the ACCESS-EU sample—the UK, Spain, Poland, France and Germany—service access was observed to be most inconsistent in Poland and the UK, most consistent and positive in Spain and Germany, and generally poorer outcomes were seen in France. An ASDEU study on failed attempts by autistic people at accessing services made within 2 years prior to survey completion [21] also compared the five countries and produced different findings from ACCESS-EU (see Supplementary Fig. 23, Supplementary Fig. 24), though rates of unsuccessful access were generally within the same range in both studies (0.00–22.73%). The study saw that the proportion of participants who had tried but failed to acquire housing services in the UK was 4.76% versus 5.25% as reported by ACCESS-EU, 9.02% versus 5.26% in Spain, 2.63% versus 12.72% in Poland, 11.48% versus 7.07% in France and 16.67% versus 3.36% in Germany. For employment services, the ASDEU study reported unsuccessful access in the UK to be 14.29% versus 5.39% cited in ACCESS-EU, 4.54% versus 3.64% in Spain, 5.26% versus 7.51% in Poland, 13.11% versus 7.58% in France and 16.67% versus 6.72% in Germany. Percentage of failed access to educational services in the UK according to the ASDEU study was 4.76% versus 6.56%, 4.55% versus 8.50% in Spain, 0.00% versus 13.29% in Poland, 6.58% versus 10.10% in France and 0.00% versus 10.92% in Germany. For financial services as examined in the ASDEU study, unsuccessful access was 9.52% in the UK, 22.73% in Spain, 5.26% in Poland, 21.31% in France and 16.67% in Germany, though ACCESS-EU reported them to be 8.75%, 8.10%, 11.56%, 15.15% and 10.08% in the same order. The ASDEU study also revealed that rate of failed access to social support services was 9.52% in the UK, 13.64% in Spain, 0.00% in Poland, 16.39% in France and 0.00% in Germany compared to the ACCESS-EU study which observed it to be 8.45%, 12.55%, 6.36%, 8.59%, and 4.20% accordingly. The discrepancy in results between the two studies was likely driven by the different demographic of participants that each had examined—the ASDEU sample consisted of only adults whereas ACCESS-EU participants comprised both adults and children. Different periods of survey distribution—the ASDEU study was conducted over 2017 while we implemented the ACCESS-EU survey throughout the COVID-19 outbreak in 2020–2022—might have also produced starkly varied patterns of service access, which could also be explained by different sample sizes, with the ASDEU study assessing fewer participants from the UK, Spain, Poland, France and Germany (range 6–61) compared to ACCESS-EU (range 119–686; see Supplementary Table 1). Separately, a study on epileptic care for autistic children aged 18 and below saw that the percentage of parents and carers whose autistic children were administered with electroencephalograms (EEGs) for epilepsy screening was 79% in the UK and 36% in Spain [36]. Although the study assessed children only versus ACCESS-EU which targeted participants of all age groups, and the former specifically probed EEG use in epilepsy screening in contrast to the latter investigating access to medical services as a broad category, the relative difference in results between the two countries was observed to be similar in ACCESS-EU, where the percentage of participants who had accessed medical services was higher in the UK (40.31%) than in Spain (28.75%; see Supplementary Fig. 23). Findings from these two studies nonetheless may not be contradictory since epilepsy screening via EEG might or might not constitute the medical services that ACCESS-EU participants had received.

### Challenges to service access

In the ACCESS-EU study, participants reported barriers to service access, similar to those indicated in earlier studies [4, 5], namely rejection from service due to autism diagnosis, unsuitability of service or participants’ ineligibility to use service, unavailability of service, long waitlists for service and unaffordability of service. When asked to specify, in free form text, what other barriers were faced, participants described a range of issues, the most often cited of which were exclusion by service providers. Examples of such situations include discrimination by service staff, not being deemed autistic by professionals, incapability of service staff to support autistic people (e.g. inadequate training), continuous referral from one service provider to another (e.g. from school teacher to GP to school administration to psychologist), ongoing wait for service providers to respond to participants’ applications (which forced some participants to seek private services and others to miss intervention opportunities), and participants not being in a condition to undergo service (e.g. ADHD, persistent noncompliance). A participant in our study spoke of their distress grappling with multiple life-changing issues caused directly or indirectly by the lack of access to much needed support: “I requested to be formally evaluated for autism because I was in the middle of prolonged and worsening workplace harassment… I had hoped that the diagnosis, as evidence, would contribute to my requests for urgent help… also so that the case could eventually have some expected support from the autism community… In my case, having a diagnosis does not seem to have been beneficial and only, in fact, then complicated things, by leading to successive and diverse misunderstandings or re-stigmatisations, sometimes leading to extra problems to be solved.”

It is especially critical for autistic people’s barriers to service access to be addressed sufficiently and urgently due to their complex physical [37] and psychological [38] vulnerability. As a result, autistic people require support more often and in more areas compared to non-autistic people. Mental health conditions, for instance, have been shown to co-occur more frequently in autistic people than non-autistic people [38, 39]. Furthermore, while some autistic people do utilise mainstream services, autism-specific services such as special classes [40] and career guidance [41] are notably helpful and even vital in many cases. Services catering particularly to autistic people should thus be made more available and, if possible, mainstream.

### Policy recommendations

Considering significant issues with and variations in service access for autistic people across Europe as highlighted by the ACCESS-EU survey, it is strongly recommended that targeted strategic policy and legislative measures are adopted to ensure efficient access to both mainstream and specialised services for autistic people, as necessary [42], and reduce waiting times for services so that autistic people of all ages can enjoy their rights. In particular, we highlight difficulties accessing therapy, mental health, financial, needs assessment and educational services, which were reported to be the most inaccessible by our participants (see Fig. 2), the lengthy waiting times associated with them, and the presence of barriers to accessing them, mainly service unavailability and service unsuitability (all reported in approximately one-fifth of cases).

As evidenced by the ACCESS-EU study, gaps in services provision across sectors affect autistic people of all ages. Access to social services for autistic people should hence be holistic and support autistic people at different of stages of life. When planning services provision, public authorities should consider the person’s full lifecycle so that their evolving needs are supported in different areas of life and throughout their lifetime, not least at transition times. Public authorities should therefore adopt a streamlined approach to service delivery for autistic people and coordination to enhance continuity and complementarity. It is also essential that public authorities consider the intersectionality of discrimination that autistic people may experience when accessing services and recognise the specific needs and challenges of groups at a disadvantage that require attention, and, in some cases, specialised support [43].

Coordination between international organisations will be impactful in addressing the common challenges highlighted by the results of the ACCESS-EU study, as observed over the past decade. In 2013, the Resolution 67/82 of the United Nations (UN) General Assembly (UNGA) [44] called on Member States to improve service access and social inclusivity, and the 67th World Health Assembly in 2014 adopted Resolution WHA67.8 [45] which recommended Member States to enforce or revise policies that were directed at addressing issues related to autism spectrum disorder (ASD). The European Parliament (EP) adopted a Written Declaration on autism in 2015 [46] which called on the European Commission and the Council to implement a European strategy for autism that would promote accurate diagnosis, support for all ages, research and the exchange of best practice. In 2023, the EP adopted a resolution entitled “Harmonising rights of autistic people” [47] which called on the European Commission and EU Member States to take action to realise the rights of autistic people in Europe, notably regarding service access across various areas. In the context of the European Strategy for the Rights of Persons with Disabilities 2021–2030 [48], the European Commission has committed to launching various flagship initiatives including guidance on independent living and social inclusion for persons with disabilities and a framework for enhancing service delivery and job opportunities for persons with disabilities, both to be introduced late 2024 [48].

The EU has a key role to play in supporting the initiation and coordination of policies steered towards achieving adequate services provision access for autistic people, and in ensuring that Member States adhere to common principles, policy objectives and ambitious targets and indicators in line with shared obligations under the UN Convention for the Rights of Persons with Disabilities [49] of which the EU and the UK are Parties. The EU can also support much needed investments through funding such as the European Social Fund Plus and the European Regional Development Fund to foster the development of national strategies and action plans to support adequate service provision—both mainstream and specialised—inclusive of autistic people.

Besides the supportive role that the EU can undertake, it is of course essential that national and regional public authorities, including in non-EU Member States such as the UK, adequately fund and implement targeted policies and strategies to realise the rights of their autistic communities. Current examples include the Spanish Strategy for Autism Spectrum Disorder [50] and the National Strategy for Autistic Children, Young People and Adults: 2021 to 2026 in England [51]. Availability of services and waiting times also varied across countries in the ACCESS-EU sample, so a granular approach is crucial to map actual needs and address issues disrupting autistic people’s access to services across the different countries and regions of the EU to devise targeted actions.

A strategic policy response at the EU, national and local levels requires that autistic people and their representative organisations be systematically and formally consulted and involved to identify needs and adapted solutions. It requires the active involvement of representatives from the autistic community including autistic people and their families as well as researchers with the objective of identifying the issues at national and regional levels and suggesting solutions tailored to the countries’ demographics and specific issues affecting their autistic population and wider public. Autistic people should also be meaningfully involved in all phases of planning, delivery, and monitoring of mainstream and specialised services, not least to tackle accessibility issues [43]. Services should moreover promote autistic people’s self-determination, agency, and full enjoyment of their rights. Co-production must therefore be a guiding principle and a key component of services provision.

### Limitations

There are several considerations to account for in the ACCESS-EU survey in terms of its methodology. The availability of the survey in different languages could have better represented the commonality of language speakers in the UK and EU. Czech and Slovenian are less common than other unrepresented languages but were indicated as languages people would want to see language translations for when asked in an early version of the survey. Additionally, factors that could be important in influencing service access, such as socioeconomic status, level of support needs and autism severity, were not explored to avoid lengthening the already extensive survey, but would be useful to delve into in future surveys with a more targeted scope. The response to the question asked about autism diagnosis also suggests that some might have misinterpreted the question. A small number of participants (2.11%) indicated ‘No services received’ despite having indicated previously that they had been formally diagnosed with autism. With regards to the design of the survey, it would also be more efficient if all questions on service access set the 2-year period as a precondition instead of asking participants what services they had or had not ever accessed and then when the attempts had been made. This design choice was meant to make the survey as accessible as possible but resulted in complex multileveled data. As for questions on waiting time, the option offered representing the shortest range of time was ‘less than one month’, which might not be meaningful in assessing urgent services such as mental health crisis support that need to reach clients much sooner than a month. Future studies should allow for more finite response options such as adopting an open-ended year-and-month format. In addition, across all services, a relatively high percentage of participants selected “other” (mean 18.42%) instead of the other predefined options for the question on reasons for unsuccessful access, suggesting that the predefined options might not have accurately represented the most frequently cited reasons. The options had been chosen to represent what we believed were universal barriers to access, common in all or at least most of the services examined in the survey. However, the relatively large proportion of “other” responses as well as participants’ explanations for what the “other” reasons were will be investigated further and will guide us when developing future studies. On a related note, the phrasing and format of the predefined options for the question on reasons for unsuccessful access—for instance, “service denied due to autism diagnosis” —might not fully comprise the complexity of autistic people’s difficulties when attempting service access. While it might be better to frame the question in an open-ended format, the comprehensive nature of the ACCESS-EU survey meant that a quantitative approach, where MCQs are usually adopted, is the most effective means of collecting and analysing data instead of free text. We will consider better ways of illustrating barriers to service access in future studies such as a more targeted survey on the topic using open-ended questions.

The data collected in the survey were also limited in several ways. The country representation in our sample did not fully reflect that in the EU and the UK—while some of the most populous countries in the region such as Germany and France also had the greatest number of participants in ACCESS-EU, others such as Italy (4th across the EU and the UK) and Romania (7th) were 9th and 23rd respectively in the study. In spite of reaching out to multiple autism organisations across the region, some countries remained underrepresented. This should therefore be taken into account when considering our results, which averaged across the entire sample of EU countries and the UK. The underrepresentation of certain countries is likely, in part, because the languages in which the survey was available did not reflect the ones most spoken by populations across the region, as previously mentioned. Another possible reason is that the Czech, Polish and Slovenian translations of the survey were available only after survey versions in English, French, German and Italian were launched, resulting in a slower reach to participants in the Czech Republic, Poland and Slovenia and thus their underrepresentation. Concerns about sample representation also extend to gender distribution, where only 4.44% of participants were other-identifying. Because the group was too small for meaningful statistical comparisons on gender, it was excluded from gender-related analyses. Fisher’s exact tests were carried out if both variables had two categories each but could not be performed in other cases. The data should also be studied with discretion, keeping in mind that survey responses made by carers on behalf of autistic family members might not represent actual experiences of the autistic individuals. Although carers were asked about the experience of their family member in the carer version of the survey, responses might be influenced by their own experience of supporting their family member trying to access the services. Furthermore, even when analysis only included access attempts within 2 years before the survey was completed, the results might still not accurately reflect the most current state of service access and relevant policies, especially since the survey was first launched in 2020—5 years prior to publication.

Consideration should moreover be given to how data in the survey were analysed. The translation of open-ended text responses in the survey using online tools instead of qualified human translators, which was due to the large volume of data and funding restrictions, may be less accurate, although we had verified the English translations manually and resolved unclear translations with native speakers. Nevertheless, we acknowledge that some linguistic or cultural nuances and language-specific terms in non-English languages might have been understood differently when they were translated back to English en masse using online tools. Where data was recategorised, this was checked by a second team member; however, there is still a risk that the intention of the participant was misinterpreted or influenced by subjective bias from the research team. The grouping of participants into adults and children was also based on the cutoff age of 16, with participants at this age or older providing self-report data. Depending on the age cutoff for different services, the 16–18 age range might be eligible for different types of services. There may also be variation in the countries included in the survey regarding the cutoff for an individual to legally be considered an adult. Age group-related results should hence be considered carefully with these caveats in mind. Moreover, the fact that multiple comparisons were not adjusted for, due to the approach adopted in our analyses and their exploratory nature (see Methods—Data Analysis), should be taken into account when evaluating the results.

### Conclusions

Autistic people of all ages experience prominent and numerous difficulties when attempting to access the services they depend on, including long waiting times [4, 5]. Problems and delays in accessing essential services have been shown to have serious consequences on the wellbeing of autistic people [33–35] including physical [52] and mental health problems [11], suicidal ideation [13] and reduced employment opportunities [53]. Indeed, many in this study reported challenges with accessing services, and where services could not be accessed, this was for a range of different reasons including unsuitability, unavailability and unaffordability of services as well as long waiting times and rejection from services due to autism diagnosis. It is therefore vital that policies working towards improving service access are enacted and harmonised across the region for autistic communities throughout Europe to receive the support they need.

## Supplementary Information

Additional file 1: Supplementary file 1 contains Supplementary Tables 1 and 2, and Supplementary Figs. 1–24.

## Data Availability

No datasets were generated or analysed during the current study.

## Abbreviations

ACCESS-EU: Autism Services Access - EU
AIMS-2-TRIALS: Autism Innovative Medicine Studies-2-Trials
A-Reps: Autism representatives
ASD: Autism spectrum disorder
ASDEU: Autism Spectrum Disorder in the European Union
CARD: Cambridge Autism Research Database
EEG: Electroencephalogram
EP: European Parliament
EU: European Union
UK: United Kingdom
UN: United Nations
UNGA: United Nations General Assembly
