# Supplementary material for: Access to services for autistic people across Europe
Source: Mol Autism. 2025 Jun 14;16:35. doi: 10.1186/s13229-025-00664-2 (PMC12166561; doi:10.1186/s13229-025-00664-2)
Supplement: Supplementary file 1 — Additional file 1: Supplementary file 1 contains Supplementary Tables 1 and 2, and Supplementary Figs. 1–24. [file 13229_2025_664_MOESM1_ESM.pdf]

Supplementary Table 1

Distribution of Countries of Participants

Country	Successful access		Unsuccessful access	
	Frequency	Percent (%)	Frequency	Percent (%)
Austria	9	0.39	7	0.38
Belgium	40	1.72	31	1.68
Croatia	3	0.13	3	0.16
Cyprus	6	0.26	5	0.27
Czech Republic	95	4.09	79	4.29
Denmark	3	0.13	3	0.16
Estonia	6	0.26	5	0.27
Finland	35	1.51	25	1.36
France	257	11.07	198	10.76
Germany	140	6.03	119	6.47
Greece	1	0.04	1	0.05
Hungary	4	0.17	4	0.22
Ireland	22	0.95	19	1.03
Italy	63	2.71	55	2.99
Latvia	1	0.04	1	0.05
Lithuania	1	0.04	1	0.05
Luxembourg	74	3.19	63	3.42
Malta	13	0.56	9	0.49
Netherlands	29	1.25	22	1.20
Poland	322	13.87	173	9.40
Portugal	2	0.09	2	0.11
Romania	2	0.09	2	0.11
Slovakia	2	0.09	2	0.11

Country	Successful access		Unsuccessful access	
	Frequency	Percent (%)	Frequency	Percent (%)
Slovenia	86	3.70	73	3.97
Spain	326	14.04	247	13.42
Sweden	6	0.26	5	0.27
United Kingdom	774	33.33	686	37.28
**Total**	**2,322**	**100.00**	**1,840**	**100.00**

Supplementary Table 2

Waiting time for services accessed less than two years before survey completion

Service		Percentage of participants for each range of waiting time (%)							
		< 1 month	1-6 months	6-12 months	12-18 months	18 months - 3 years	> 3 years	Unsure	No service received
Autism diagnosis		6.39	29.84	17.74	13.48	9.78	7.75	4.44	2.20
Autism diagnostic (for self-diagnosed)		6.19	20.35	14.16	14.16	14.16	15.93	4.42	10.62
Needs assessment		20.21	40.43	15.25	6.74	5.67	4.26	7.45	
	Early intervention	25.00	53.47	13.19	1.39	1.39	4.17	1.39	
	Speech and language	32.59	39.26	10.74	6.30	2.22	5.19	3.70	
	Occupational therapy	29.85	37.81	14.93	8.96	1.99	1.49	4.98	
Therapy	Physiotherapy	43.51	39.69	5.34	3.05	2.29	2.29	3.82	
	Behavioural support/ therapy	27.30	41.98	13.99	7.85	1.71	3.75	3.41	NA
	Dietitian/ nutritionist	37.68	31.88	14.49	2.90	4.35	4.35	4.35	
	Other	33.54	35.98	8.54	7.93	5.49	4.27	4.27	
	Crisis support	55.80	23.76	4.97	3.87	1.66	1.66	8.29	
Mental health	Outpatient	27.44	38.46	14.10	7.69	3.08	2.82	6.41	
	Inpatient	53.91	26.96	6.09	4.35	0.00	0.87	7.83	
	Other	30.34	37.08	11.24	6.74	3.37	4.49	6.74	
Information/referral		32.78	40.66	8.71	5.81	1.24	2.90	7.88	

Service		Percentage of participants for each range of waiting time (%)							
		< 1 month	1-6 months	6-12 months	12-18 months	18 months - 3 years	> 3 years	Unsure	No service received
Educational		34.35	32.24	12.85	6.54	3.74	5.14	5.14	
Housing		18.81	33.66	9.90	11.88	3.96	12.87	8.91	
Medical	GP	79.76	12.38	2.14	0.95	0.00	1.43	3.33	
	Specialist	24.62	47.69	12.69	7.31	3.08	1.15	3.46	
	Dental	48.36	36.98	8.32	1.97	0.66	1.09	2.63	
	Emergency telephone	92.86	3.57	1.79	0.00	0.00	0.00	1.79	
	A&E	96.67	0.00	0.00	0.00	1.11	1.11	1.11	
	Other	69.23	15.38	7.69	3.85	0.00	0.00	3.85	
Employment		37.29	38.14	8.47	5.93	0.85	2.54	6.78	
Social care	Respite care	23.53	33.33	19.61	7.84	3.92	7.84	3.92	
	Peer support	45.45	30.30	9.09	3.03	3.03	0.00	9.09	
	Support group	46.15	38.46	5.77	3.85	1.92	1.92	1.92	
	Support with daily living skills	24.29	37.14	10.00	8.57	7.14	7.14	5.71	
	Advocacy	39.47	28.95	7.89	2.63	7.89	5.26	7.89	
	Other	25.26	35.79	14.74	3.16	4.21	8.42	8.42	
Legal		72.37	15.79	2.63	2.63	2.63	1.32	2.63	
Financial		17.75	36.63	15.51	11.91	4.94	5.39	7.87	
Helpline		75.86	6.90	3.45	0.00	1.72	1.72	10.34	
Other		66.67	16.67	0.00	0.00	16.67	0.00	0.00	

Supplementary Figure 1

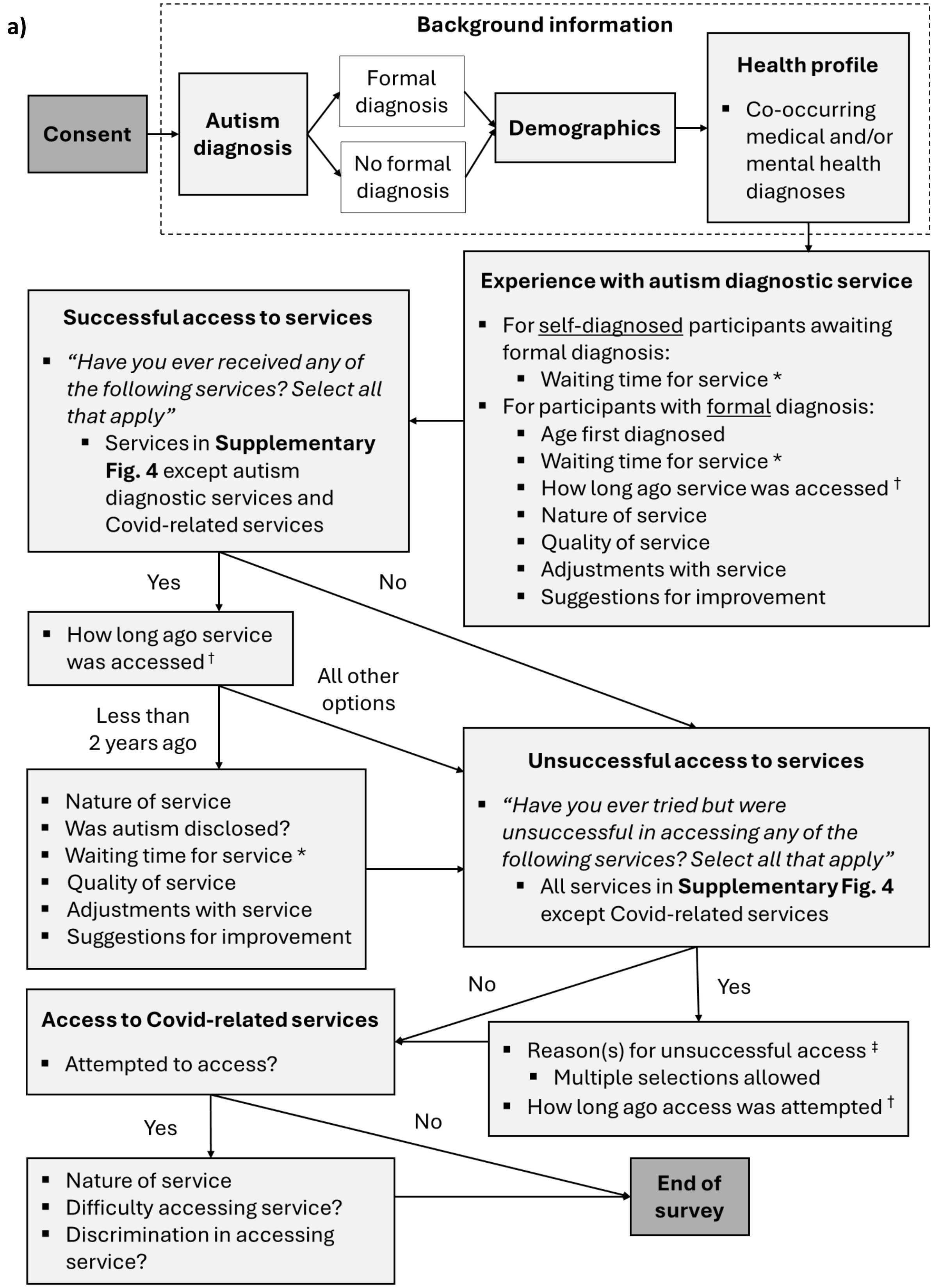

b)

* Waiting time:

- Less than 1 month
- 1-6 months
- Greater than 6 months but less than 12 months
- 12-18 months
- Greater than 18 months but less than 3 years
- 3 years and above
- Unsure
- No service received

† How long ago service was accessed/access was attempted:

- Less than 2 years ago
- 2-5 years ago
- More than 5 years ago
- Don't remember

‡ Reason(s) for unsuccessful access:

- Less than 2 years ago
- 2-5 years ago
- More than 5 years ago
- Don't remember

Supplementary Fig. 1. a) Summary of ACCESS-EU survey flow. Survey questions denoted by *, † and ‡ are multiple-choice questions with options in **b**). **b**) Options for questions on: waiting time (for an accessed service or, in the "Experience with autism diagnostic service" section of the survey (see **a**)), for autism diagnostic service yet to be received by self-diagnosed autistic participants who were awaiting formal diagnosis (questions denoted by * in **a**))); when a successfully accessed service was received or an unsuccessful attempt at access was made (questions denoted by † in **a**)); and reasons for unsuccessful access to a service (questions denoted by ‡ in **a**)).

Supplementary Figure 2

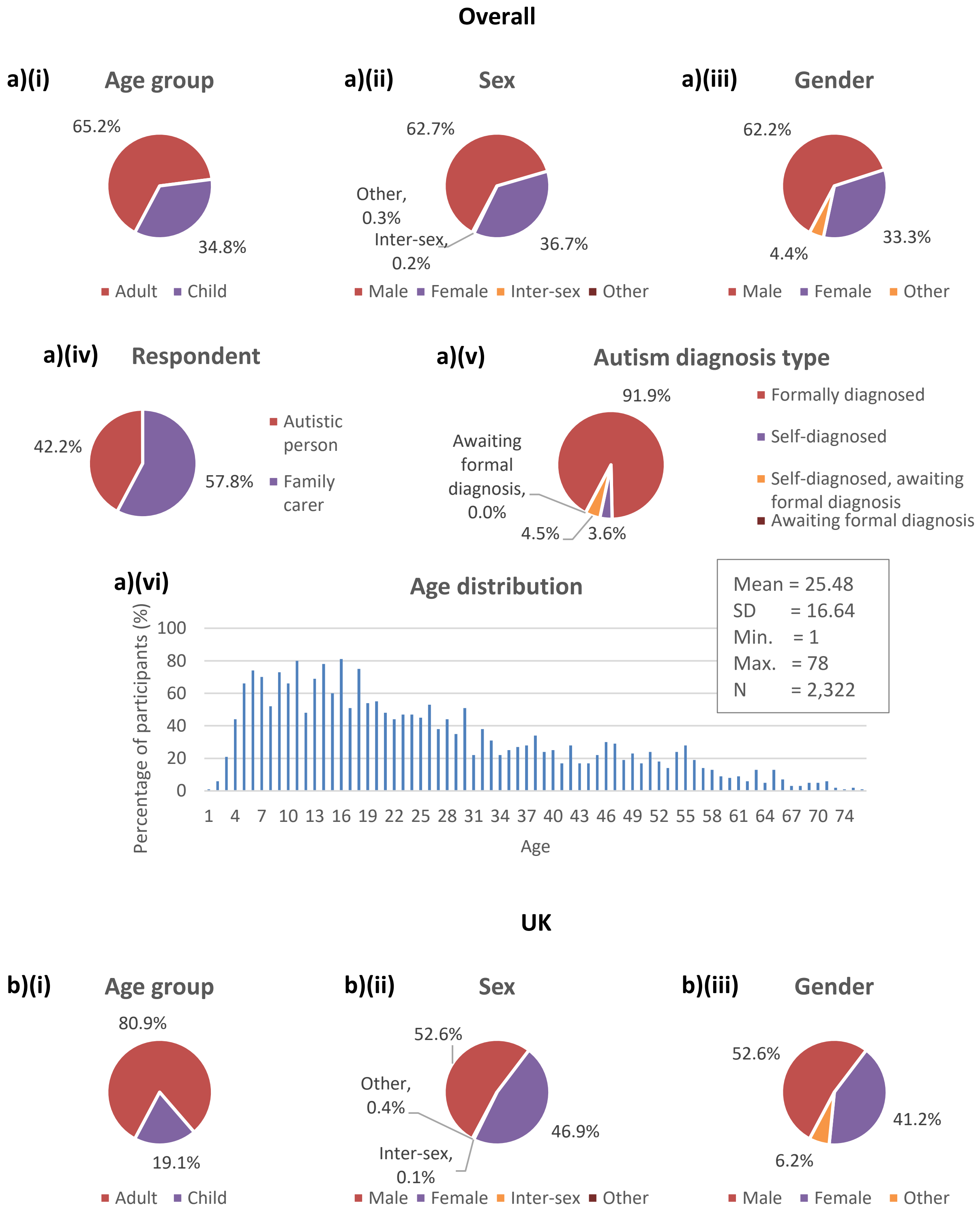

b)(iv)

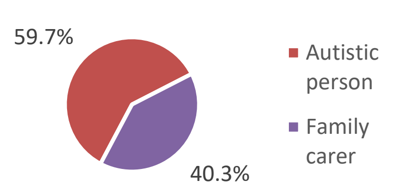

b)(v)

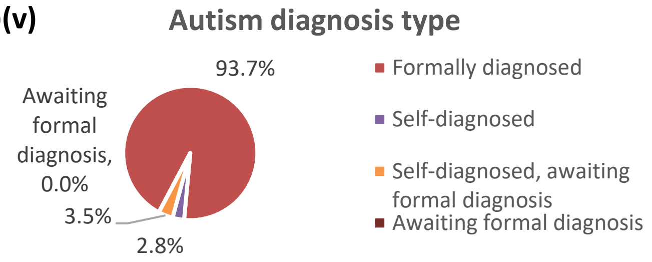

b)(vi)

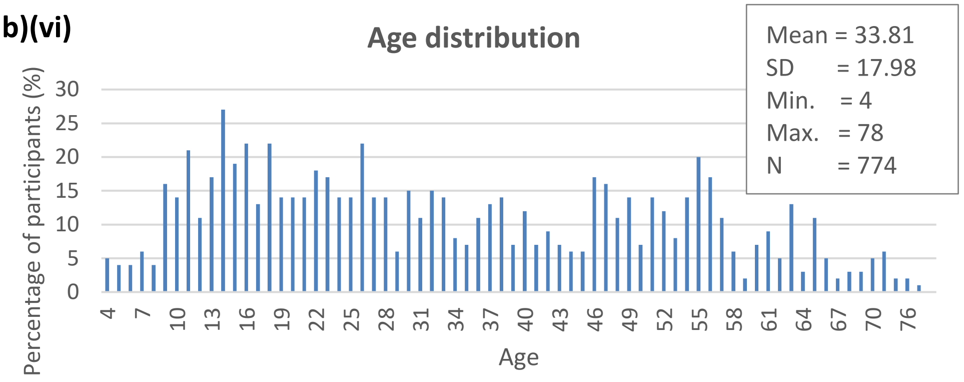

Spain

c)(i)

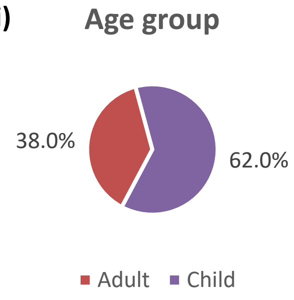

c)(ii)

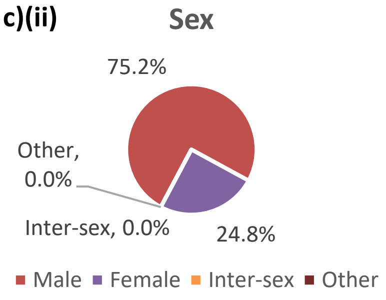

c)(iii)

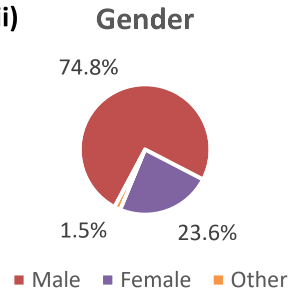

c)(iv)

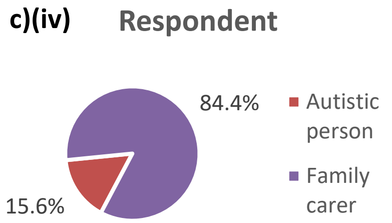

c)(v)

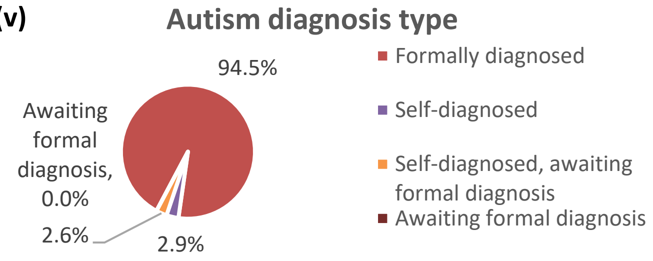

c)(vi)

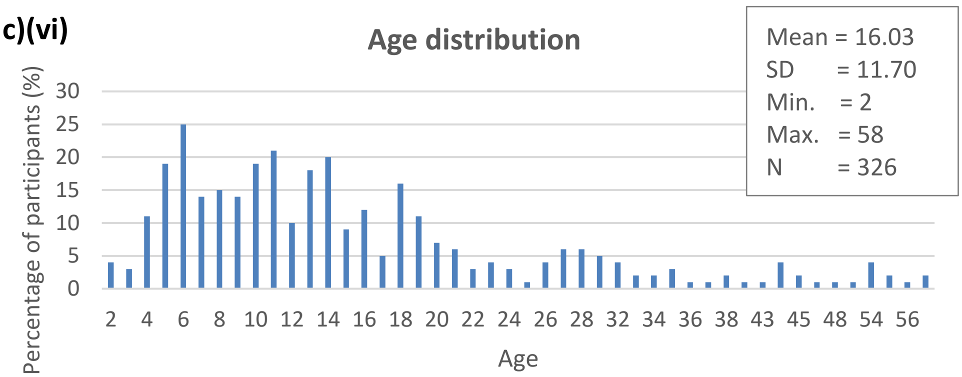

Poland

d)(i)

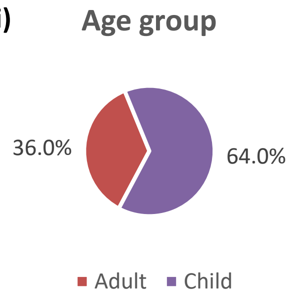

d)(ii)

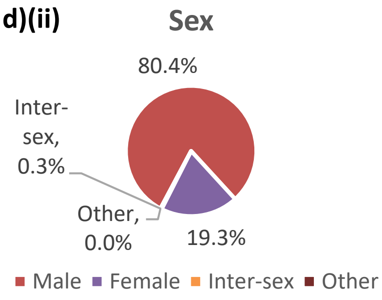

d)(iii)

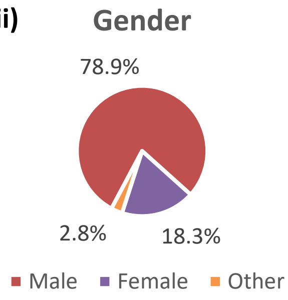

d)(iv)

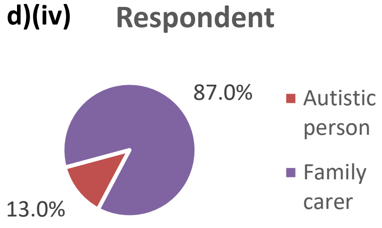

d)(v)

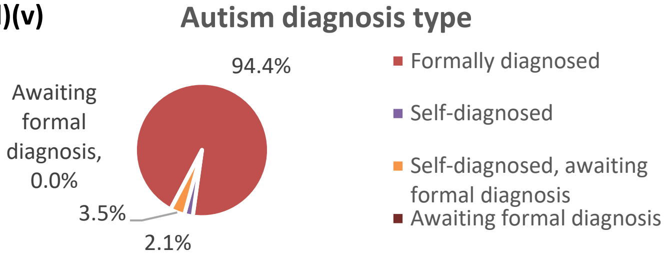

d)(vi)

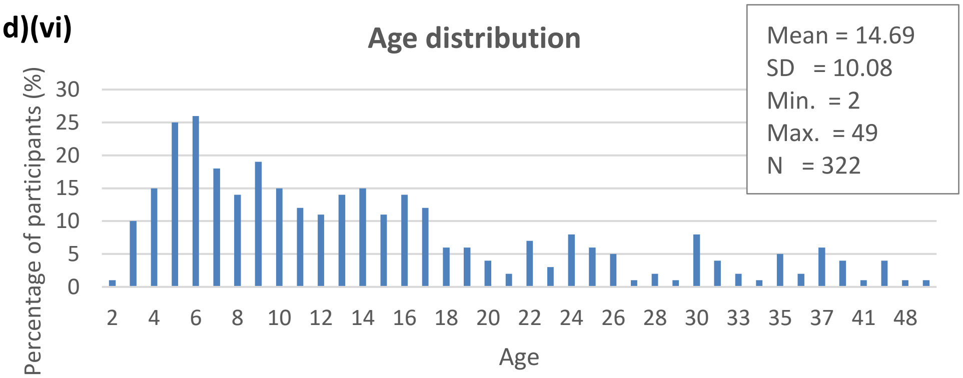

France

e)(i)

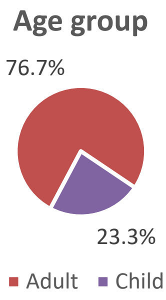

e)(ii)

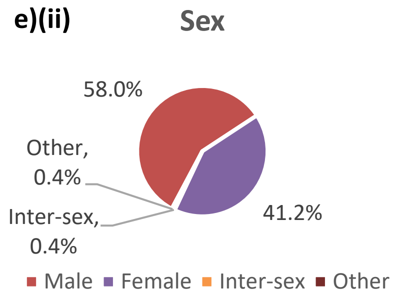

e)(iii)

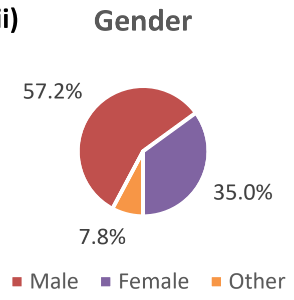

e)(iv) Respondent

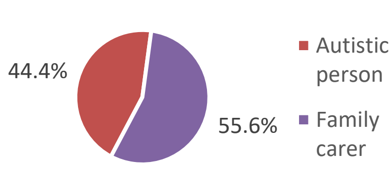

e)(v)

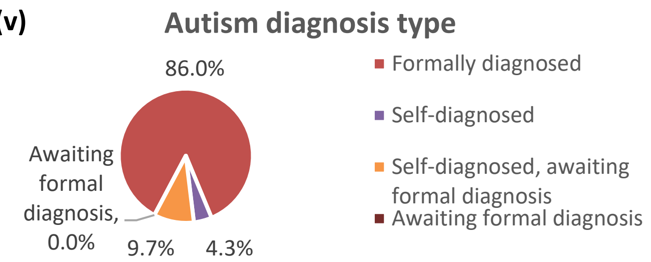

e)(vi)

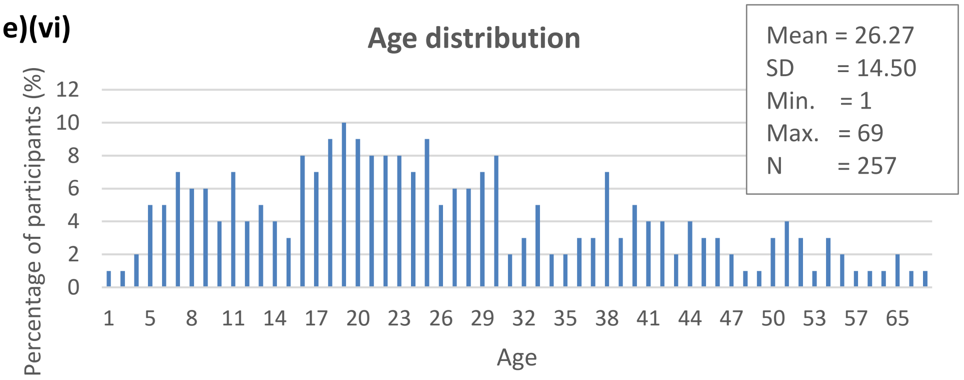

Germany

f)(i)

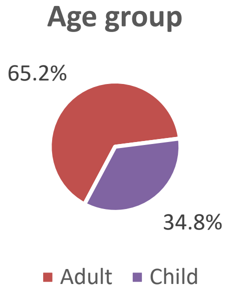

f)(ii)

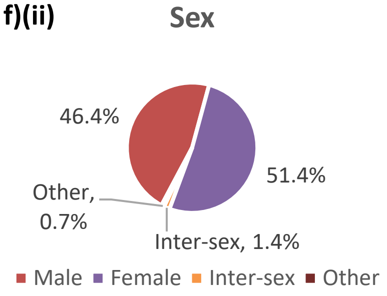

f)(iii)

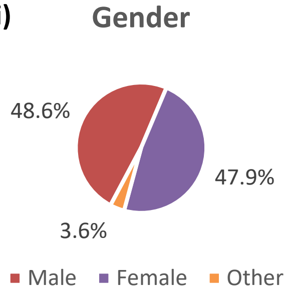

f)(iv)

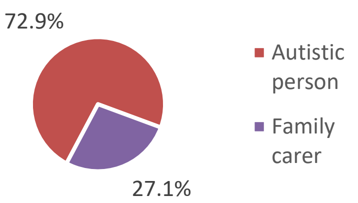

f)(v)

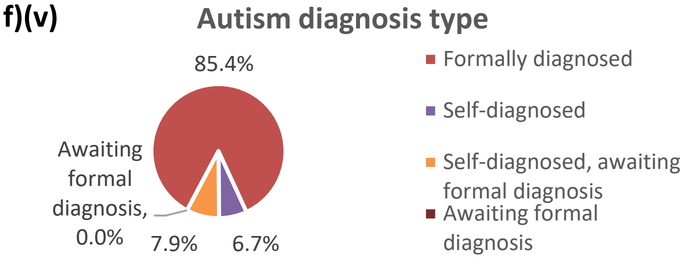

f)(vi)

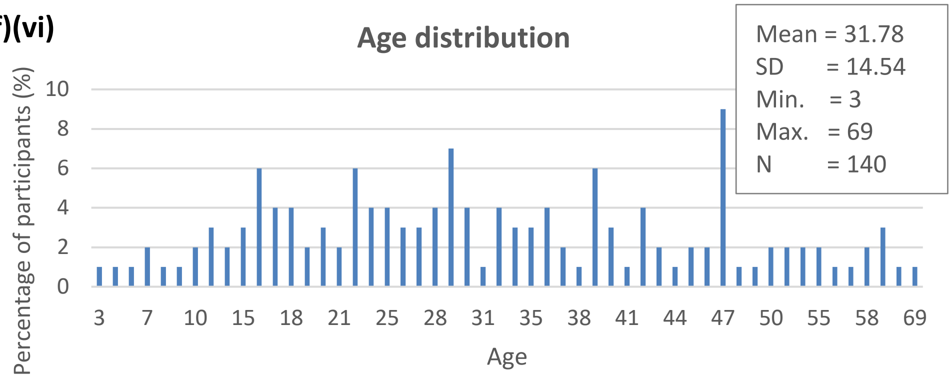

Supplementary Fig. 2. a) Participants overall. **(i)** Age group, n=2,322. **(ii)** Sex assigned at birth, n=2,322. **(iii)** Identified gender, n=2,321 (one participant wished to conceal their gender). **(iv)** Distribution of survey respondents as self-reporting autistic participants or family carers, n=2,322. **(v)** Type of autism diagnosis, n=2,322. **(vi)** Age distribution, n=2,322. **b)** Participants in the UK. **(i)** Age group, n=774. **(ii)** Sex assigned at birth, n=774. **(iii)** Identified gender, n=774. **(iv)** Distribution of survey respondents as self-reporting autistic participants or family carers, n=774. **(v)** Type of autism diagnosis, n=774. **(vi)** Age distribution, n=774. **c)** Participants in Spain. **(i)** Age group, n=326. **(ii)** Sex assigned at birth, n=326. **(iii)** Identified gender, n=326. **(iv)** Distribution of survey respondents as self-reporting autistic participants or family carers, n=326. **(v)** Type of autism diagnosis, n=326. **(vi)** Age distribution, n=326. **d)** Participants in Poland. **(i)** Age group, n=322. **(ii)** Sex of assigned at birth, n=322. **(iii)** Identified gender, n=322. **(iv)** Distribution of survey respondents as self-reporting autistic participants or family carers, n=322. **(v)** Type of autism diagnosis, n=322. **(vi)** Age distribution, n=322. **e)** Participants in France. **(i)** Age group, n=257. **(ii)** Sex of assigned at birth, n=257. **(iii)** Identified gender, n=257. **(iv)** Distribution of survey respondents as self-reporting autistic participants or family carers, n=257. **(v)** Type of autism diagnosis, n=257. **(vi)** Age distribution, n=257. **f)** Participants in Germany. **(i)** Age group, n=140. **(ii)** Sex assigned at birth, n=140. **(iii)** Identified gender, n=140. **(iv)** Distribution of survey respondents as self-reporting autistic participants or family carers, n=140. **(v)** Type of autism diagnosis, n=140. **(vi)** Age distribution, n=140.

Supplementary Figure 3

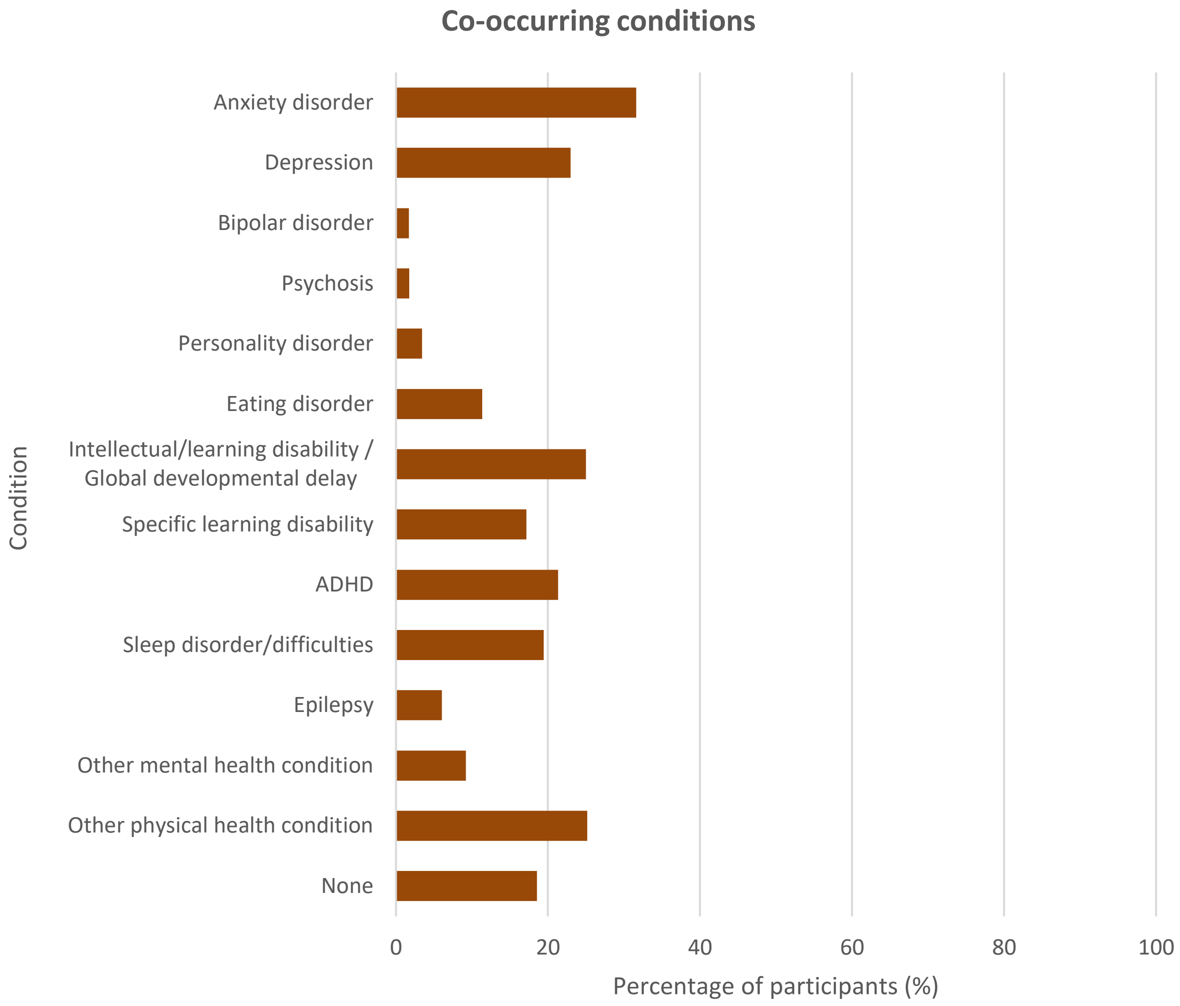

Supplementary Fig. 3. Co-occurring health conditions of participants other than autism diagnosis, n=2,322.

Supplementary Figure 4

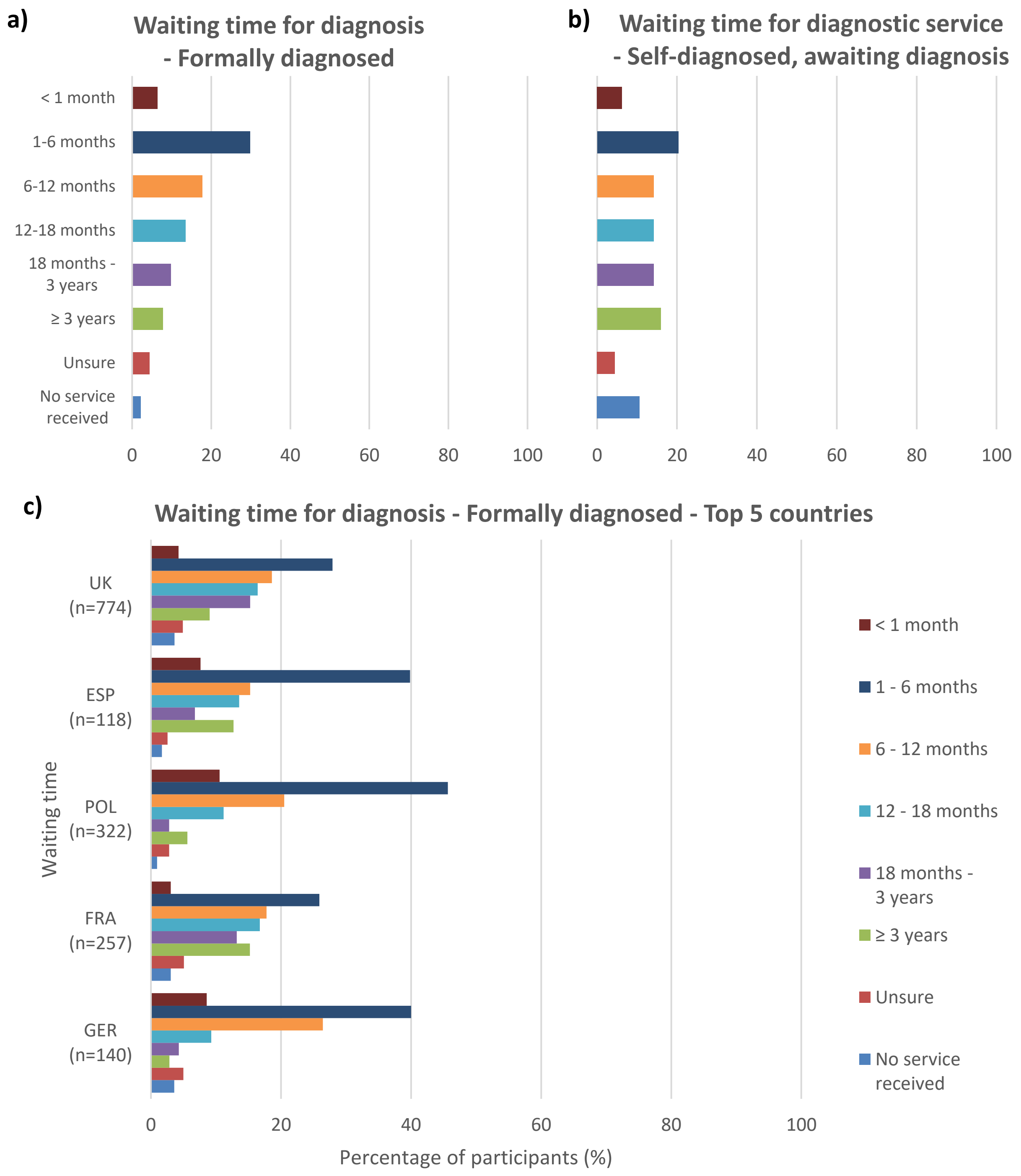

Supplementary Fig. 4. a) How long formally diagnosed participants had waited to receive their autism diagnosis, n=2,322. **b)** How long self-diagnosed participants awaiting formal diagnosis had been waiting to access a diagnostic service, n= 113. **c)** How long formally diagnosed participants in the five most represented countries in the survey i.e. the UK, Spain, Poland, France and Germany had waited to receive their autism diagnosis, n=1,819.

Supplementary Figure 5

Autism Diagnostic Service

- Services associated with autism diagnosis
- E.g. assessment, follow-up review, post-diagnosis information, referral

Needs Assessment

- An overall assessment of your family member's support and service needs

Therapy Service

- Allied health services
- Subcategories: 1) early intervention, 2) occupational therapy, 3) speech and language therapy, 4) behaviour support/therapy, 5) physiotherapy, 6) nutritionist/dietitian service, 7) other therapy service

Mental Health Service

- Subcategories: 1) mental health crisis support, 2) outpatient mental health support, 3) inpatient psychiatric treatment, 4) other mental health service

Information/Referral Service

- Service providing information on local services
- E.g. training, education, referral centre

Educational Service

- E.g. specialist support or specialist educational setting within pre-school, nursery school, primary, secondary, tertiary settings including technical colleges and universities

Housing Service/Assistance

- Assistance with securing and/or maintaining accommodation
- E.g. semi-independent supported living, residential support living, subsidised housing

Medical Service

- Subcategories: 1) general practitioner, 2) dental service, 3) specialist medical service, 4) emergency telephone service, 5) accident and emergency service, 6) other medical service

Employment Service

- E.g. vocational training, recruitment agency, careers advice

Social Care Service

- Subcategories: 1) support group, 2) peer support, 3) support with daily living skills, 4) advocacy service, 5) respite care for carers, 6) other social care service

Legal Service

- Seeking legal advice or instructing a solicitor to act on the autistic person's behalf

Financial Benefits/Assistance

- Government assisted benefits/schemes

Helpline Service

- Information hotlines
- E.g. on local autism services, health and mental health information

Supplementary Fig. 5. Definition of services examined in the ACCESS-EU survey. Therapy, mental health, medical and social care services were broken down into subcategories in follow-up questions on waiting time for service accessed as well as nature and quality of service accessed. Participants were able to see the definitions on screen during the survey when asked if they had ever accessed the services or attempted to access them but failed.

Supplementary Figure 6

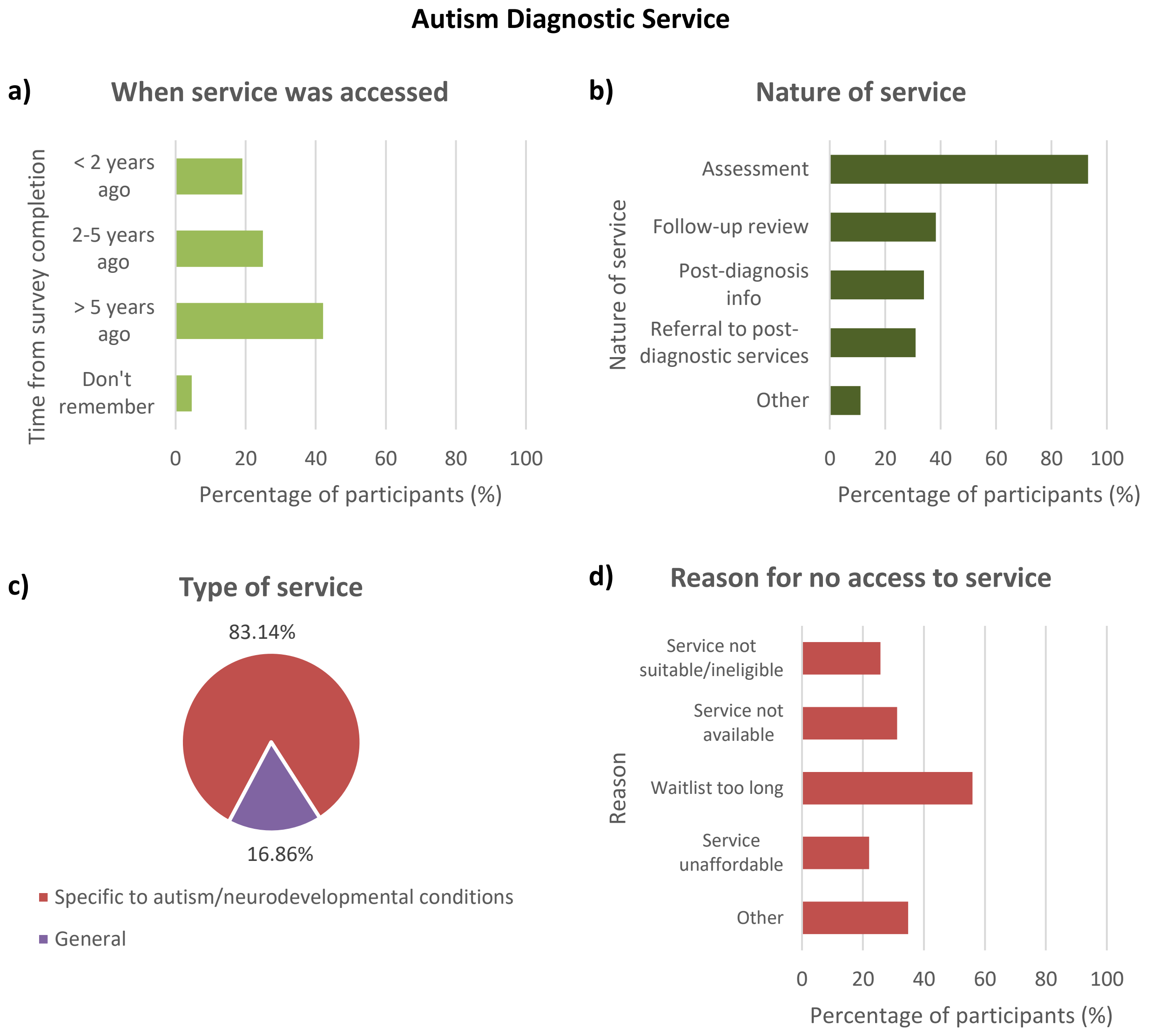

Supplementary Fig. 6. Autism diagnostic service. **a)** When service was last accessed from time of survey completion. Multiple responses allowed if service was accessed more than once, n=2,105. **b)** Nature of service accessed less than two years before survey completion. Multiple responses allowed if service was accessed more than once, n=442. **c)** Type of service accessed less than two years before survey completion. Only one response allowed, n=439. **d)** Reason for failed attempt at accessing service made less than two years before survey completion, according to participants. Multiple responses allowed, n=109.

Supplementary Figure 7

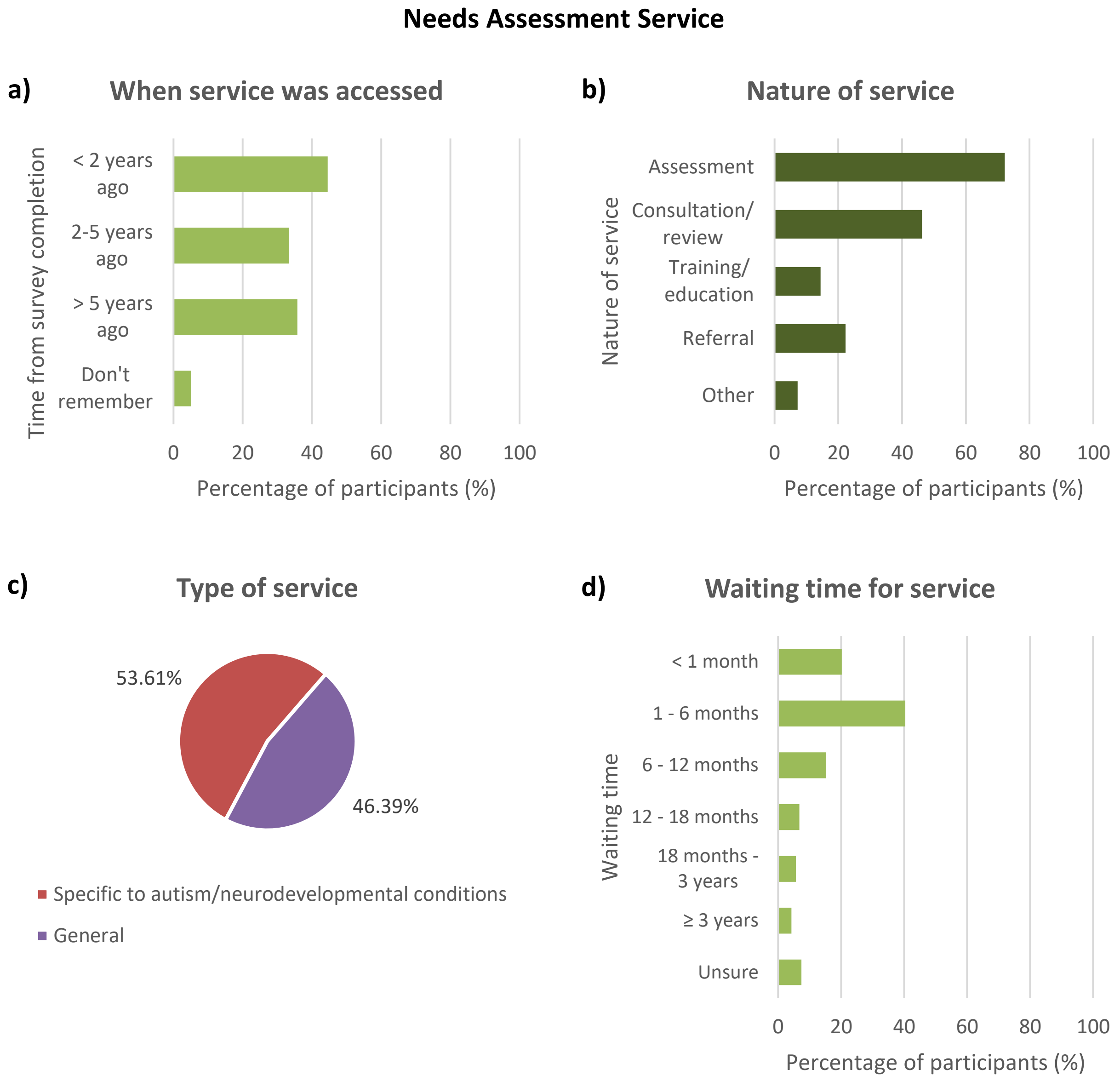

e)

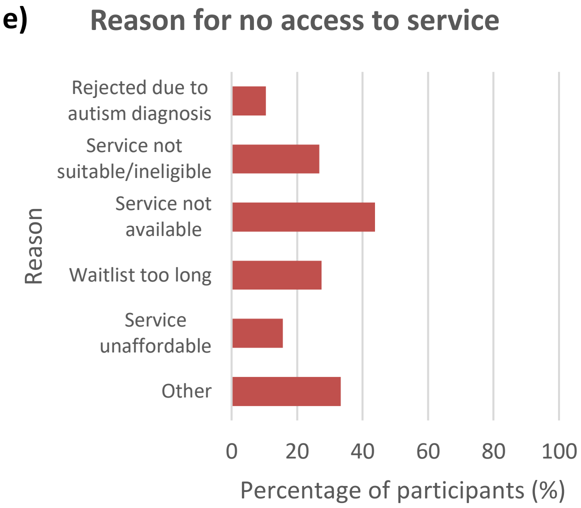

Supplementary Fig. 7. Needs assessment service. **a)** When service was last accessed from time of survey completion. Multiple responses allowed if service was accessed more than once, n=779. **b)** Nature of service accessed less than two years before survey completion. Multiple responses allowed if service was accessed more than once, n=346. **c)** Type of service accessed less than two years before survey completion. Only one response allowed, n=291. **d)** Waiting time upon referral for service accessed less than two years before survey completion. Only one response allowed, n=282. **e)** Reason for failed attempt at accessing service made less than two years before survey completion, according to participants. Multiple responses allowed, n=153.

Supplementary Figure 8

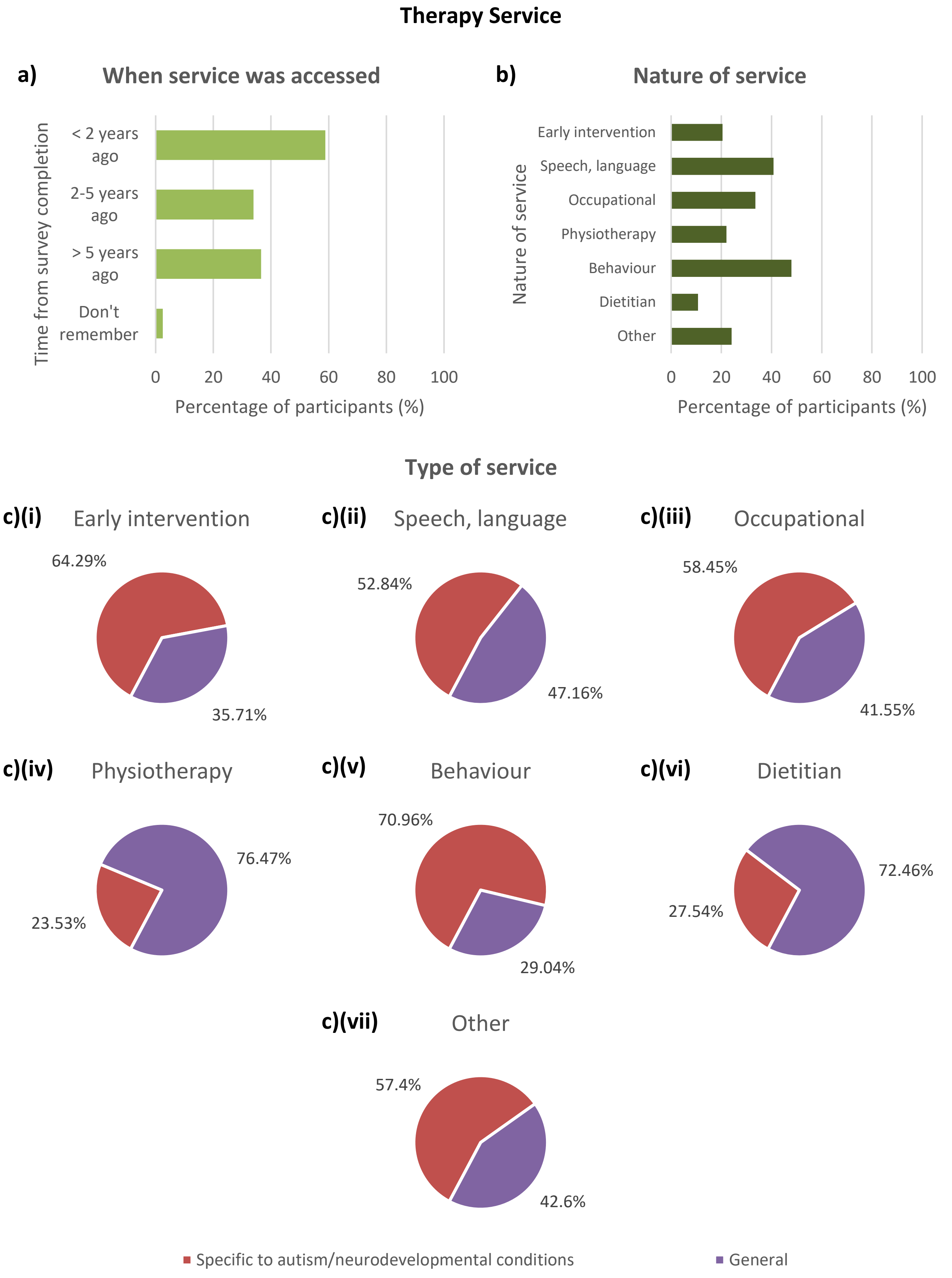

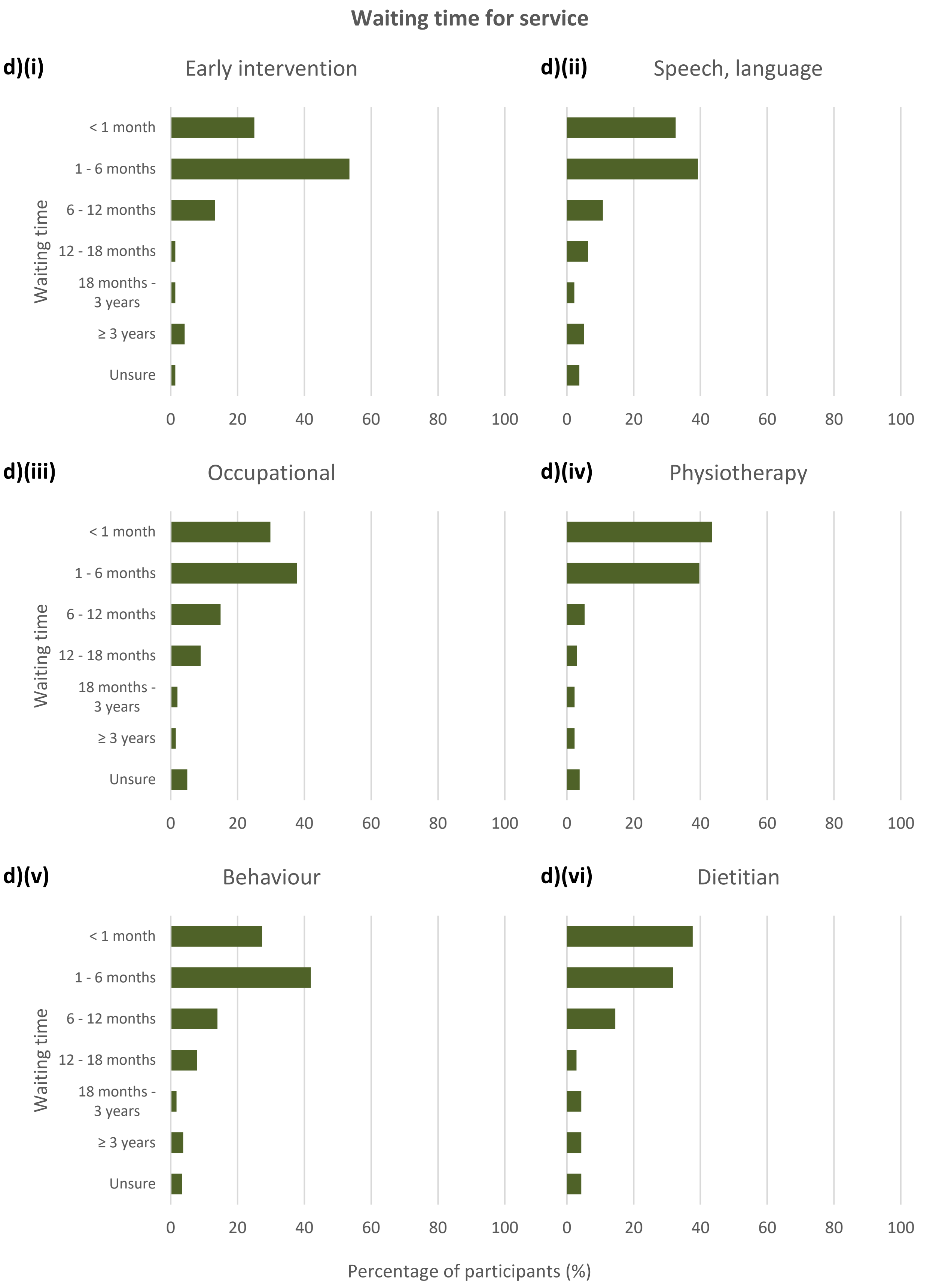
Waiting time for service
d)(i)
Early intervention
d)(ii)
Speech, language
d)(iii)
Occupational
d)(iv)
Physiotherapy
d)(v)
Behaviour
d)(vi)
Dietitian
Waiting time
< 1 month
1 - 6 months
6 - 12 months
12 - 18 months
18 months - 3 years
≥ 3 years
Unsure
0
20
40
60
80
100
Percentage of participants (%)

d)(vii)

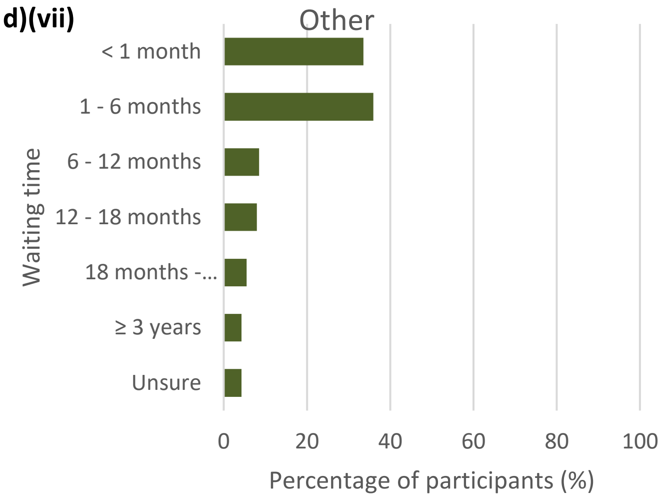

e)

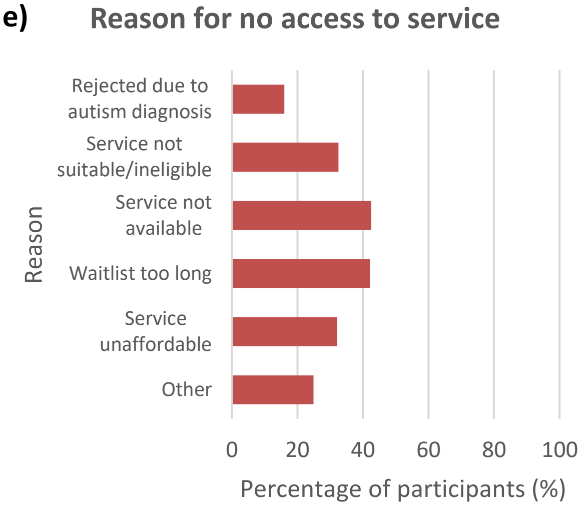

Supplementary Fig. 8. Therapy service. **a)** When service was last accessed from time of survey completion. Multiple responses allowed if service was accessed more than once, n=1,318. **b)** Nature of service accessed less than two years before survey completion. Multiple responses allowed if service was accessed more than once, n=781. **c)** Type of service accessed less than two years before survey completion for **(i)** early intervention service, n=154, **(ii)** speech and language therapy service, n=282, **(iii)** occupational therapy service, n=207, **(iv)** physiotherapy service, n=136, **(v)** behavioural support/therapy service, n=303, **(vi)** dietitian/nutritionist service, n=69 and **(vii)** other therapy service, n=169. Only one response allowed. **d)** Waiting time upon referral for service accessed less than two years before survey completion for **(i)** early intervention service, n=144, **(ii)** speech and

language therapy service, n=270, **(iii)** occupational therapy service, n=201, **(iv)** physiotherapy service, n=131, **(v)** behavioural support/therapy, n=293, **(vi)** dietitian/nutritionist service, n=69 and **(vii)** other therapy service, n=164. Only one response allowed. **e)** Reason for failed attempt at accessing service made less than two years before survey completion, according to participants. Multiple responses allowed, n=249.

Supplementary Figure 9

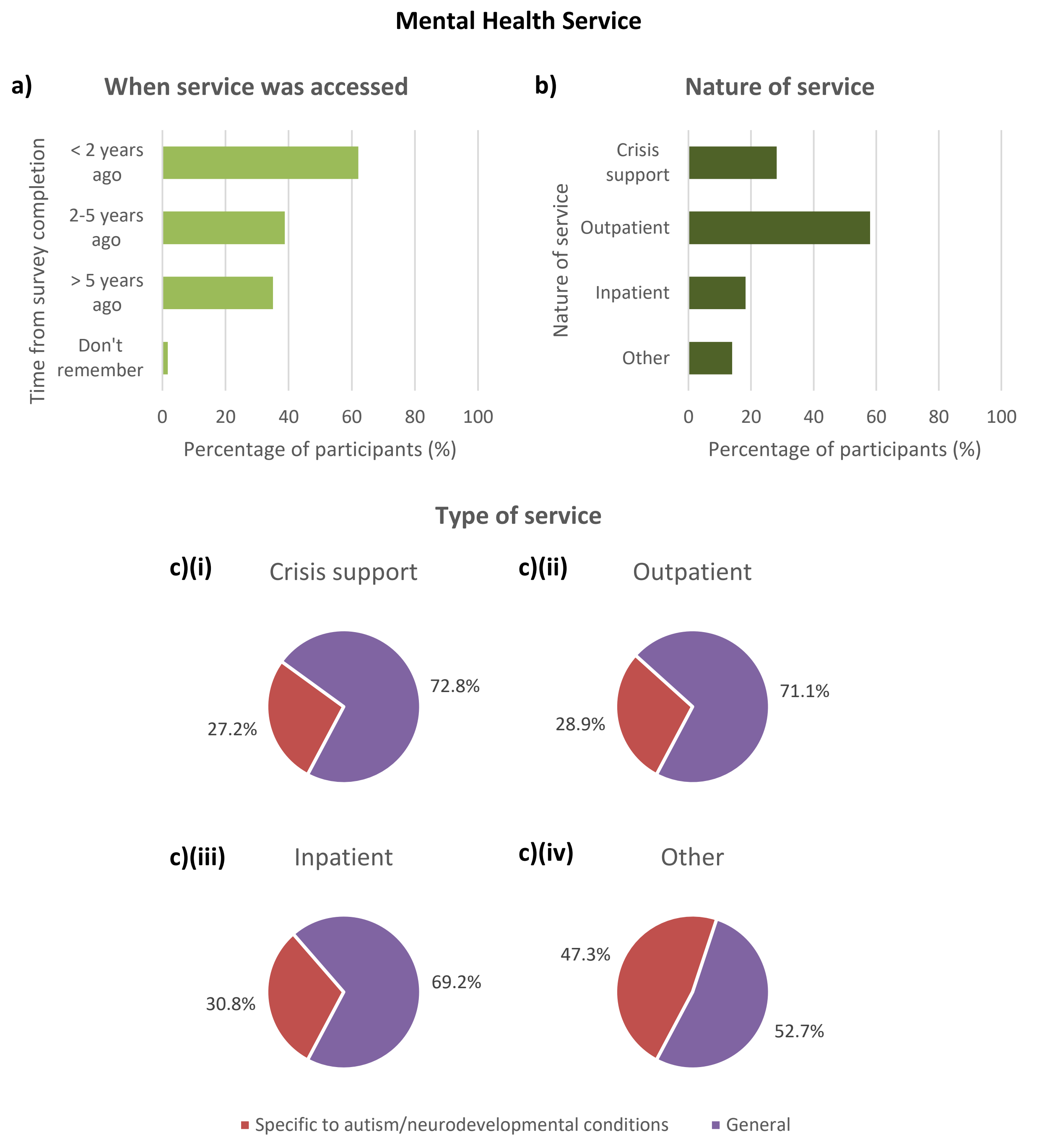

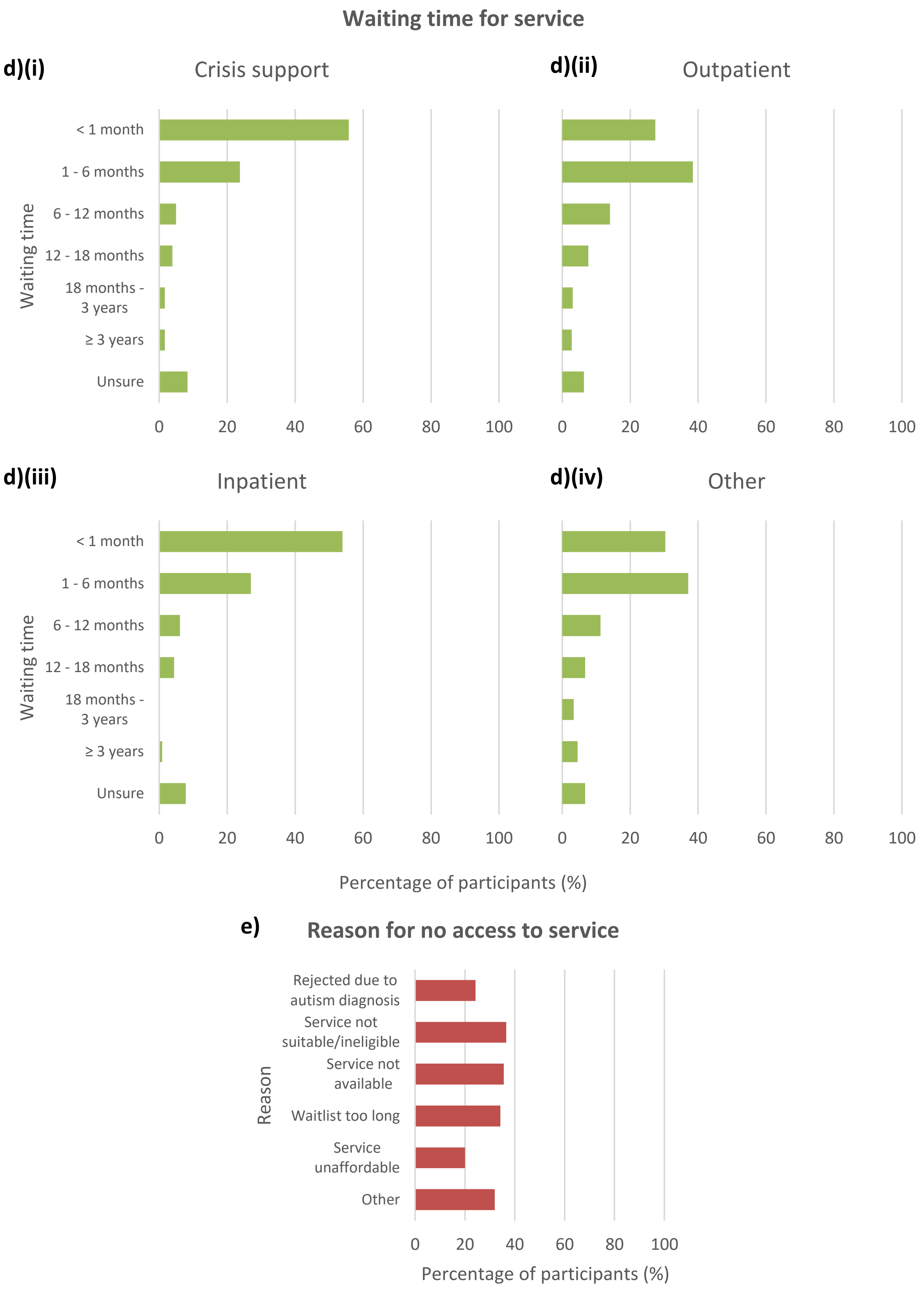
Waiting time for service
d)(i)
Crisis support
d)(ii)
Outpatient
Waiting time
< 1 month
1 - 6 months
6 - 12 months
12 - 18 months
18 months - 3 years
≥ 3 years
Unsure
0
20
40
60
80
100
d)(iii)
Inpatient
d)(iv)
Other
Percentage of participants (%)
e)
Reason for no access to service
Reason
Rejected due to autism diagnosis
Service not suitable/ineligible
Service not available
Waitlist too long
Service unaffordable
Other
Percentage of participants (%)

Supplementary Fig. 9. Mental health service. **a)** When service was last accessed from time of survey completion. Multiple responses allowed if service was accessed more than once, n=1,117. **b)** Nature of service accessed less than two years before survey completion. Multiple responses allowed if service was accessed more than once, n=696. **c)** Type of service accessed less than two years before survey completion for **(i)** crisis support, n=191, **(ii)** outpatient support, n=398, **(iii)** inpatient psychiatric service, n=120 and **(iv)** other mental health service, n=93. Only one response allowed. **d)** Waiting time upon referral for service accessed less than two years before survey completion for **(i)** crisis support, n=181, **(ii)** outpatient support, n=390, **(iii)** inpatient psychiatric service, n=115 and **(iv)** other mental health mental service, n=89. Only one response allowed. **e)** Reason for failed attempt at accessing service made less than two years before survey completion, according to participants. Multiple responses allowed, n=219.

Supplementary Figure 10

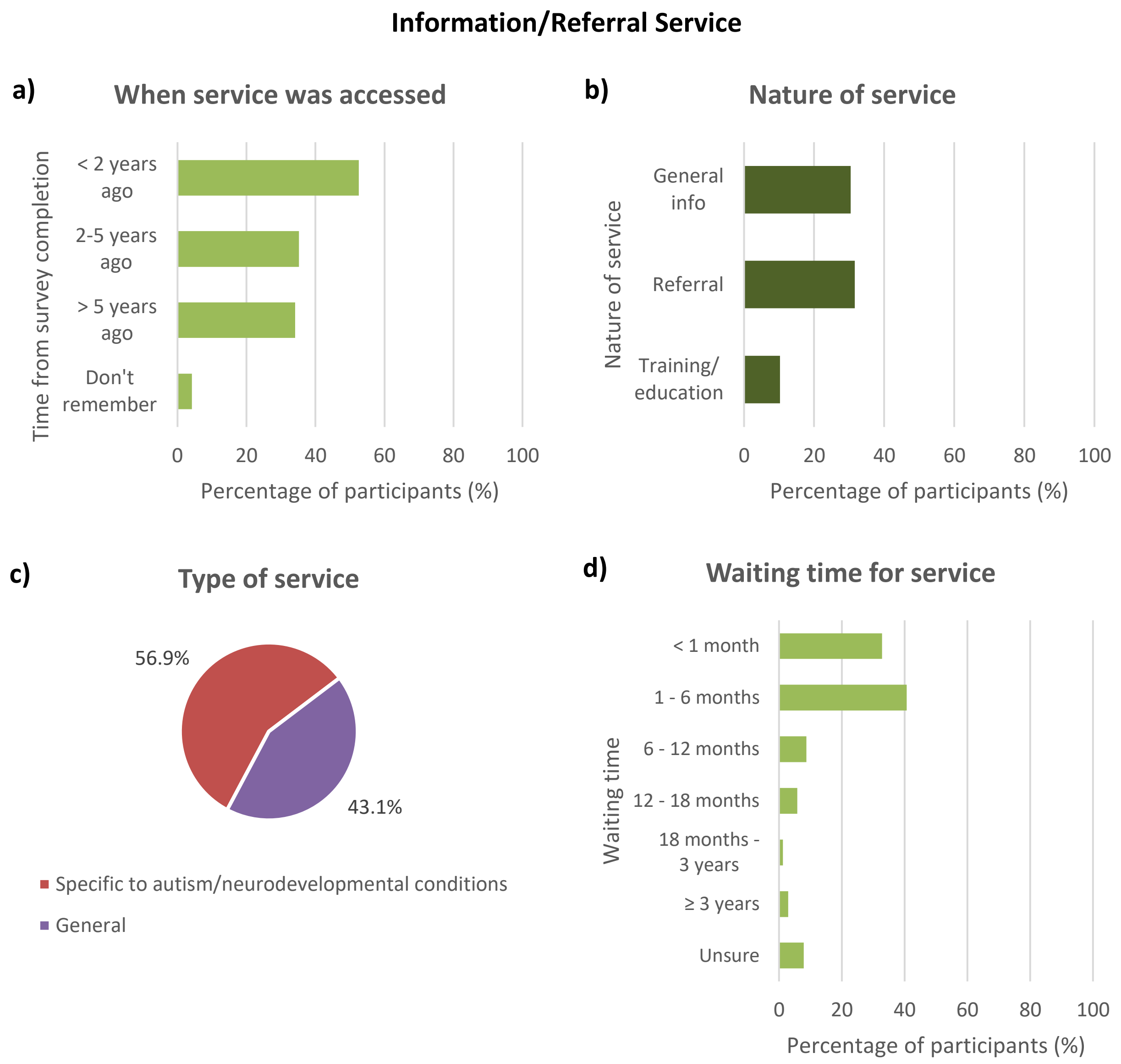

e)

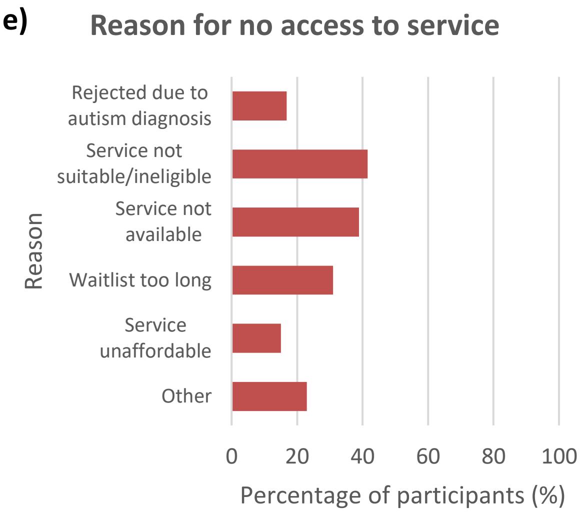

Supplementary Fig. 10. Information/referral service. **a)** When service was last accessed from time of survey completion. Multiple responses allowed if service was accessed more than once, n=651. **b)** Nature of service accessed less than two years before survey completion. Multiple responses allowed if service was accessed more than once, n=247. **c)** Type of service accessed less than two years before survey completion. Only one response allowed, n=246. **d)** Waiting time upon referral for service accessed less than two years before survey completion. Only one response allowed, n=241. **e)** Reason for failed attempt at accessing service made less than two years before survey completion, according to participants. Multiple responses allowed, n=113.

Supplementary Figure 11

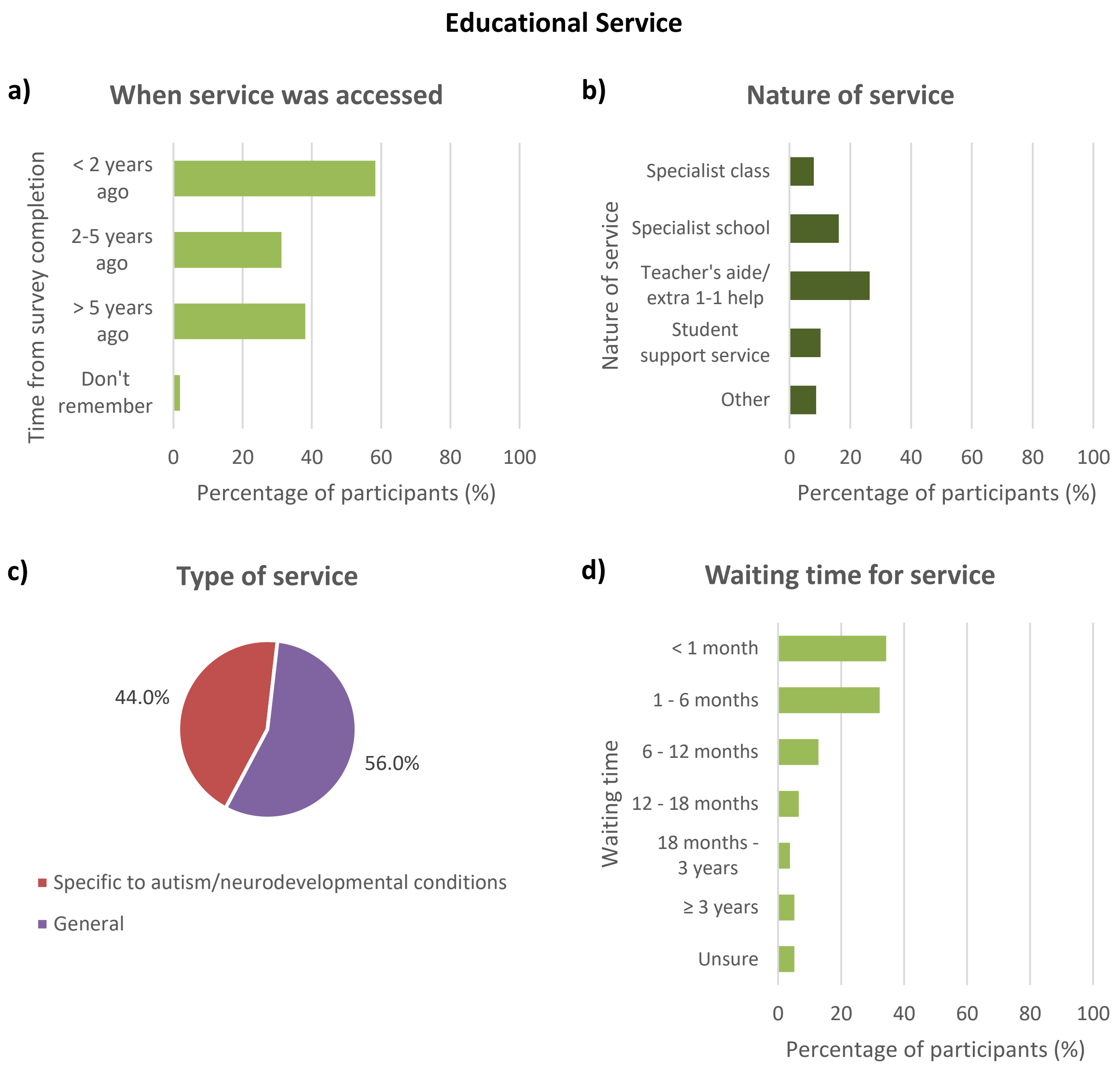

e)

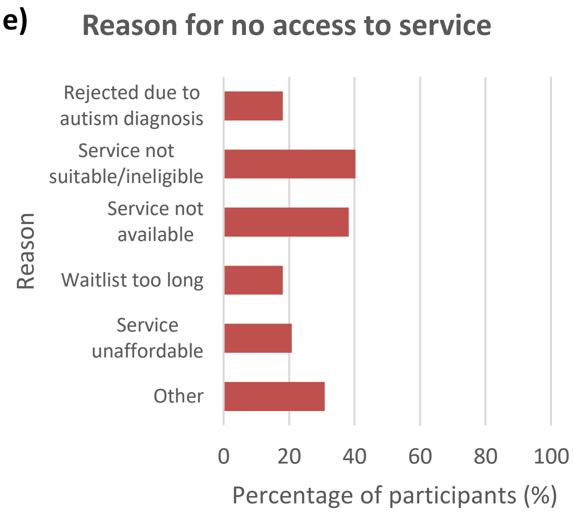

Supplementary Fig. 11. Educational service. **a)** When service was last accessed from time of survey completion. Multiple responses allowed if service was accessed more than once, n=1,077. **b)** Nature of service accessed less than two years before survey completion. Multiple responses allowed if service was accessed more than once, n=437. **c)** Type of service accessed less than two years before survey completion. Only one response allowed, n=436. **d)** Waiting time upon referral for service accessed less than two years before survey completion. Only one response allowed, n=428. **e)** Reason for failed attempt at accessing service made less than two years before survey completion, according to participants. Multiple responses allowed, n=149.

Supplementary Figure 12

Housing Service

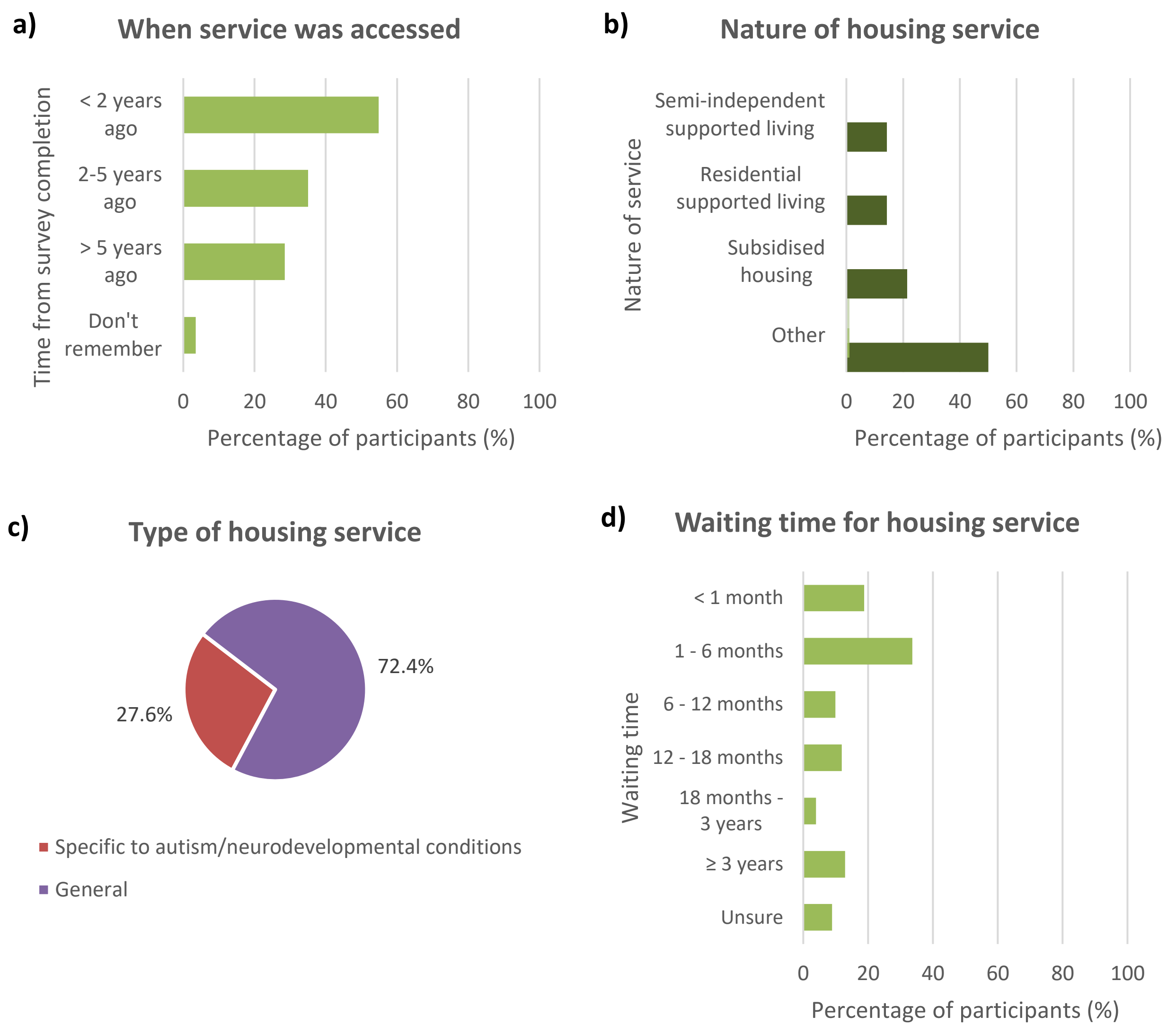

e)

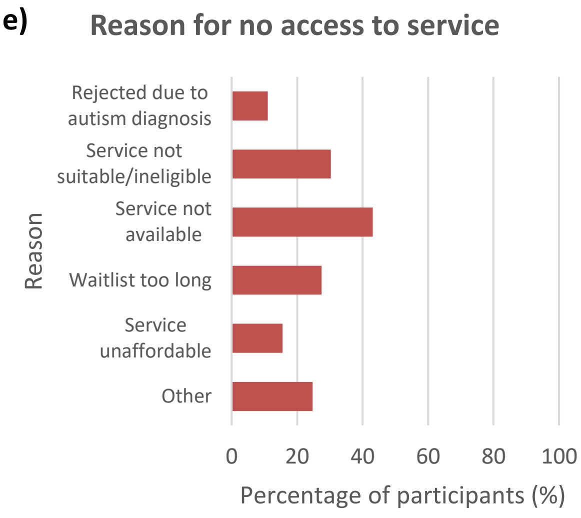

Supplementary Fig. 12. Housing service. **a)** When service was last accessed from time of survey completion. Multiple responses allowed if service was accessed more than once, n=288. **b)** Nature of service accessed less than two years before survey completion. Multiple responses allowed if service was accessed more than once, n=98. **c)** Type of service accessed less than two years before survey completion. Only one response allowed, n=105. **d)** Waiting time upon referral for service accessed less than two years before survey completion. Only one response allowed, n=101. **e)** Reason for failed attempt at accessing service made less than two years before survey completion, according to participants. Multiple responses allowed, n=109.

Supplementary Figure 13

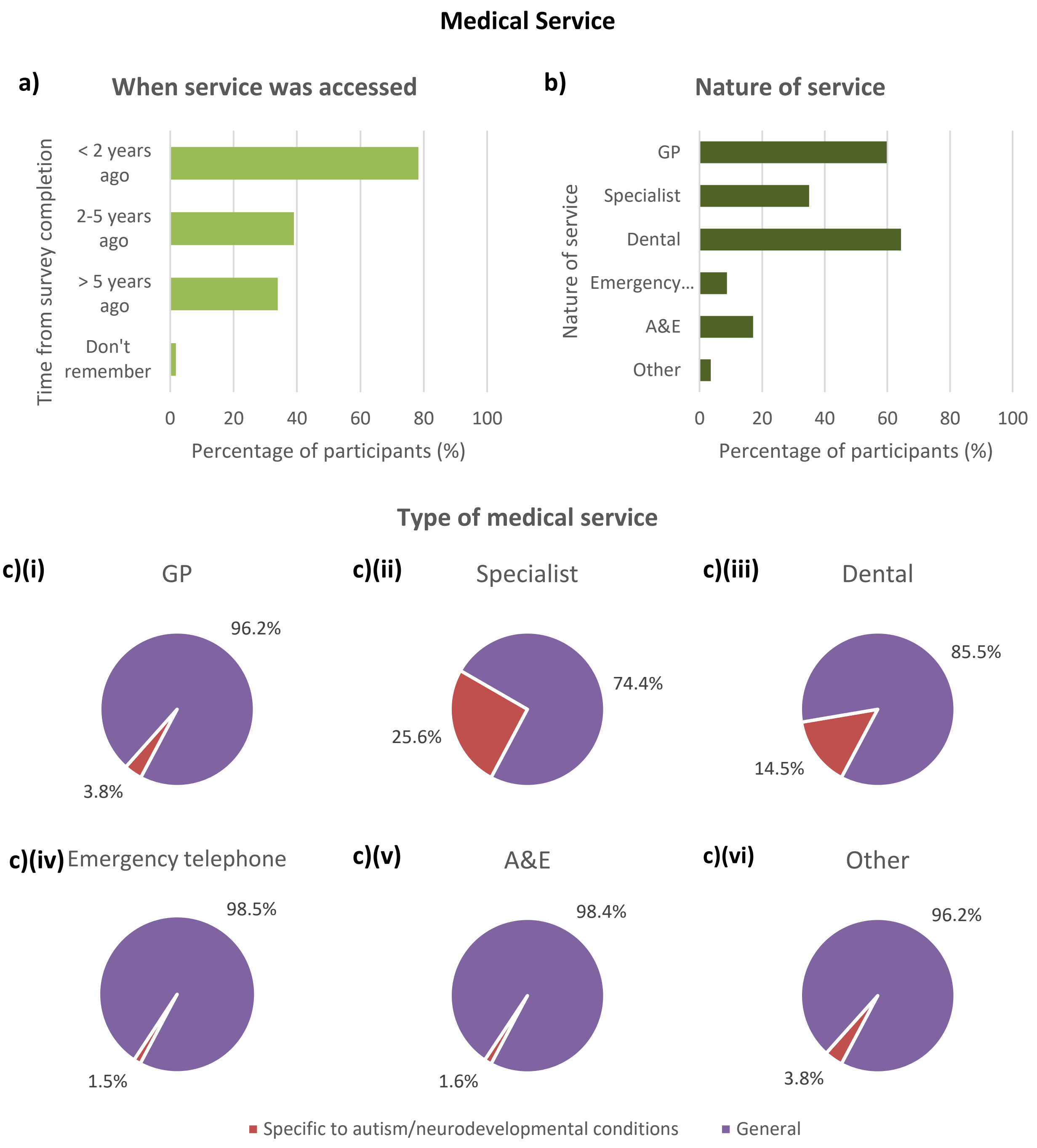

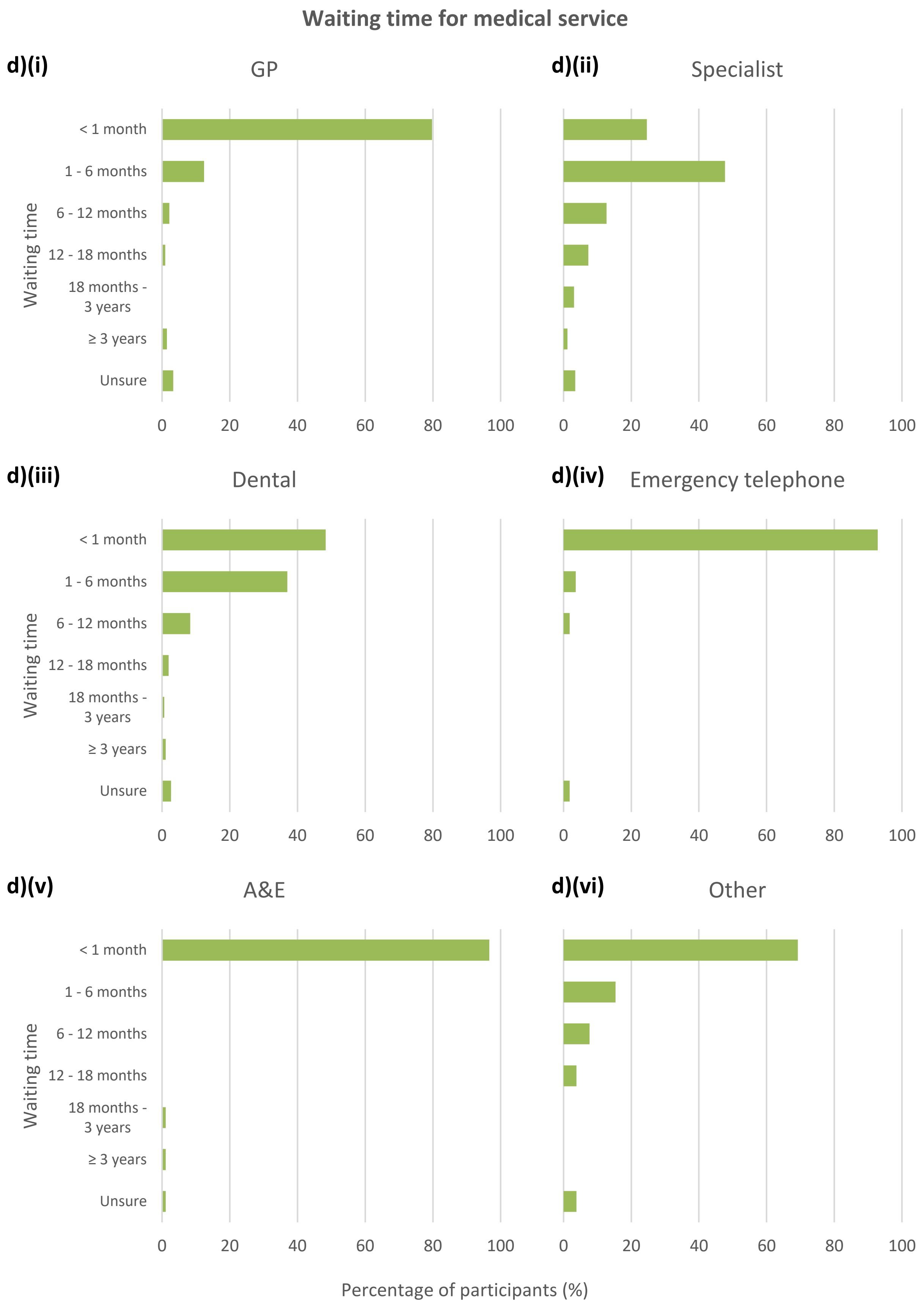
Waiting time for medical service
d)(i)
GP
d)(ii)
Specialist
d)(iii)
Dental
d)(iv)
Emergency telephone
d)(v)
A&E
d)(vi)
Other
Waiting time
< 1 month
1 - 6 months
6 - 12 months
12 - 18 months
18 months - 3 years
≥ 3 years
Unsure
0
20
40
60
80
100
Percentage of participants (%)

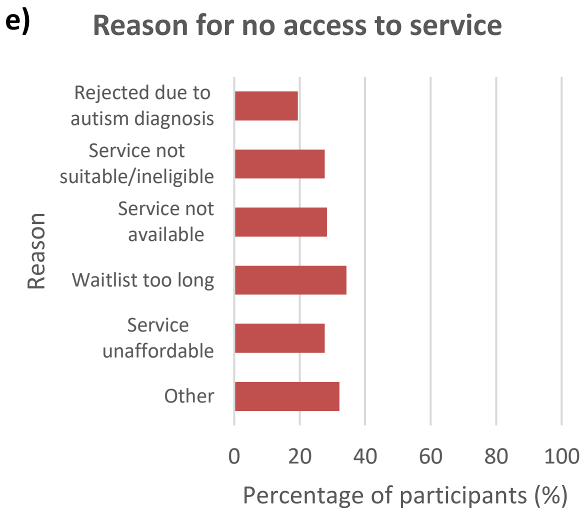

Supplementary Fig. 13. Medical service. **a)** When service was last accessed from time of survey completion. Multiple responses allowed if service was accessed more than once, n=1,017. **b)** Nature of service accessed less than two years before survey completion. Multiple responses allowed if service was accessed more than once, n=799. **c)** Type of service accessed less than two years before survey completion for **(i)** general practitioner (GP) service, n=473, **(ii)** specialist medical service, n=270, **(iii)** dental service, n=495, **(iv)** emergency telephone service, n=68, **(v)** accident and emergency (A&E) service, n=129 and **(vi)** other medical service, n=26. Only one response allowed. **d)** Waiting time upon referral for service accessed less than two years before survey completion for **(i)** general practitioner (GP) service, n=420, **(ii)** specialist medical service, n=260, **(iii)** dental service, n=457, **(iv)** emergency telephone service, n=56, **(v)** accident and emergency (A&E) service, n=90 and **(vi)** other medical service, n=26. Only one response allowed. **e)** Reason for failed attempt at accessing service made less than two years before survey completion, according to participants. Multiple responses allowed, n=134.

Supplementary Figure 14

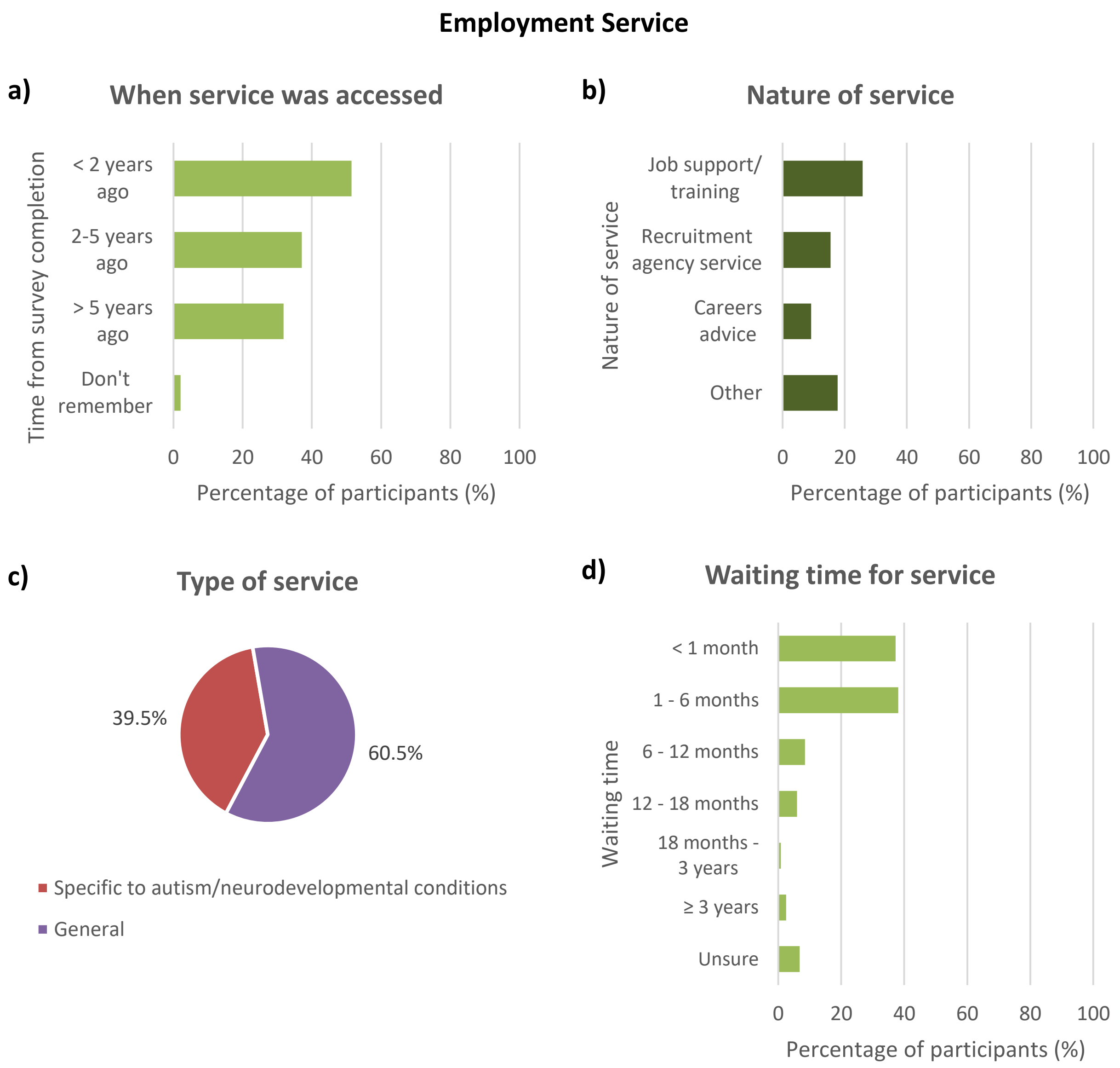

e)

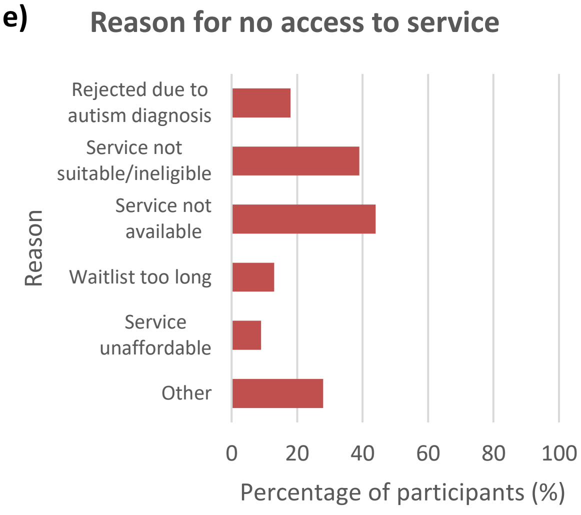

Supplementary Fig. 14. Employment service. **a)** When service was last accessed from time of survey completion. Multiple responses allowed if service was accessed more than once, n=340. **b)** Nature of service accessed less than two years before survey completion. Multiple responses allowed if service was accessed more than once, n=119. **c)** Type of service accessed less than two years before survey completion. Only one response allowed, n=119. **d)** Waiting time upon referral for service accessed less than two years before survey completion. Only one response allowed, n=118. **e)** Reason for failed attempt at accessing service made less than two years before survey completion, according to participants. Multiple responses allowed, n=100.

Supplementary Figure 15

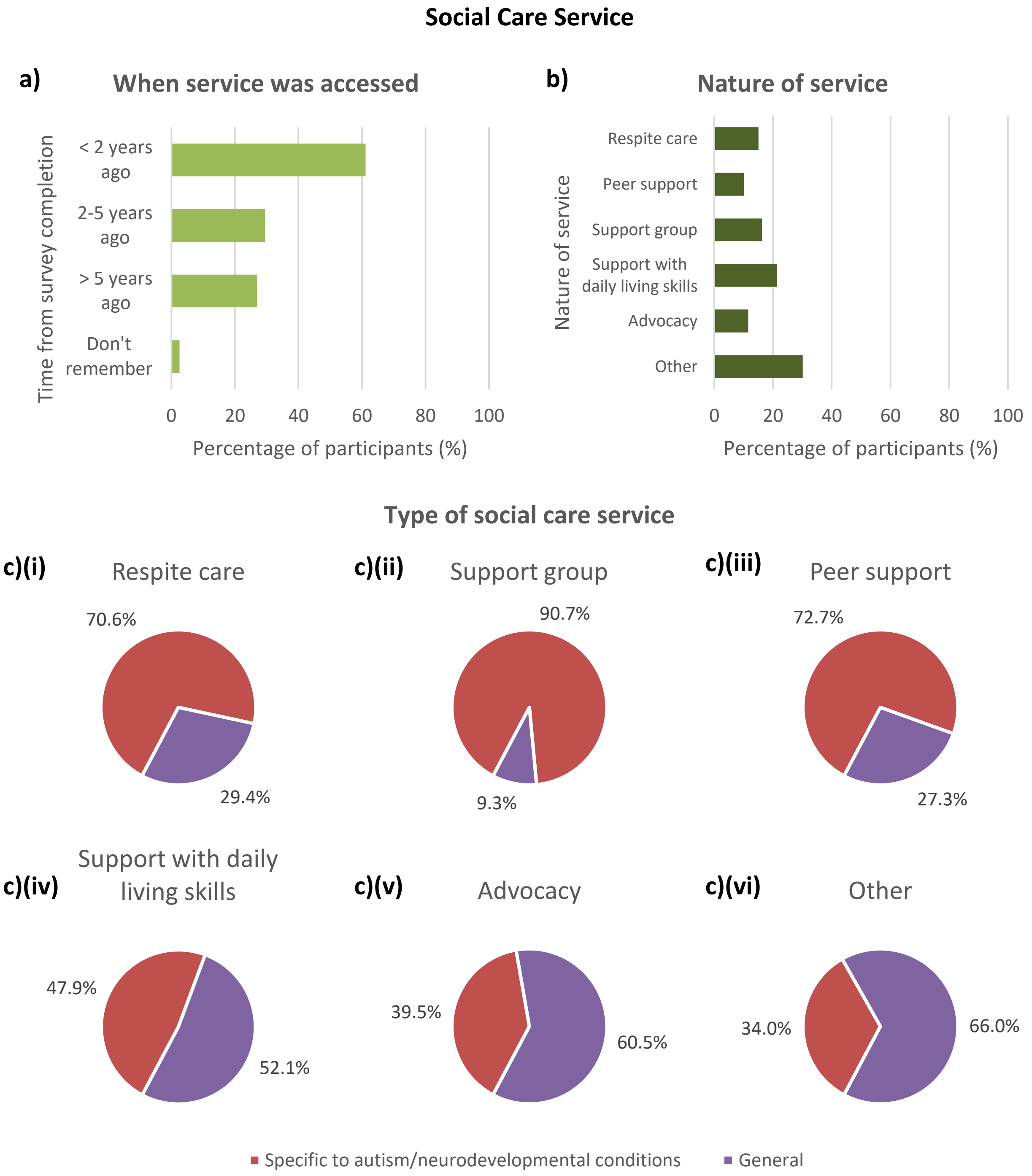

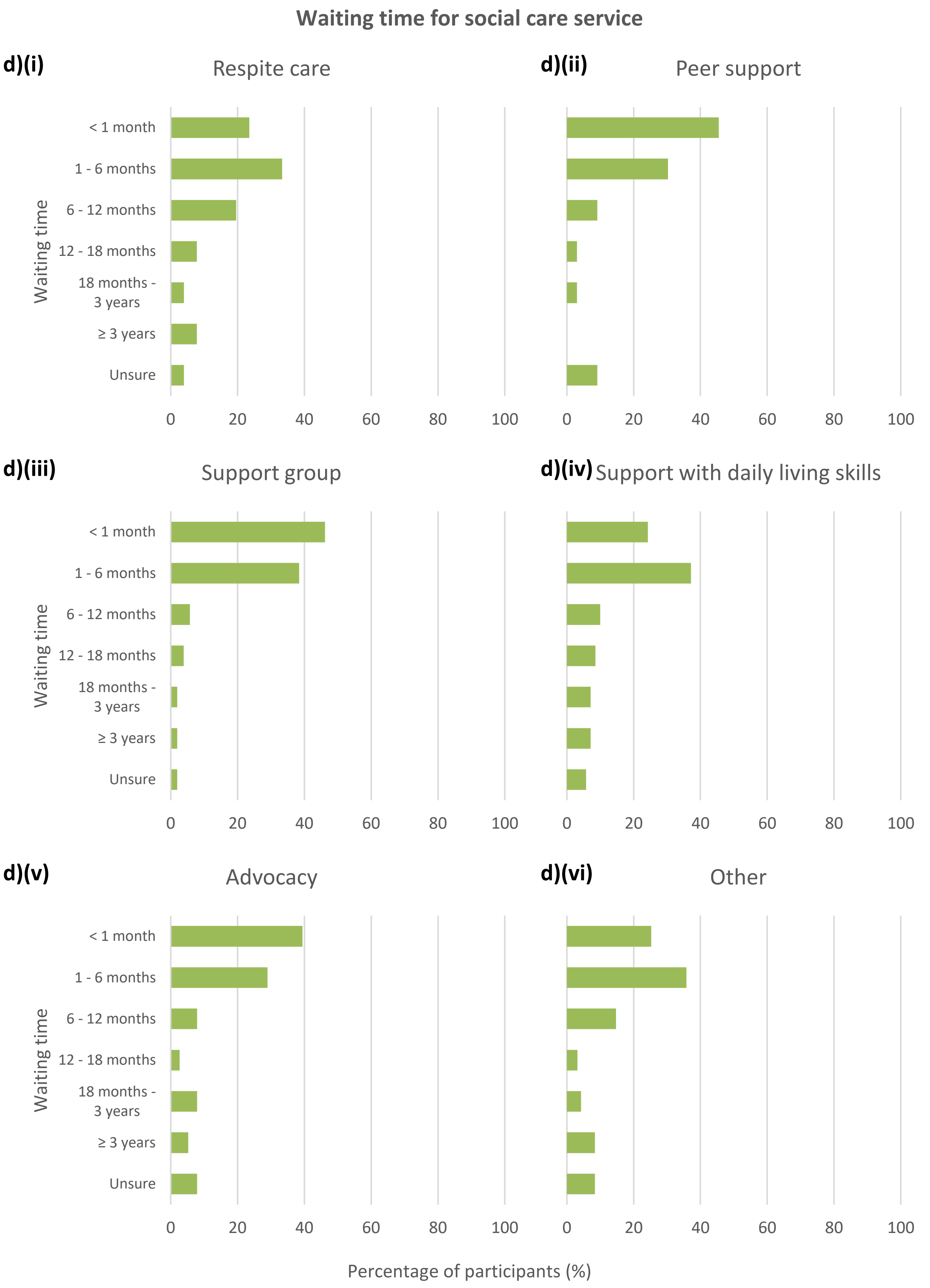
Waiting time for social care service
d)(i)
Respite care
d)(ii)
Peer support
d)(iii)
Support group
d)(iv)
Support with daily living skills
d)(v)
Advocacy
d)(vi)
Other
Waiting time
< 1 month
1 - 6 months
6 - 12 months
12 - 18 months
18 months - 3 years
≥ 3 years
Unsure
0
20
40
60
80
100
Percentage of participants (%)

e)

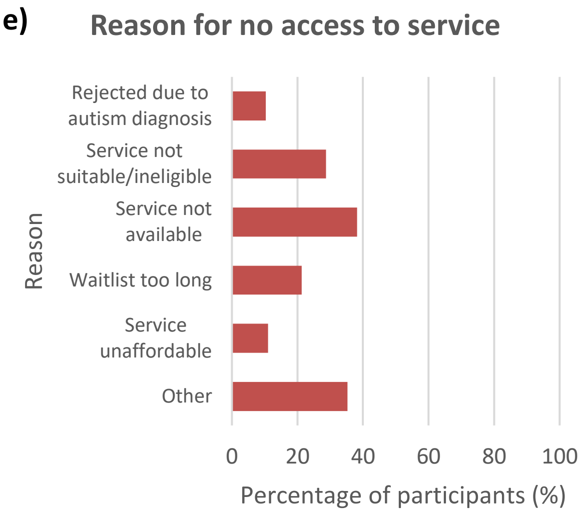

Supplementary Fig. 15. Social care service. **a)** When service was last accessed from time of survey completion. Multiple responses allowed if service was accessed more than once, n=546. **b)** Nature of service accessed less than two years before survey completion. Multiple responses allowed if service was accessed more than once, n=338. **c)** Type of service accessed less than two years before survey completion for **(i)** respite care service, n=51, **(ii)** peer support, n=33, **(iii)** support group service, n=54, **(iv)** support with daily living skills, n=71, **(v)** advocacy service, n=38 and **(vi)** other social care service, n=97. Only one response allowed. **d)** Waiting time upon referral for service accessed less than two years before survey completion for **(i)** respite care service, n=51, **(ii)** peer support, n=33, **(iii)** support group service, n=52, **(iv)** support with daily living skills, n=70, **(v)** advocacy service, n=38 and **(vi)** other social care service, n=95. Only one response allowed. **e)** Reason for failed attempt at accessing service made less than two years before survey completion, according to participants. Multiple responses allowed, n=136.

Supplementary Figure 16

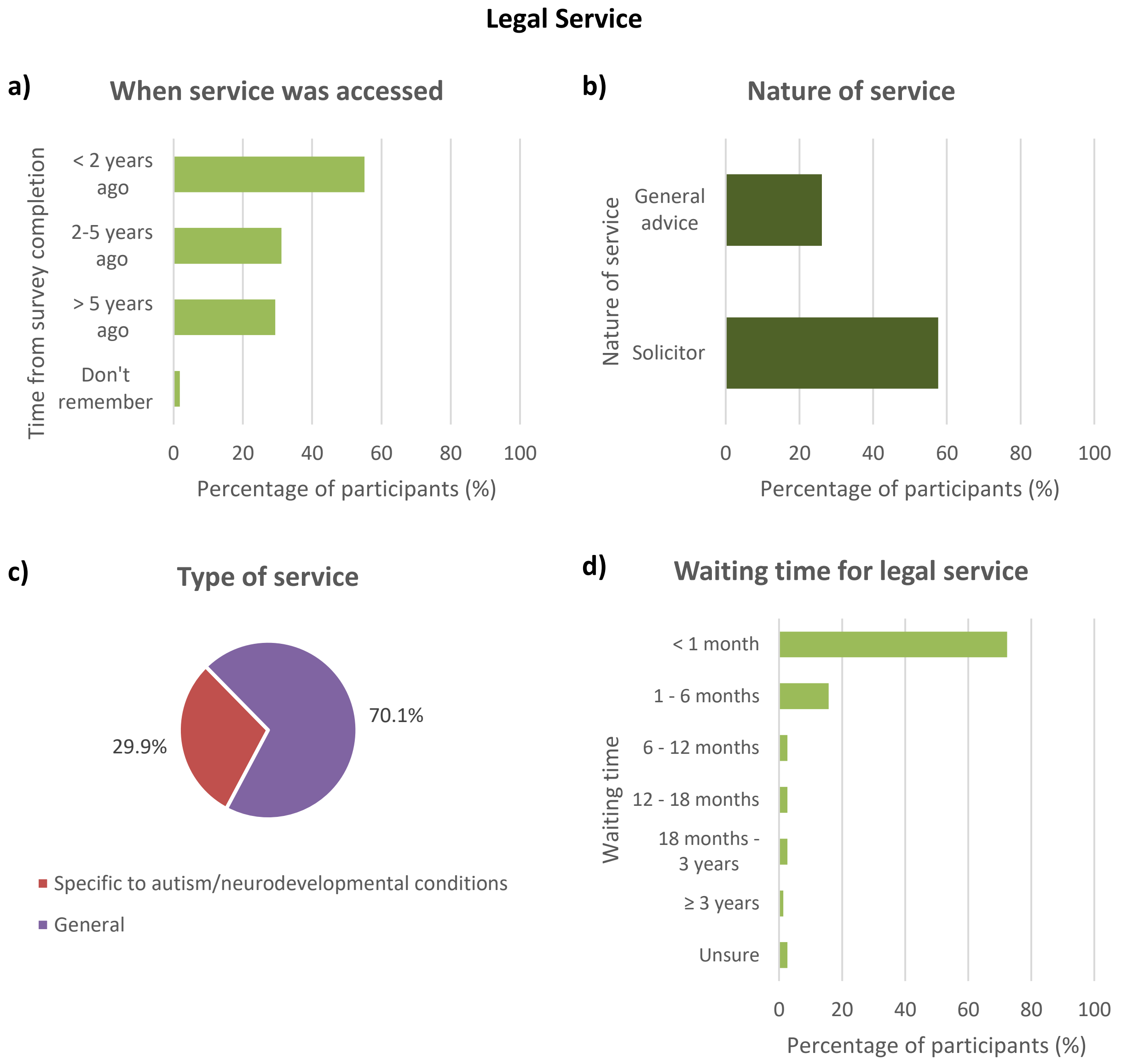

e)

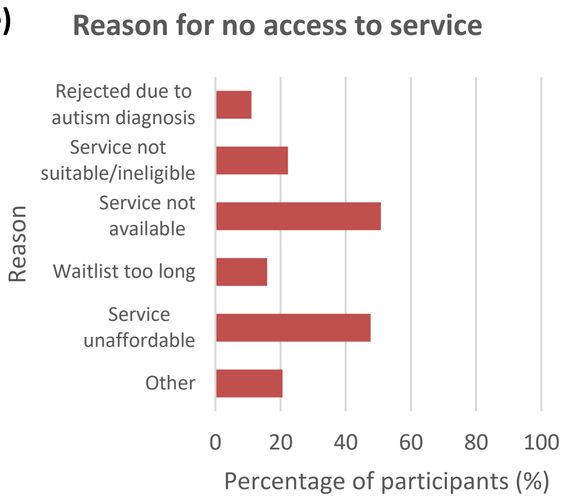

Supplementary Fig. 16. Legal service. **a)** When service was last accessed from time of survey completion. Multiple responses allowed if service was accessed more than once, n=167. **b)** Nature of service accessed less than two years before survey completion. Multiple responses allowed if service was accessed more than once, n=77. **c)** Type of service accessed less than two years before survey completion. Only one response allowed, n=77. **d)** Waiting time upon referral for service accessed less than two years before survey completion. Only one response allowed, n=76. **e)** Reason for failed attempt at accessing service made less than two years before survey completion, according to participants. Multiple responses allowed, n=63.

Supplementary Figure 17

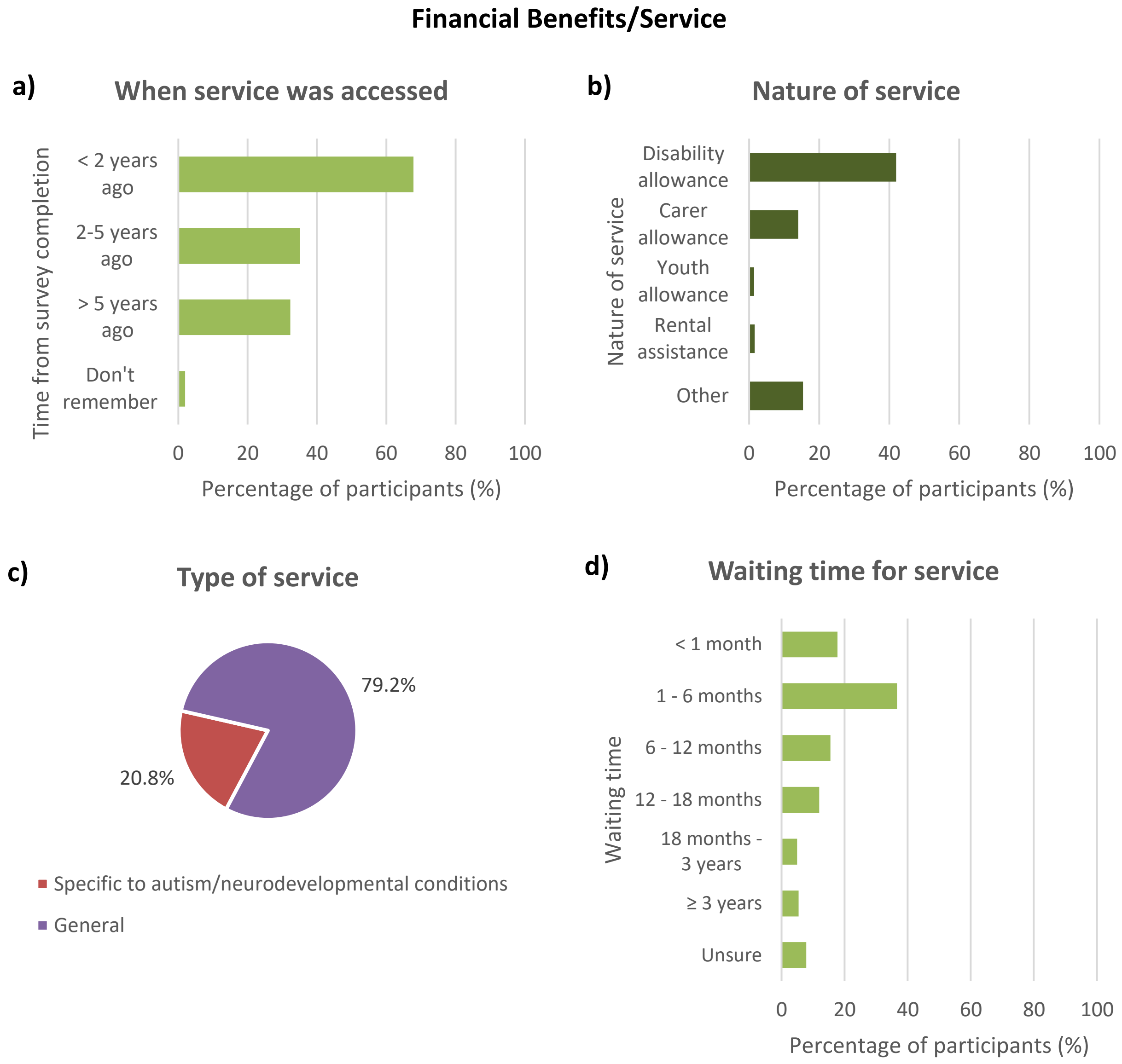

e)

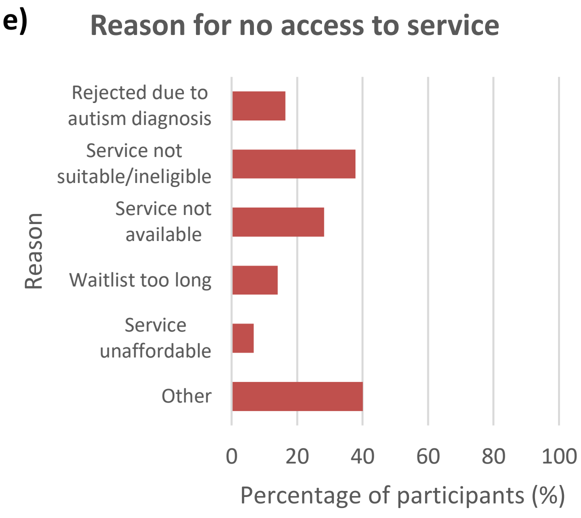

Supplementary Fig. 17. Financial benefits/service. **a)** When service was last accessed from time of survey completion. Multiple responses allowed if service was accessed more than once, n=912. **b)** Nature of service accessed less than two years before survey completion. Multiple responses allowed if service was accessed more than once, n=461. **c)** Type of service accessed less than two years before survey completion. Only one response allowed, n=457. **d)** Waiting time upon referral for service accessed less than two years before survey completion. Only one response allowed, n=445. **e)** Reason for failed attempt at accessing service made less than two years before survey completion, according to participants. Multiple responses allowed, n=177.

Supplementary Figure 18

Helpline Service

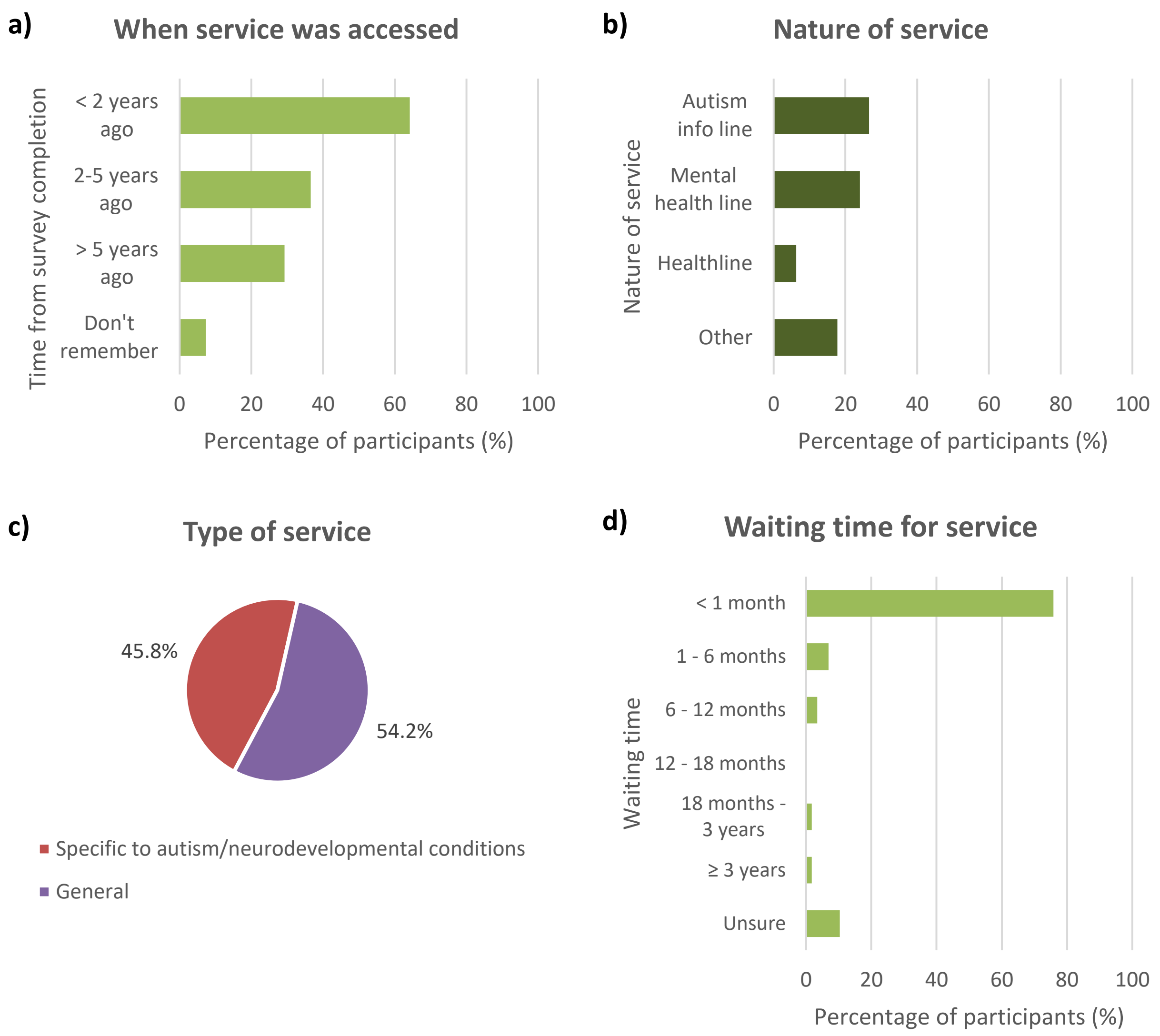

e)

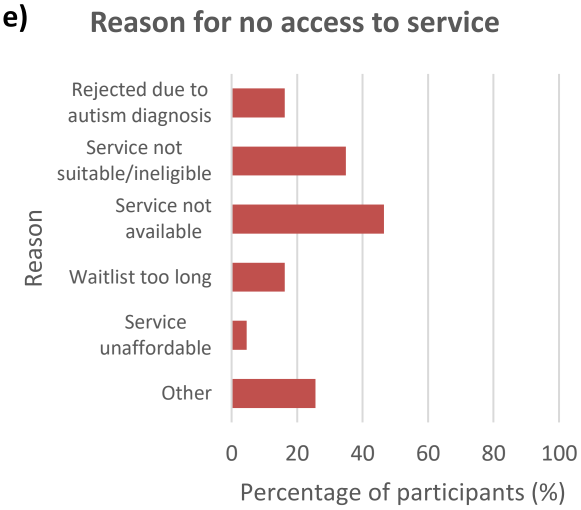

Supplementary Fig. 18. Helpline service. **a)** When service was last accessed from time of survey completion. Multiple responses allowed if service was accessed more than once, n=123. **b)** Nature of service accessed less than two years before survey completion. Multiple responses allowed if service was accessed more than once, n=59. **c)** Type of service accessed less than two years before survey completion. Only one response allowed, n=59. **d)** Waiting time upon referral for service accessed less than two years before survey completion. Only one response allowed, n=58. **e)** Reason for failed attempt at accessing service made less than two years before survey completion, according to participants. Multiple responses allowed, n=43.

Supplementary Figure 19

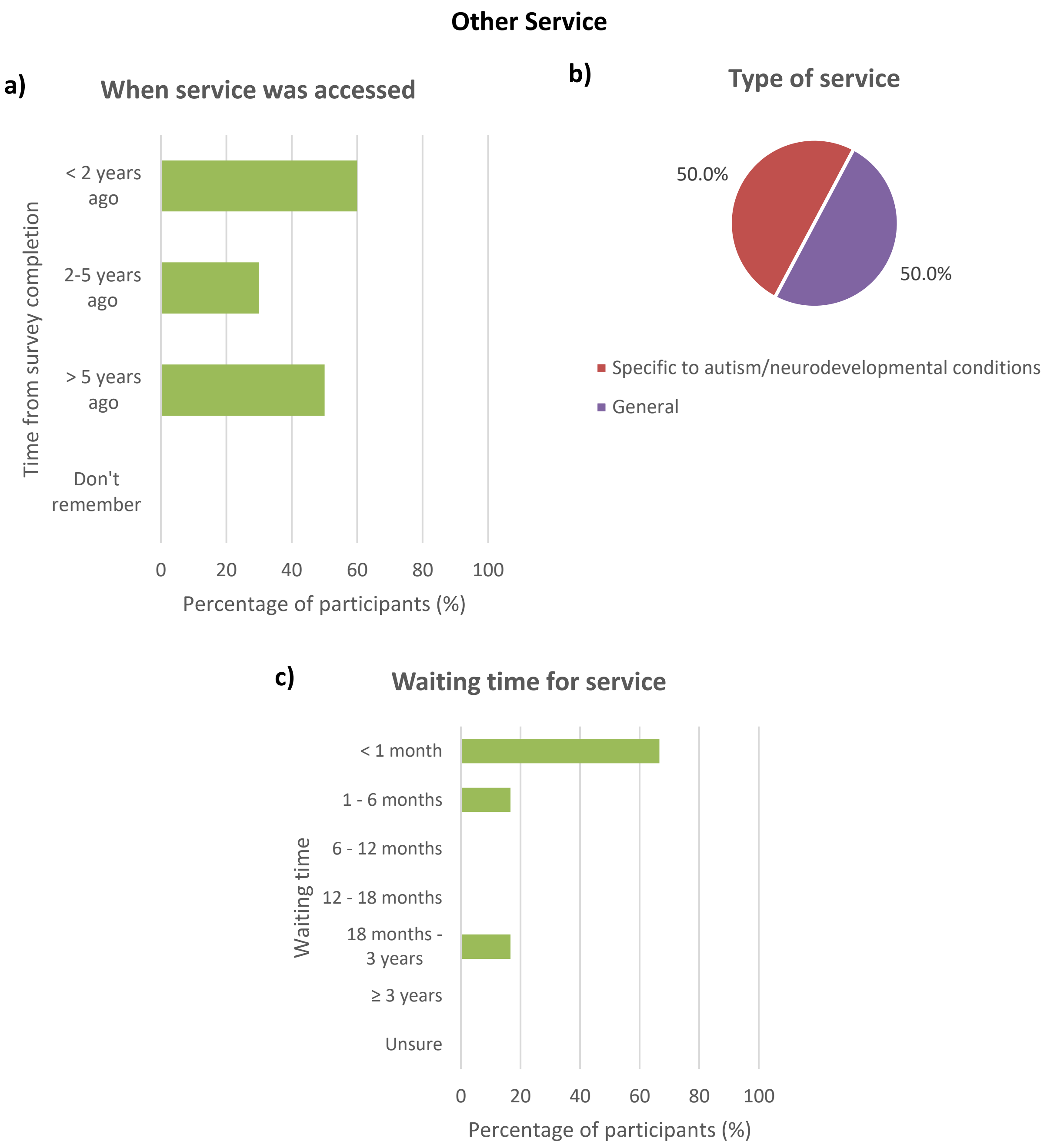

Supplementary Fig. 19. Other service not listed in survey. **a)** When service was last accessed from time of survey completion. Multiple responses allowed if service was accessed more than once, n=9. **b)** Type of service accessed less than two years before survey completion. Only one response allowed, n=6. **c)** Waiting time upon referral for service accessed less than two years before survey completion. Only one response allowed, n=6. No participant indicated failed access to service.

Supplementary Figure 20

Service Access at Any Timepoint

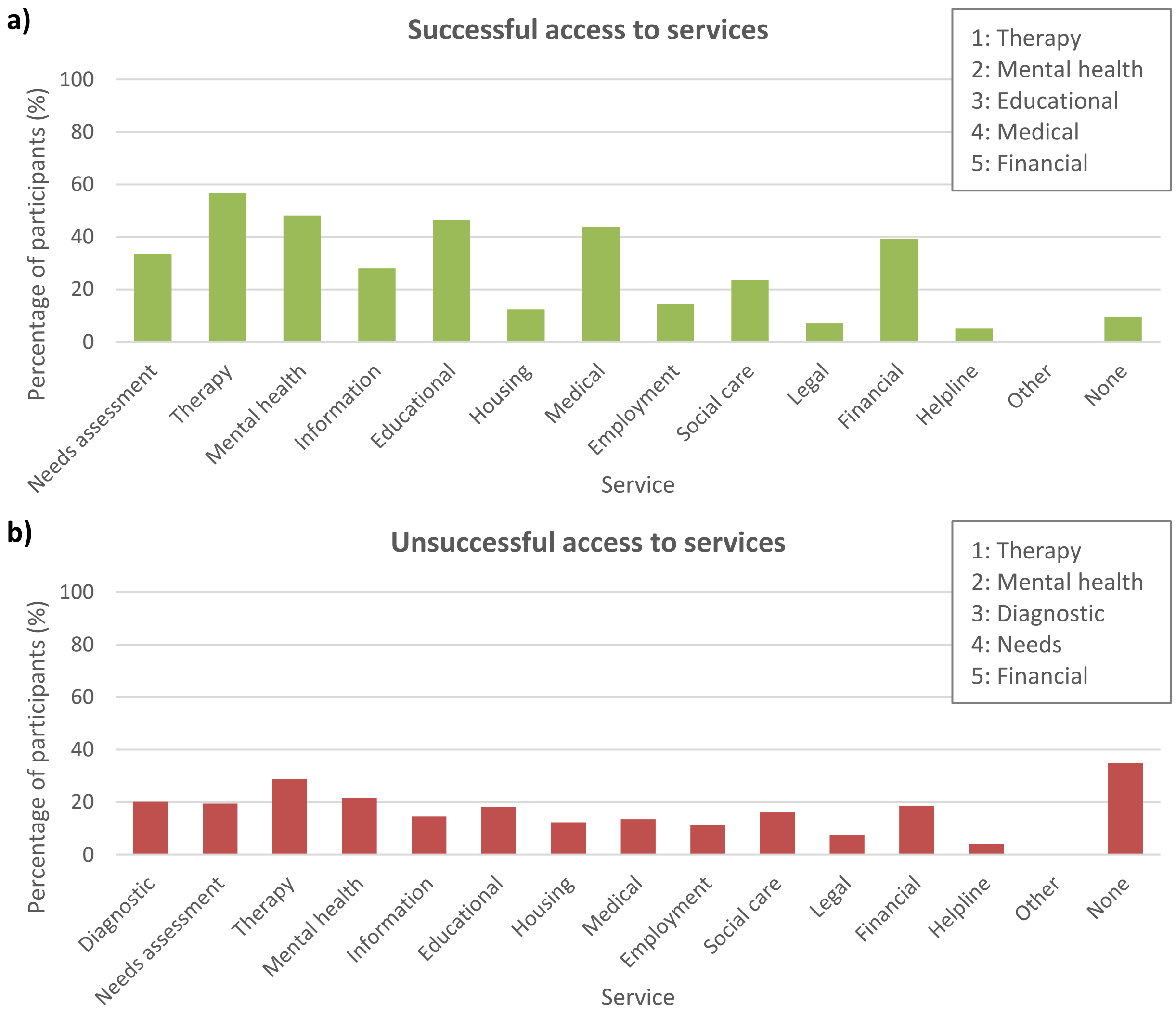

Supplementary Fig. 20. Percentage of participants indicating **a)** previous access at any timepoint to needs assessment, therapy, mental health, information/referral, educational, housing, medical, employment, social care, legal, financial and helpline services as well as other services not listed in the survey and none of the services listed, n=2,322, and **b)** unsuccessful attempts at any timepoint at access to autism diagnostic, needs assessment, therapy, mental health, information/referral, educational, housing, medical, employment, social care, legal, financial and helpline services as well as other services not listed in the survey and none of the services listed, n=1,840.

Supplementary Figure 21

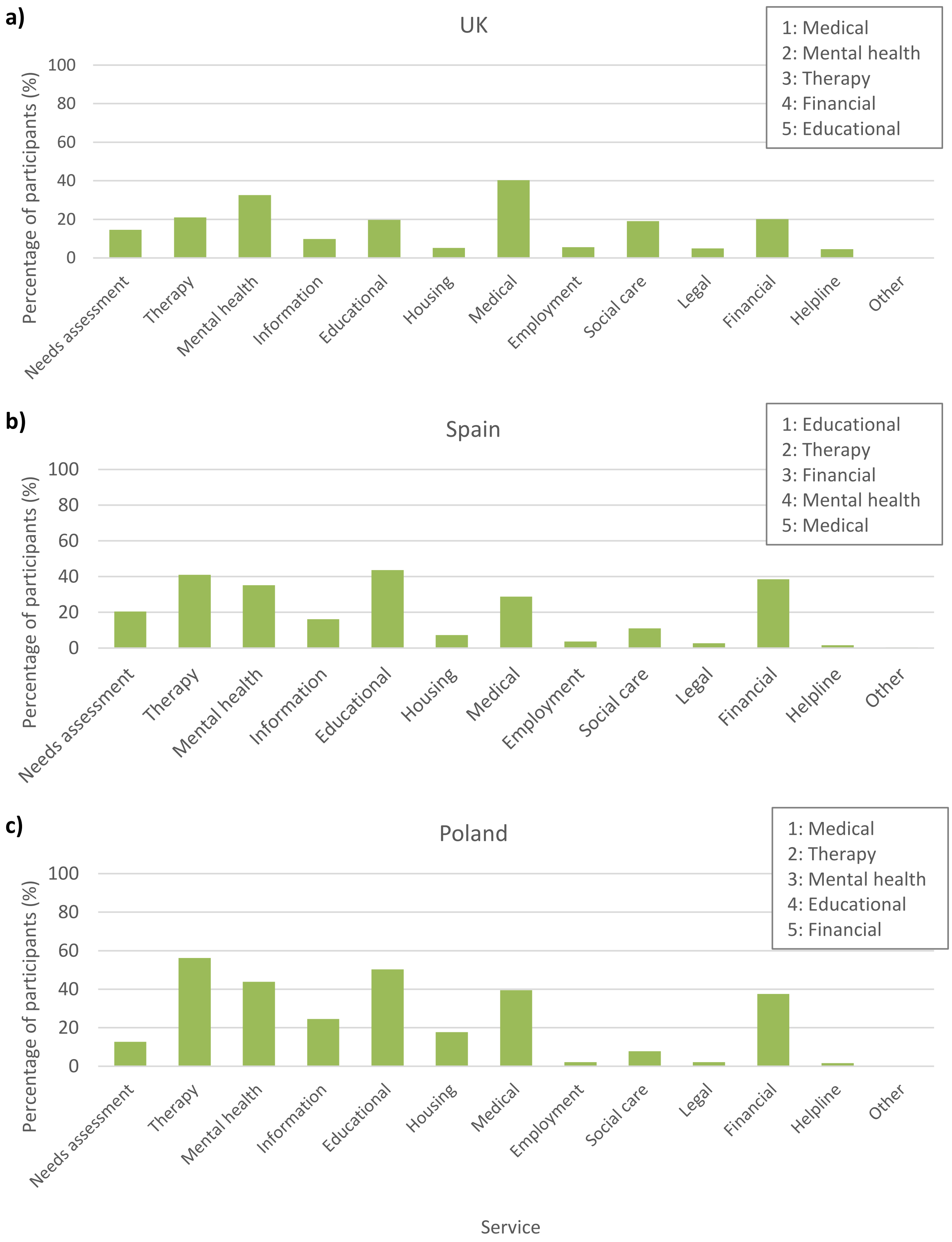

d)

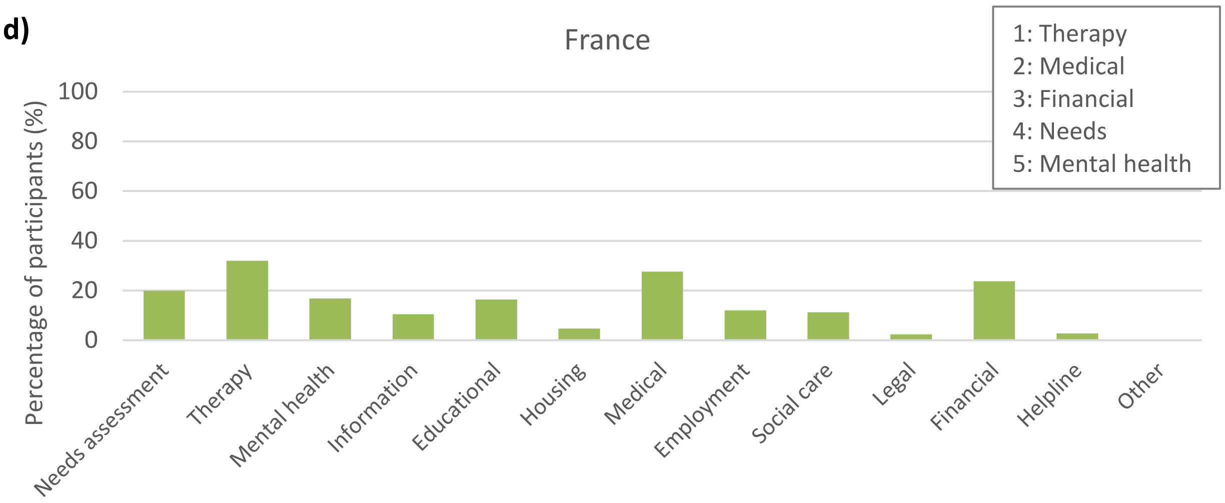

e)

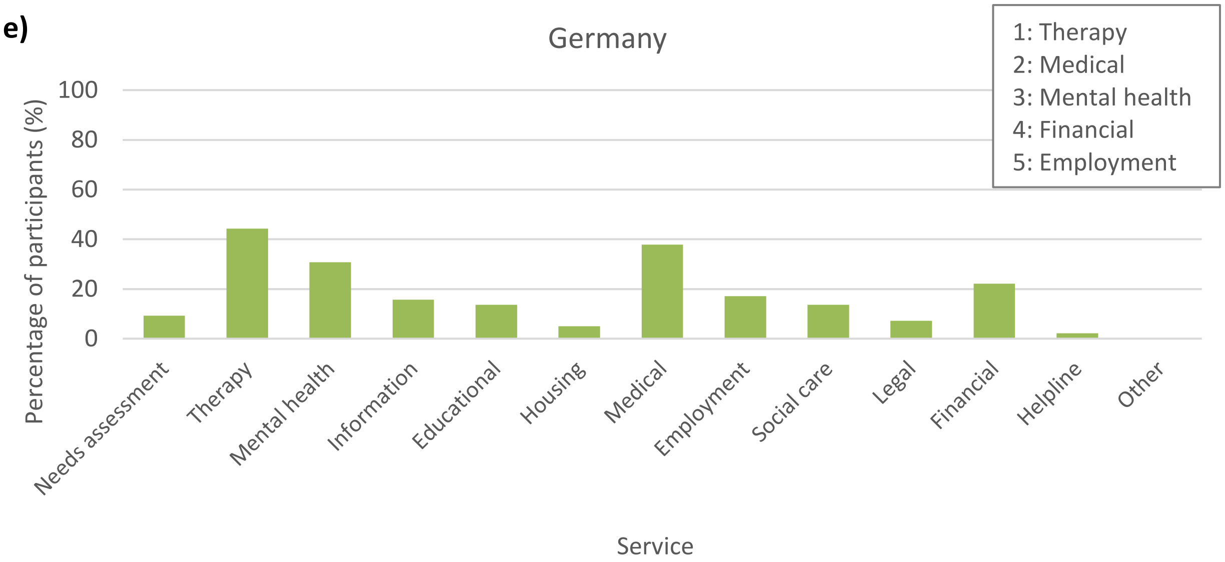

Supplementary Fig. 21. Percentage of participants indicating access less than two years before survey completion to needs assessment, therapy, mental health, information/referral, educational, housing, medical, employment, social care, legal, financial and helpline services as well as other services not listed in the survey in **a)** the UK, n=774, **b)** Spain, n=326, **c)** Poland, n=322, **d)** France, n=257 and **e)** Germany, n=140.

Supplementary Figure 22

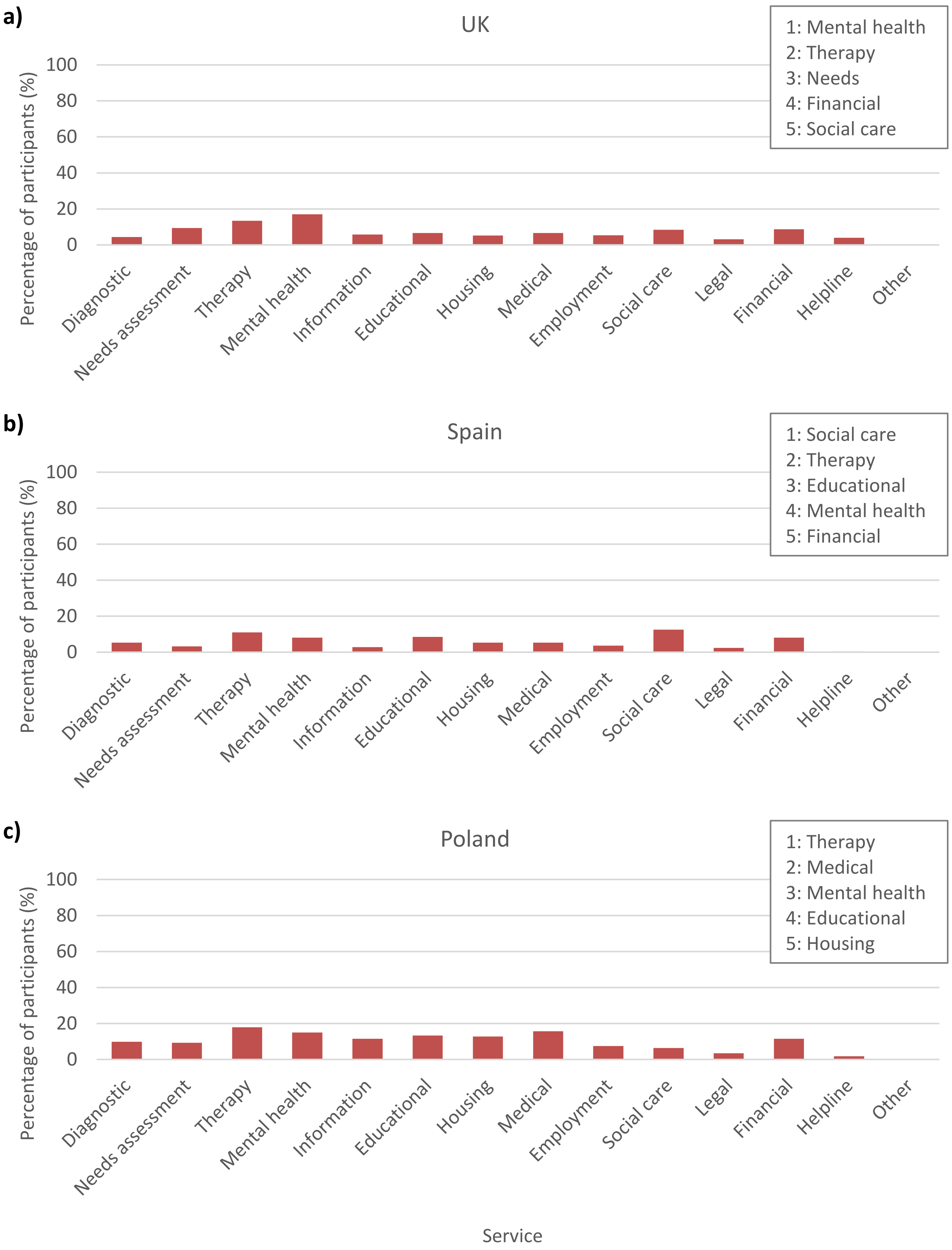

d)

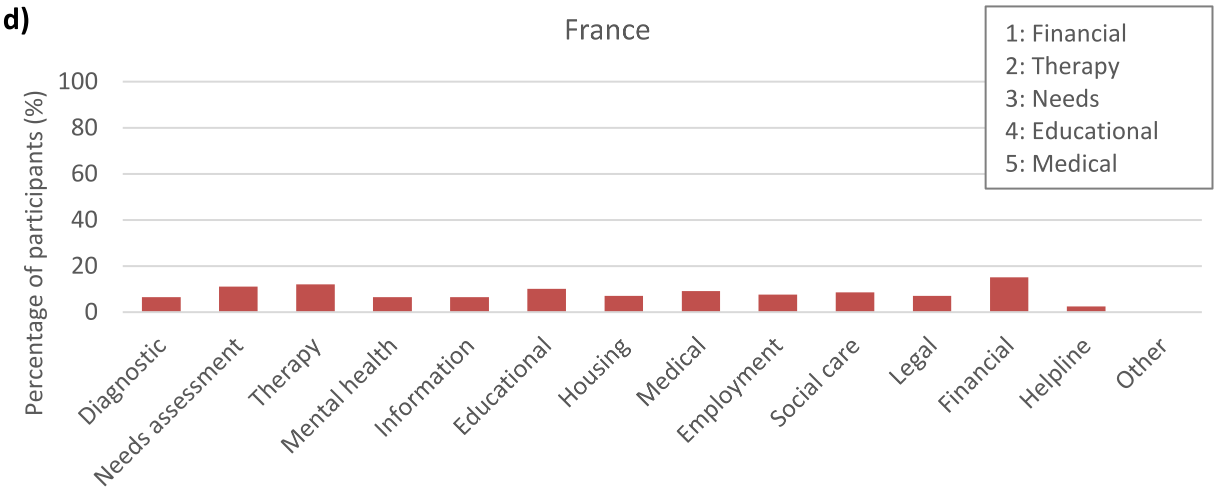

e)

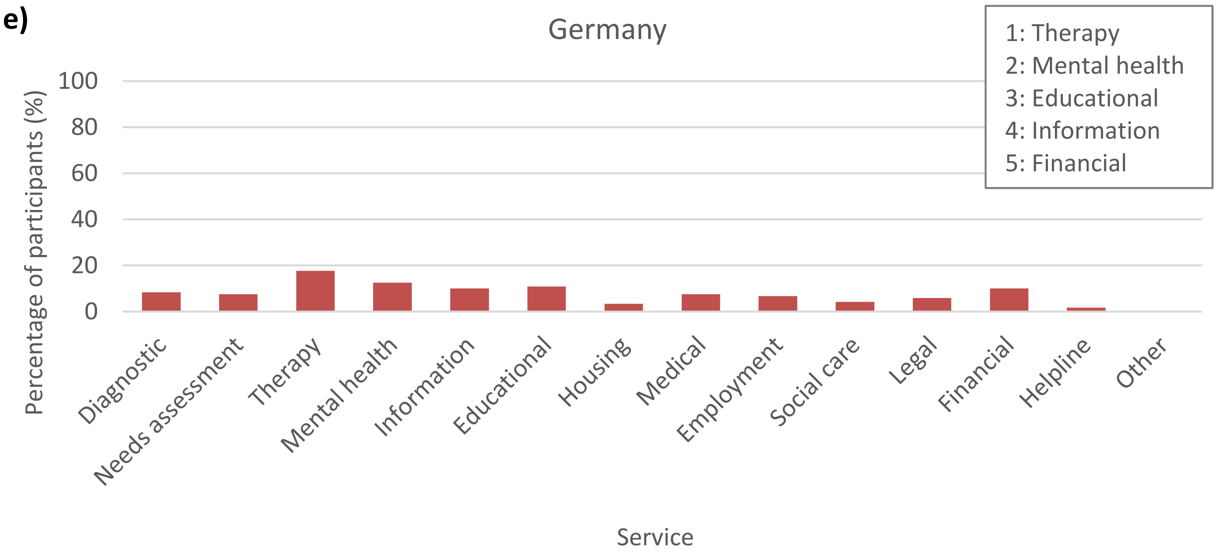

Supplementary Fig. 22. Percentage of participants indicating unsuccessful attempts at access less than two years before survey completion to autism diagnostic, needs assessment, therapy, mental health, information/referral, educational, housing, medical, employment, social care, legal, financial and helpline services as well as other services not listed in the survey in **a)** the UK, n=686, **b)** Spain, n=247, **c)** Poland, n=173, **d)** France, n=198 and **e)** Germany, n=119.

Supplementary Figure 23

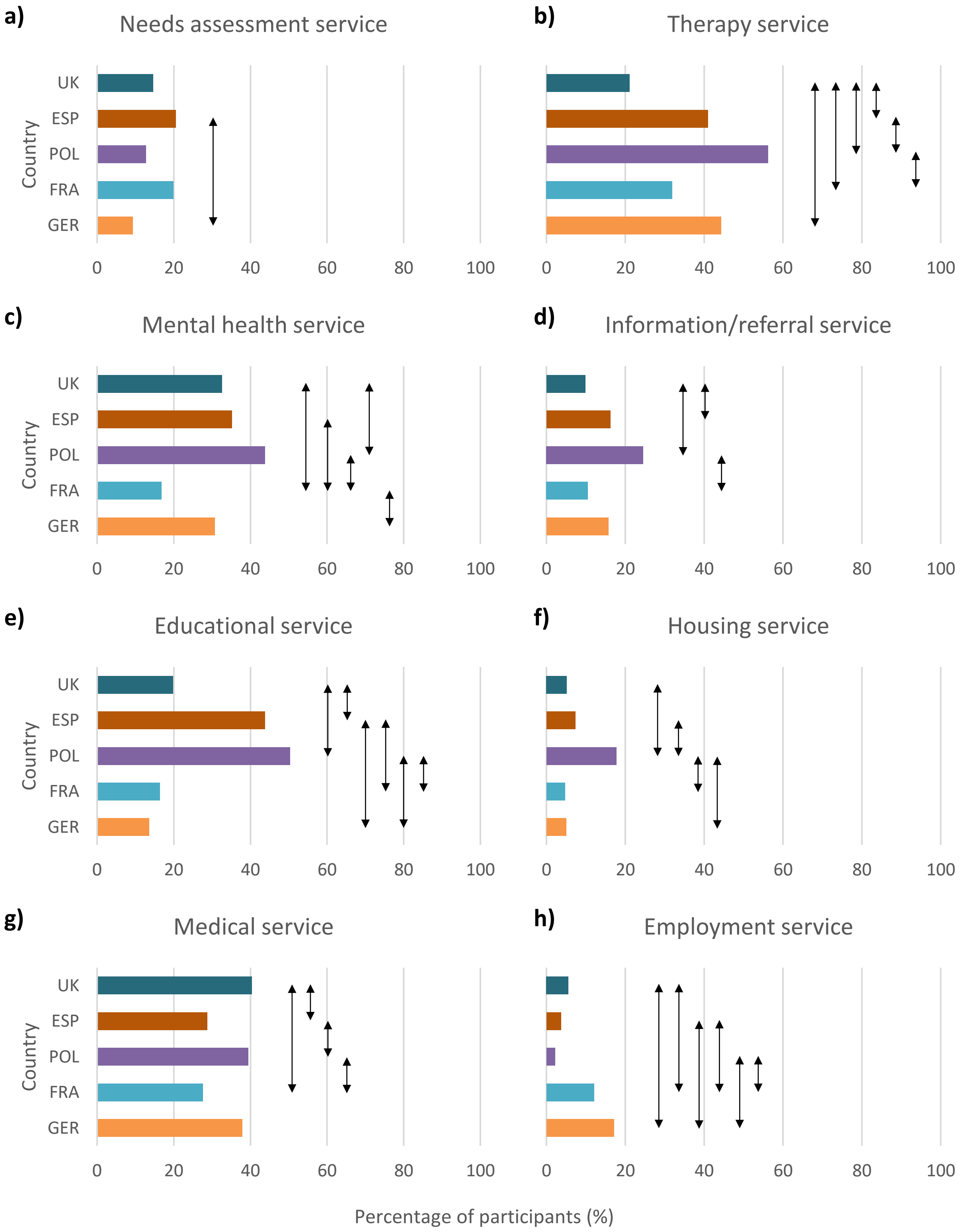

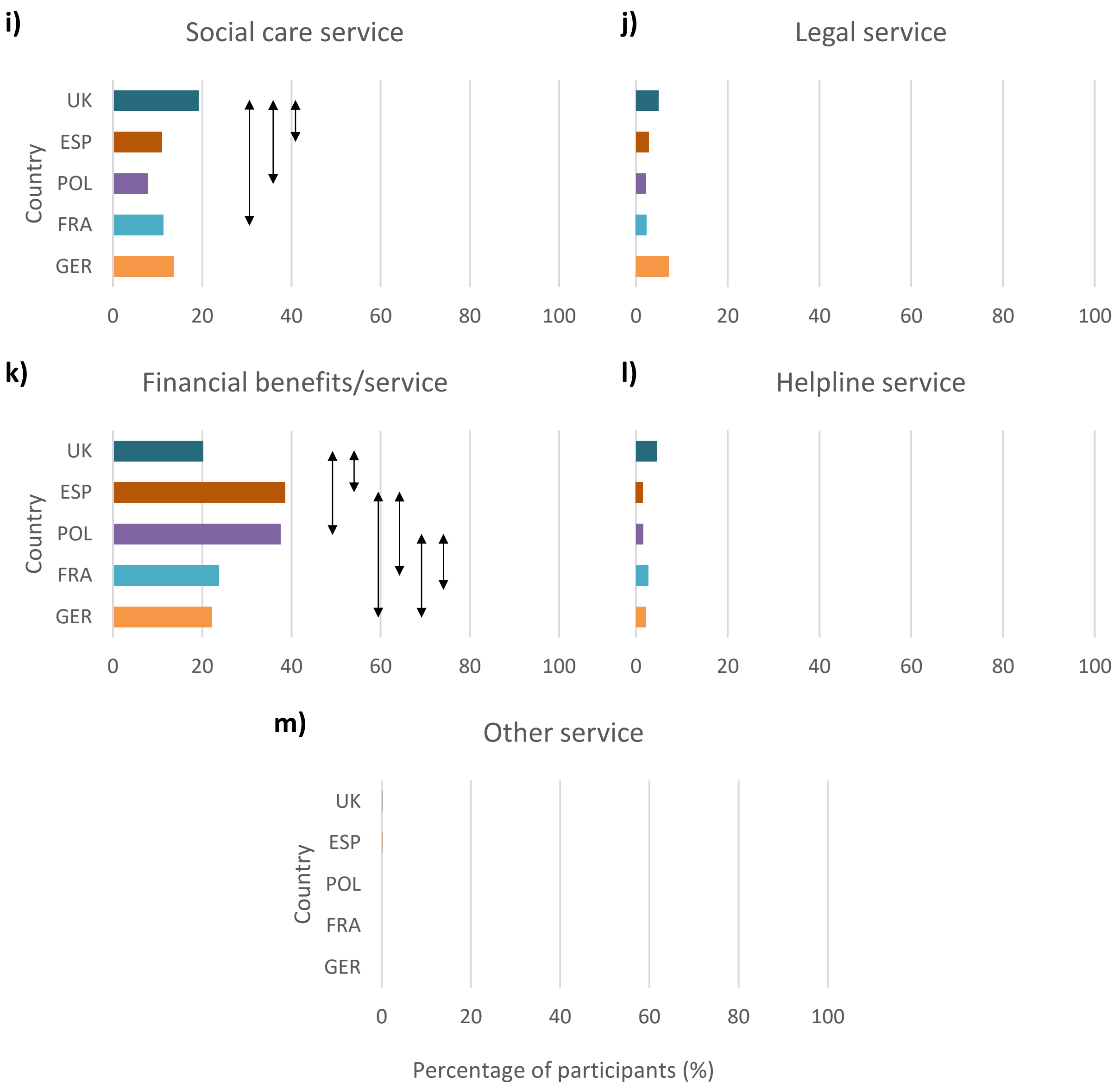

Supplementary Fig. 23. Percentage of participants, by country, indicating access less than two years before survey completion to **a)** needs assessment services, n=285, **b)** therapy services, n=622, **c)** mental health services, n=594, **d)** information/referral services, n=257, **e)** educational services, n=519, **f)** housing services, n=140, **g)** medical services, n=657, **h)** employment services, n=117, **i)** social care services, n=257, **j)** legal services, n=70, **k)** financial benefits/services, n=495, **l)** helpline services, n=55 and **m)** other services not listed in the survey, n=3. Arrows indicate significant ($p < .05$) two-proportion z-test results, suggesting significant differences between respective countries.

Supplementary Figure 24

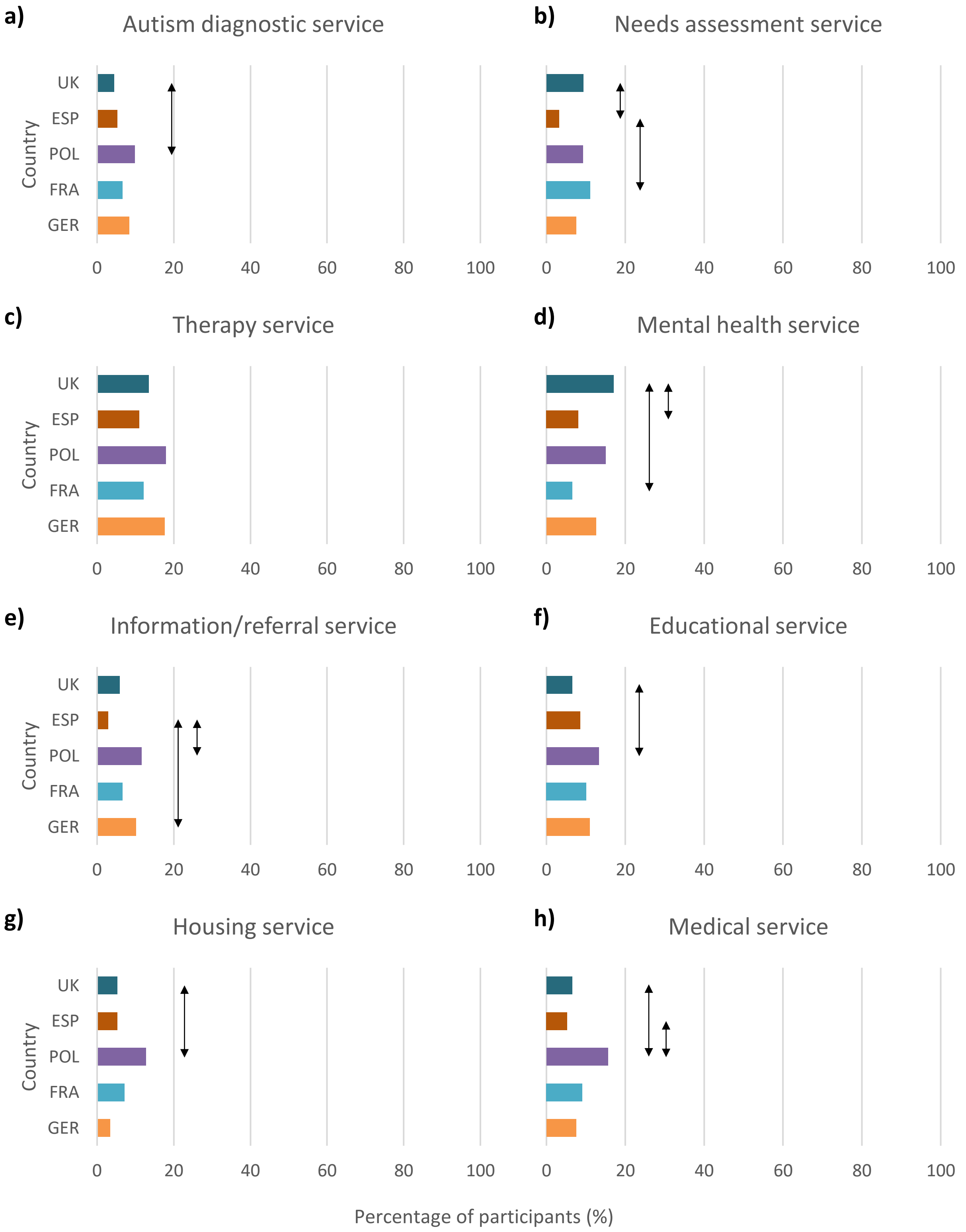

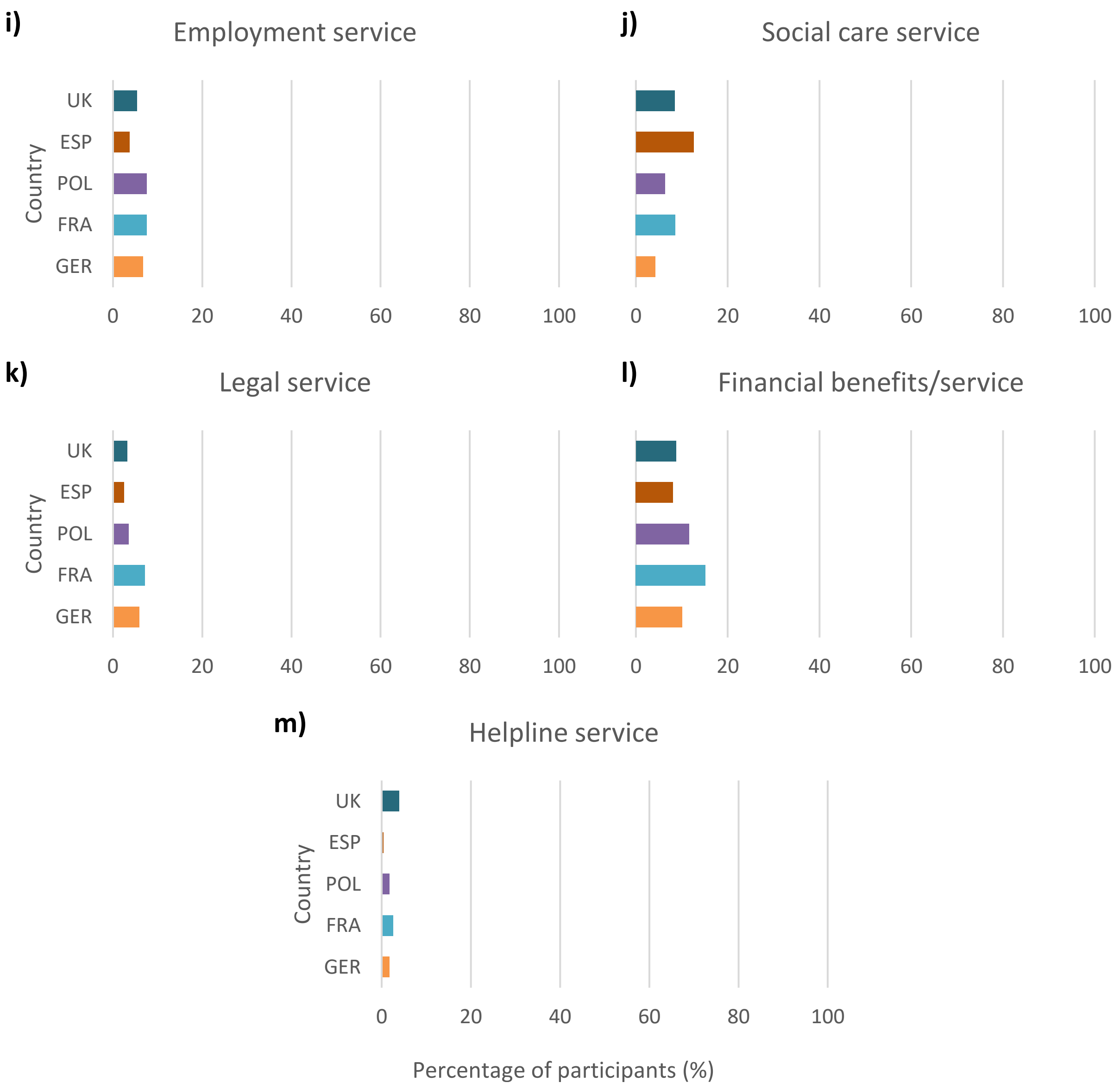

Supplementary Fig. 24. Percentage of participants by country indicating unsuccessful attempts at access less than two years before survey completion to **a)** autism diagnostic services, n=83, **b)** needs assessment services, n=119, **c)** therapy services, n=195, **d)** mental health services, n=191, **e)** information/referral services, n=92, **f)** educational services, n=122, **g)** housing services, n=89, **h)** medical services, n=112, **i)** employment services, n=82, **j)** social care services, n=122, **k)** legal services, n=55, **l)** financial benefits/services, n=142 and **m)** helpline services, n=38. Arrows indicate significant ($p < .05$) two-proportion z-test results, suggesting significant differences between respective countries.
